# Eye Tracking and AI-Generated Content: A Systematic Literature Review of Visual Attention, Cognitive Processing, and User Engagement

**DOI:** 10.3390/jemr19050097

**Published:** 2026-09-04

**Authors:** Hakile Resulbegoviq, Valentina Hlebec, Mirjana Pejić Bach, Irina Stamatović

**Affiliations:** 1 Faculty of Social Sciences, University of Ljubljana, 1000 Ljubljana, Slovenia; valentina.hlebec@fdv.uni-lj.si; 2 Institute Damar, Bulevar Crnogorskih Serdara 5, 81000 Podgorica, Montenegro; 3 Department of Informatics, Faculty of Economics and Business, University of Zagreb, 10000 Zagreb, Croatia; mirjana.pejicbach@gmail.com; 4 IEDC Bled School of Management, Prešernova Street 33, 4260 Bled, Slovenia; 5 Faculty of Commercial and Business Sciences, Lava 7, 3000 Celje, Slovenia; 6 HELP–Hilfe zur Selbsthilfe, Bulevar Knjaza Danila 13/15, 81000 Podgorica, Montenegro; stamatovic@help-ev.de

**Keywords:** eye tracking, generative artificial intelligence, AI-generated content, visual attention, cognitive processing, user engagement, synthetic media, systematic review

## Abstract

Generative artificial intelligence increasingly produces text, images, feedback, summaries, advertisements, synthetic faces, and audiovisual content evaluated alongside human-produced material. This systematic review synthesized comparative eye-tracking evidence on visual attention, cognitive processing, and engagement with AI-generated content. Searches of Scopus, Web of Science, and PubMed yielded 896 records; 23 studies met the eligibility criteria and contributed 778 participants in the review-relevant eye-tracking components. The evidence covered textual, static visual, audiovisual, and interactive outputs. Across heterogeneous designs and tasks, no modality-independent gaze pattern emerged. AI-generated material sometimes attracted more focal inspection, sometimes received less task-relevant attention, and often redistributed gaze across interface elements. Where supported by task characteristics or complementary outcomes, longer viewing was more often associated with processing difficulty, uncertainty, or checking than with preference. Generated summaries supported learning in some settings, whereas realistic synthetic media remained difficult to identify despite focused inspection. Methodological appraisal identified recurrent limitations in sampling, stimulus matching, confounder control, eye-tracking reporting, and documentation of model versions, prompts, generation settings, and output selection. Observed gaze differences were context-dependent and varied with modality, task, comparator, source belief, expertise, output quality, and measurement choices. Standardized reporting and stronger links between gaze and functional outcomes are needed for cumulative inference.

## 1. Introduction

### 1.1. Background and Rationale

Users increasingly encounter content before they know, or can reliably determine, whether it was produced by a person or generated by an artificial intelligence system. An explanation in a learning platform, an image in an advertisement, a translated subtitle, a medical visual, or a response in an online search environment may appear fluent, coherent, and professionally produced while concealing or blurring its computational origin. This uncertainty has become consequential because generative artificial intelligence has developed from a specialized computational capability into a general-purpose infrastructure for producing and transforming information. Contemporary generative systems create text, images, audio, code, and multimodal outputs, while large language models and related applications generate summaries, recommendations, conversational responses, translations, subtitles, explanations, and feedback. These outputs are increasingly encountered not as isolated demonstrations of technological capability, but as functional components of education, online search, healthcare communication, advertising, journalism, creative work, social media, and professional decision-making. In communication and marketing contexts, AI is also increasingly integrated with neuromarketing and social-media analytics to examine how technology-related perceptions, communication processes, and user responses interact [1]. In these environments, users may read an AI-generated explanation, evaluate a synthetic image, respond to automated feedback, or interact with an AI-produced answer without necessarily knowing how, or to what extent, the material was generated or computationally modified. Generative AI has therefore become not only a production technology, but also a consequential part of the human information environment [2].

The increasing realism and communicative fluency of generated outputs complicate conventional distinctions between artificial and human-produced content. In some modalities, synthetic materials can closely approximate authentic examples; state-of-the-art AI-generated faces, for instance, have been shown to be difficult for observers to distinguish from photographs of real people [3]. Comparable challenges arise when users encounter generated writing, artwork, advertisements, or manipulated audiovisual material. Yet perceptual resemblance at the surface level does not establish cognitive or experiential equivalence: content that appears fluent, realistic, or professionally produced may still elicit different patterns of attention, trust, verification, and engagement. Technical assessments of output accuracy, realism, or model performance are therefore insufficient for understanding how generated content functions once it is encountered by human users.

Human responses to AI-generated content may depend on both the observable properties of the material and the beliefs surrounding its origin. Linguistic irregularities, visual artifacts, semantic inconsistency, or mismatches between elements may prompt users to inspect regions or reread uncertain information. At the same time, source expectations, disclosure labels, prior familiarity with AI, and general trust in automated systems may shape how the same material is evaluated. Actual provenance and perceived provenance must therefore be distinguished: an AI-generated output may be processed as human-made, while authentic content may be treated with suspicion because it appears artificial. Research on AI-generated artwork, for example, indicates that visual attention and evaluation may be associated not only with the true authorship of a stimulus but also with whether observers believe it was created by a human or an AI system [4].

These distinctions matter because differences in gaze do not map onto a single psychological process. Rapid orientation toward an AI-generated element may reflect perceptual salience or an immediately noticeable anomaly, while sustained viewing may indicate interest and engagement, or, just as plausibly, uncertainty, processing difficulty, or an attempt to verify authenticity. Repeated inspections can represent meaningful information integration or unresolved confusion; concentrated gaze may reflect successful identification of a relevant feature or a narrow search for errors; broad scanpaths may indicate exploration or inefficient search. The same eye-movement measure can therefore carry different meanings across tasks and modalities: a longer dwell time on an AI-generated image during an aesthetic task cannot be interpreted in the same manner as prolonged fixation on a suspected deepfake during a detection task, or on automated feedback during a learning activity.

Much of the early evidence on human responses to AI-generated content has relied on questionnaires, interviews, authenticity ratings, liking judgments, and trust scales. These measures remain indispensable because they capture conscious evaluations, perceived usefulness, and subjective experience, but they are incomplete indicators of the processes that unfold during exposure. Retrospective reports depend on what participants notice, remember, and are able or willing to articulate, and may be affected by reconstruction, limited introspective access, social-desirability tendencies, and cues about the purpose of an experiment [5,6]. Such retrospective assessment is also poorly suited to reconstructing the moment-to-moment fluctuations in attention through which users inspect, compare, reject, or revisit information [7]. Self-report should therefore not be displaced but triangulated with behavioral and physiological measures that capture processes closer to the time at which they occur. This need for methodological triangulation is also consistent with research in social neuroscience and electronic commerce, where physiological and behavioral data have been proposed as complementary sources for understanding processes that may not be adequately captured through conventional self-report methods alone [8].

Eye tracking offers one such complement by recording the spatial and temporal organization of visual behavior. Fixation location and duration indicate which elements receive overt visual attention and for how long; time to first fixation reveals the speed with which a region attracts gaze; revisits and regressions identify reinspection; and scanpaths, gaze transitions, and entropy-based measures characterize how attention moves across a display. In reading research, eye movements have long been used to study the allocation of processing effort across words, clauses, and discourse, while research on scenes and visual search has demonstrated their value under more complex viewing conditions [9,10]. Applied to AI-generated content, these measures can reveal whether users inspect generated and comparison materials differently, which features they prioritize, and whether viewing becomes more concentrated, fragmented, or exploratory.

Pupillometry adds a further dimension by capturing task-evoked changes in pupil diameter associated with cognitive effort and attentional state. Pupil responses are affected by luminance, arousal, fatigue, and momentary lapses of attention, and cannot be treated as an unqualified measure of cognitive load [11,12]. More broadly, eye tracking does not provide direct access to thought, comprehension, or intention. The long-standing association between fixation behavior and information processing is theoretically useful, but it does not license a simple eye–mind equivalence in every task; valid interpretation instead requires explicit attention to experimental instructions, content modality, comparison condition, areas of interest, data-processing decisions, and complementary subjective or physiological measures [13].

The emerging literature illustrates both the value of this approach and the difficulty of synthesizing it. Eye-tracking studies of AI-generated content are distributed across human–computer interaction, cognitive psychology, education, communication, advertising, medical imaging, and misinformation research, examining, among other things, the reading of AI- and human-authored text, visual attention to AI-created art, processing of AI versus teacher feedback, and detection of deepfake videos. These studies differ markedly in participant populations, tasks, stimuli, comparison conditions, eye-tracking devices, and dependent measures.

This heterogeneity makes broad conclusions particularly vulnerable to conceptual overreach. A finding that an AI-generated stimulus received longer total fixation duration cannot be generalized as evidence that AI content is “more engaging” unless the task, comparator, and related outcomes support that interpretation. Higher pupil dilation cannot automatically be described as cognitive overload, and more concentrated gaze on a synthetic face may reflect either successful anomaly detection or uncertainty-driven scrutiny. Direct comparisons between AI- and human-generated artifacts also address a different question from comparisons between AI feedback and teacher feedback, or between GenAI search responses and conventional results. These forms of evidence can be synthesized within a common review, but they should not be treated as methodologically interchangeable.

Consequently, the literature should not be reduced to the binary question of whether AI-generated content attracts more or less attention. A more informative synthesis must determine which aspect of attention was measured, during what task, toward what type of content, against which comparison condition, using which gaze indicator, and in association with which cognitive, behavioral, or evaluative outcome—while also considering whether the underlying studies provide adequate methodological reporting, valid stimulus matching, and reproducible descriptions of how the AI content was generated. Without such distinctions, apparent cross-study patterns may reflect differences in measurement and design rather than stable differences in human responses to AI-generated material. A systematic review is therefore needed to organize this interdisciplinary evidence, evaluate the strength and interpretability of its methods, and identify the conditions under which AI-generated content is processed differently from relevant human, authentic, or non-AI alternatives.

### 1.2. Objectives and Scope of the Review

This systematic literature review synthesizes comparative empirical evidence on the use of eye tracking, gaze-based measures, and pupillometry to examine human responses to AI-generated content. Its central focus is content encountered and processed by human participants, rather than artificial intelligence as a general technical system. Eligible studies involve participants directly viewing, reading, evaluating, or interacting with identifiable AI-generated outputs, and must include a comparison condition drawn from human-generated, teacher-generated, professionally produced, authentic, or non-AI material. Eligible AI-generated outputs include textual passages, summaries, feedback, chatbot responses, images, visual designs, artworks, synthetic faces, medical images, videos, deepfakes, and multimodal materials.

Interactive studies are included only when participants process a discernible generated output and gaze-based measures are used to examine attention, reading, evaluation, or engagement during that exposure. The review excludes research in which AI is used only to classify or analyze eye-tracking data, in which gaze functions solely as input to an adaptive system, or in which the primary contribution is model development without substantive analysis of human responses to generated content. It also excludes evaluations of AI systems that do not expose participants to identifiable generated outputs, studies that mention eye tracking without analyzing gaze-based responses, and comparisons limited to different AI models, prompts, or generated variants without a valid human, authentic, or non-AI condition.

The comparative focus is intended to move beyond cataloguing where AI and eye tracking have been used together. The review examines whether, how, and under what conditions visual attention, cognitive processing, and engagement differ across AI-generated and comparison conditions, considering content modality, participant characteristics, experimental task, eye-tracking methodology, cognitive and behavioral outcomes, methodological quality, and the reproducibility of generated stimuli. It distinguishes direct content comparisons—such as AI-generated versus human-generated text, images, or videos—from studies of AI-generated feedback or assistance, and from interactive exposure to chatbot responses, GenAI search results, or task-embedded support. These evidence types are related but represent different forms of exposure and warrant separate interpretation.

The review makes four principal contributions. First, it maps an interdisciplinary and methodologically diverse evidence base. Second, it synthesizes the conditions under which AI-generated and comparison content produce similar or different patterns of attention, processing, and engagement. Third, it critically evaluates how gaze measures are translated into claims about internal cognitive and affective processes. Fourth, it identifies weaknesses in eye-tracking reporting and in the documentation and reproducibility of AI-generated stimuli. The remainder of the article first establishes the theoretical and conceptual foundations for interpreting eye movements in relation to AI-generated content, then describes the systematic review methodology, presents the synthesized findings, and discusses methodological implications, evidence gaps, and priorities for future research.

## 2. Theoretical and Conceptual Grounding

### 2.1. Eye Tracking as a Measure of Visual Attention

Eye tracking records the spatial and temporal organization of gaze and provides process-level evidence about overt visual attention. Because high-acuity vision is concentrated around the fovea, gaze shifts indicate which display elements are selected for detailed inspection and when. This relation is probabilistic rather than absolute: attention can be allocated covertly, and looking at an element does not establish that it was understood, trusted, remembered, or preferred [10,14].

Fixations are relatively stable periods between saccades during which visual information can be sampled in detail. Common measures include fixation count, mean and total fixation duration, fixation rate, dwell time, and time to first fixation. Together, these measures describe repeated sampling, sustained allocation of gaze, and initial orientation; their meaning depends on the task, stimulus, and accompanying evidence [10,13].

Saccades and scanpaths characterize how gaze moves through a display, while AOI transitions, revisits, regressions, and entropy measures describe switching, reinspection, and the dispersion or predictability of gaze. They are useful for describing search and information-sampling strategies, but do not by themselves distinguish efficient exploration from processing difficulty [15].

Pupillometry extends eye tracking to psychophysiological response. Task-evoked pupil changes have been associated with effort, cognitive control, arousal, and uncertainty, but are also affected by luminance, fatigue, baseline differences, and measurement artifacts. Pupil responses should therefore be interpreted as context-sensitive convergent indicators rather than direct measures of cognitive load [11].

### 2.2. Visual Attention and Information Processing

Visual attention prioritizes parts of a complex information environment through an interaction of bottom-up properties, such as contrast, motion, and novelty, and top-down factors, including goals, expectations, prior knowledge, and source beliefs. Eye tracking makes the temporal organization of this selection observable by distinguishing initial orientation, sustained inspection, search, and reanalysis.

Visual search and reanalysis are goal-directed. In AI-related tasks, users may inspect content for anomalies, compare generated output with source information, or revisit earlier regions. These patterns describe where and when information is sampled; verification should be inferred only when supported by task requirements or complementary evidence [3,16,17].

Information integration requires coordinated processing across sources or modalities. Transitions and scanpaths can show the sequence in which information regions are consulted, but frequent switching may reflect either successful coordination or split attention. Comprehension must therefore be established through performance evidence rather than inferred from gaze alone [18,19].

### 2.3. Cognitive Processing of AI-Generated Content

AI-generated content may be perceptually fluent while remaining uncertain in provenance, accuracy, or reliability. Users must therefore interpret what an output communicates and evaluate whether it is coherent, authentic, and appropriate for the task.

Processing fluency refers to the ease with which information is perceived or interpreted and should be distinguished from accuracy and epistemic reliability [20,21]. Cognitive load theory distinguishes demands arising from task complexity from avoidable demands created by presentation or coordination requirements [22]. Generative systems may reduce search or organization demands while introducing additional evaluative and coordination demands; this represents a candidate redistribution of effort rather than a universal effect.

Uncertainty and anomaly detection are particularly relevant when generated content appears human-like but its origin or reliability is unclear. Actual, disclosed, perceived, and inferred provenance may diverge, so attention can be shaped by source beliefs independently of objective origin. Localized scrutiny does not itself establish successful detection, and gaze duration is not a direct measure of trust [3,23].

Comprehension requires successful construction and application of meaning. Efficient or prolonged viewing may accompany either successful understanding or processing difficulty, so claims about comprehension should be grounded in outcomes such as recall, transfer, revision quality, or task accuracy.

### 2.4. User Engagement with AI-Generated Content

Engagement is multidimensional and includes visual, behavioral, evaluative, and learning components rather than a single observable response [24]. Visual engagement concerns the allocation of gaze; behavioral engagement concerns interaction and persistence; evaluative engagement concerns judgments of credibility, usefulness, authenticity, or satisfaction; and learning engagement concerns comprehension, transfer, feedback uptake, revision, and continued task involvement.

These dimensions are related but non-equivalent. Fixation, dwell, and revisits show that content received visual resources but do not establish positive interest or acceptance. Evaluative engagement should be assessed through ratings or choices, and learning engagement through performance or behavior rather than inferred from gaze alone.

### 2.5. Limits of Inference from Eye Movements

Eye tracking provides temporally detailed behavioral evidence but does not directly reveal internal mental states. The eye–mind assumption is useful because gaze often accompanies information processing, yet a fixation does not establish comprehension, trust, preference, memory, or successful integration, and attention may also shift without direct fixation [9].

A central interpretive problem is many-to-one mapping: the same gaze pattern may arise from different psychological processes, and different gaze strategies may support the same outcome. Longer fixation, for example, may reflect interest, effort, or difficulty, while revisits may reflect either productive comparison or unresolved processing [13].

Interpretation must therefore consider task instructions, stimulus properties, comparator conditions, participant expertise, source disclosure, response accuracy, and complementary measures. Measurement choices—including sampling rate, calibration, event detection, AOI construction, preprocessing, data loss, and visual controls—can also alter gaze estimates and limit cross-study comparability.

Triangulation provides the strongest basis for inference. Gaze findings become more persuasive when they converge with task accuracy, reaction time, comprehension, behavioral choice, ratings, interviews, logs, or physiological indicators. Accordingly, the review distinguishes observable gaze processes from candidate psychological interpretations and functional outcomes, retaining higher-level interpretations only when supported by task structure or convergent evidence.

## 3. Materials and Methods

### 3.1. Review Design and Reporting Framework

This systematic review was prepared in accordance with PRISMA 2020 and the Synthesis Without Meta-analysis (SWiM) reporting principles where quantitative pooling was not appropriate [25,26]. A comparative PECOS framework guided eligibility: the population comprised human participants; the exposure was direct viewing, reading, evaluation, or task-based processing of identifiable AI-generated content; eligible comparators were human-generated, professionally produced, teacher-generated, authentic, real, traditional, or no-AI/no-augmentation conditions; outcomes included eye-tracking, gaze, or pupillometric measures and associated cognitive, evaluative, behavioral, or learning outcomes; and eligible designs were original comparative empirical studies. The completed PRISMA 2020 Checklist is provided in Supplementary Table S13.

### 3.2. Information Sources and Search Strategy

Three bibliographic databases were searched: Scopus, Web of Science Core Collection, and PubMed. The final searches were conducted on 2 July 2026 and returned 503, 381, and 12 records, respectively (896 records in total). Searches were restricted to English-language publications from 2015 onward. Database-specific syntax combined terms for eye tracking and gaze with terms for generative AI, AI-generated content, synthetic media, deepfakes, large language models, and relevant content formats. Search dates, limits, record counts, and the available search-strategy documentation are provided in Supplementary Table S2A,B.

### 3.3. Eligibility Criteria

Studies were eligible when they reported original comparative empirical research with human participants who directly encountered identifiable AI-generated content; included an eligible human-generated, professional, teacher-generated, authentic/real, traditional, or no-AI/no-augmentation comparator; used eye tracking, gaze tracking, or pupillometry as an outcome measure; were published in English from 2015 onward; and were available as peer-reviewed journal articles or full conference papers.

Exclusion criteria were AI used only as an analytic method; no identifiable AI-generated exposure; no human participants; no eye-tracking or gaze outcome; gaze used only as system input; no eligible comparator; non-empirical publication; or an ineligible publication type, including preprints without a peer-reviewed version, theses, posters, extended abstracts, late-breaking reports, book chapters, and non-peer-reviewed reports. Detailed operational definitions are provided in Supplementary Table S1.

### 3.4. Study Selection

The database searches yielded 896 records. After 305 duplicates were removed, 591 unique records were screened by title and abstract, and 491 were excluded. One hundred reports were sought for retrieval; 27 were not retrieved, leaving 73 reports for full-text assessment. Fifty full texts were excluded for the following reasons: no eligible comparator (*n* = 28), eye tracking did not assess direct processing of AI-generated content (*n* = 12), ineligible publication type or status (*n* = 5), no direct exposure to AI-generated content (*n* = 3), or non-empirical/no eligible human-participant study (*n* = 2). Twenty-three studies were included.

**Figure 1:**
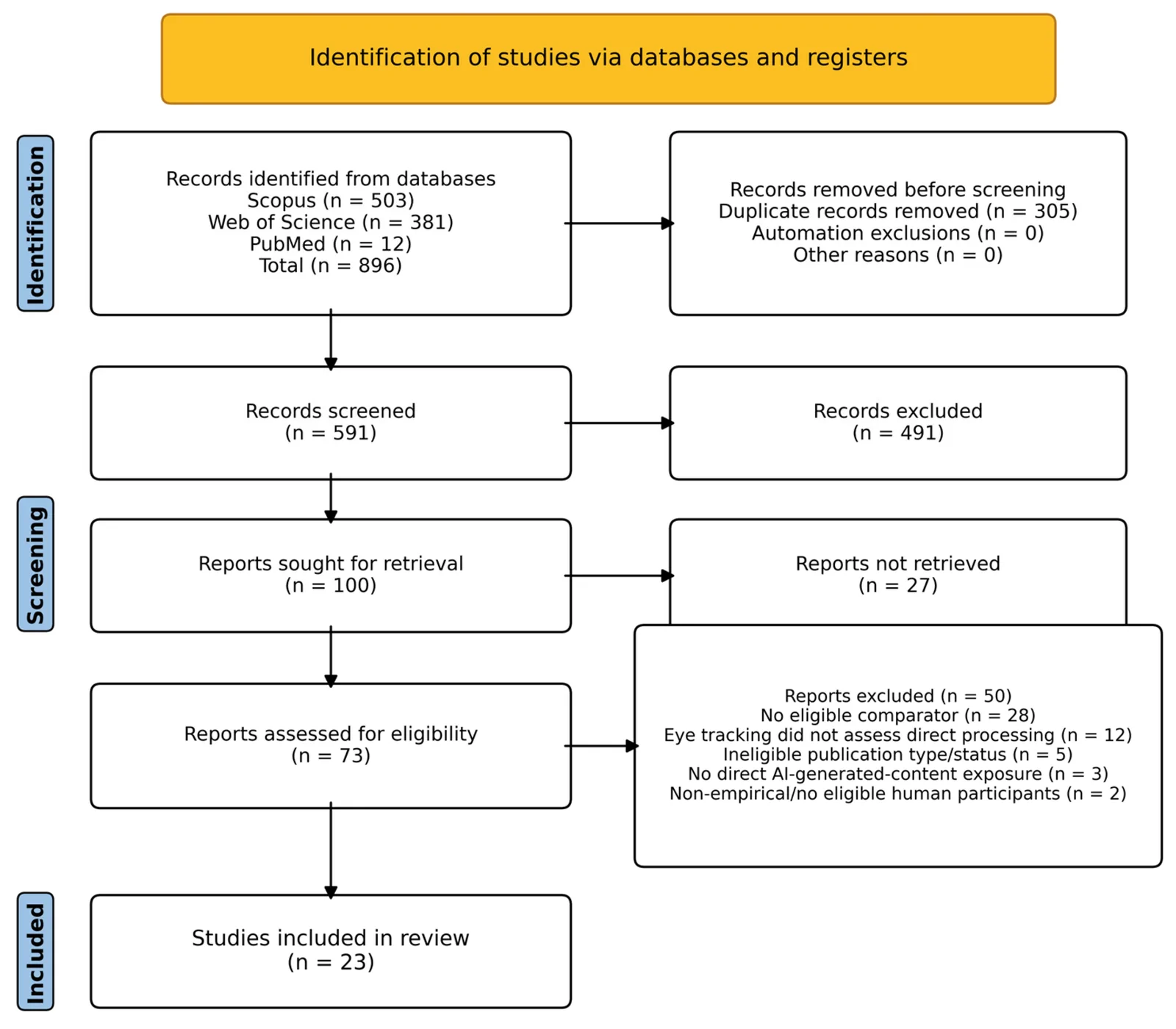
PRISMA 2020 flow diagram of study identification, screening, eligibility assessment, and inclusion.

Two reviewers (H.R. and I.S.) conducted the screening using the predefined eligibility criteria. As an initial calibration exercise, both independently screened the same first 50 records and compared their decisions with the senior methodological supervisor (V.H.) to ensure consistent application of the criteria. H.R. and I.S. then independently screened the remaining titles and abstracts and assessed the retrieved full texts. Disagreements and borderline cases were resolved through discussion and consensus, with guidance from V.H. when needed. No automation tools were used, and no formal inter-reviewer agreement statistic was calculated. The complete study-selection process is shown in Figure 1. Aggregate reasons for full-text exclusion are summarized in Supplementary Table S3A, and a citation-level list of all 50 reports excluded following full-text assessment is provided in Supplementary Table S3B.

### 3.5. Data Extraction

A standardized extraction framework captured bibliographic characteristics, country and domain, study design, sample, participant expertise, AI-generated content and model, comparator, disclosure, task, eye-tracking hardware, sampling rate, calibration, event detection, AOIs, preprocessing, gaze outcomes, cognitive and engagement outcomes, moderators, and principal findings. For multi-study reports, the eye-tracking component relevant to this review was the unit of extraction.

### 3.6. Methodological Appraisal

General study-design quality was appraised with the Mixed Methods Appraisal Tool (MMAT), version 2018 [27]. Each report was assigned the appropriate MMAT design category. Mixed-methods studies were assessed across qualitative, quantitative, and integration components. Ratings were recorded as Yes, No, or Can’t tell; no summed score or exclusion threshold was used.

To complement MMAT, a purpose-built matrix assessed technical reporting completeness and potential validity concerns specific to eye tracking. It recorded device and sampling characteristics; calibration, validation, and drift procedures; event-detection algorithms and thresholds; AOI/ROI definition; preprocessing; missing-gaze handling; participant and trial exclusions; pupil processing; visual controls; statistical correction; and data/code availability.

The eye-tracking matrix was retained separately from MMAT because it is not a validated risk-of-bias instrument. Missing reporting was treated as limited methodological transparency and did not demonstrate that a procedure had not been performed. A separate matrix assessed AI-stimulus reporting and reproducibility, including provider/model, model version, prompts, generation settings and date, output selection, human editing, comparator matching, and material availability. Complete study-level judgments are provided in Supplementary Tables S5 and S10.

A descriptive sensitivity-oriented comparison was used to incorporate technical reporting completeness into interpretation. Studies fully reporting at least five of seven core domains—device/model, sampling rate, calibration, validation/drift control, event-detection algorithm, numerical event thresholds, and AOI/ROI definition—were classified as having higher technical reporting completeness. This review-specific threshold was not treated as a validated quality score, risk-of-bias cutoff, or exclusion criterion; all studies remained in the synthesis.

### 3.7. Data Synthesis

The review used structured narrative and thematic synthesis. Before synthesis, we assessed whether quantitative pooling was feasible within four prespecified subgroups: reading and language processing, authenticity and detection, learning and information integration, and professional and creative tasks. Studies were compared with respect to design, comparator, participant population, task, gaze-metric definition, AOI, unit of analysis, and availability of compatible numerical statistics. No subgroup contained enough studies with conceptually equivalent outcomes and the summary statistics required to derive a common standardized effect; meta-analysis was therefore not performed.

Findings were organized by content modality, task, comparator, metric family, psychological or behavioral outcome, general study-design quality, technical reporting completeness, and AI-stimulus reproducibility. Direct provenance comparisons were interpreted separately from studies of generated feedback, summaries, search, and task-embedded assistance, because these evidence classes do not estimate a single underlying “AI-generated content effect.” Null and mixed findings were retained. Psychological interpretations of fixation, dwell, pupil response, or transitions were used only when supported by task structure or convergent behavioral, subjective, performance-based, or physiological evidence.

## 4. Results

### 4.1. Overview of Included Studies

A total of 23 studies met the eligibility criteria and were included in the final synthesis. Because several reports contained more than one empirical study or methodological component, the characteristics reported below refer specifically to the eye-tracking component relevant to this review. The included literature was published between 2020 and 2026 and was strongly concentrated in the most recent years. One study was published in 2020, one in 2023, three in 2024, 12 in 2025, and six in 2026. Thus, 18 of the 23 studies (78.3%) appeared in 2025 or 2026, indicating that empirical interest in gaze-based responses to AI-generated content has expanded rapidly alongside the wider availability of generative-AI systems. Seventeen studies (73.9%) were published as journal articles, whereas six (26.1%) were full conference papers. Detailed study characteristics are presented in Table 1.

The geographical distribution of the evidence was diverse but uneven. A participant or data-collection setting could be identified for 18 studies, 11 of which were situated in East Asia. Five studies were conducted in China, two in the Republic of Korea, one across China and South Korea, one in Hong Kong SAR, one across China and Macao SAR, and one in Japan. European settings included Germany, the United Kingdom, Norway, Denmark, and Poland, while one study was conducted in the United States and one was conducted in Australia. Five reports did not explicitly identify the participant or data-collection country. The geographical profile therefore reflects a marked concentration in East Asian and European research environments, with more limited representation from North America, Australia, and other world regions.

The studies were also distributed across several disciplinary traditions. Seven were situated primarily within visual-perception and synthetic-media research, including work on generated faces, AI images, manipulated images, AI art, and deepfake detection. Six originated in education and learning, examining generated summaries, instructional feedback, help-seeking, and generative search. Five addressed design, communication, or creative work, including logo evaluation, health advertising, cultural illustration, architectural façades, and AI-supported design ideation. Four focused on language, translation, or text-entry tasks, including AI-authored passages, subtitles, transcription contexts, and post-editing. One study was situated in medical imaging and radiology and examined radiologists’ responses to synthetic chest X-rays. This disciplinary breadth confirms that eye tracking is being used to investigate AI-generated content across both basic perceptual research and applied professional contexts.

Across the review-relevant eye-tracking components, the studies included a combined total of 778 participants. Final analyzable sample sizes ranged from 10 to 82 participants, with a median of 30 and a mean of 33.8. Eleven studies (47.8%) primarily recruited students, learners, or trainees, including university students, school-aged learners, translation trainees, and second-language learners. Nine studies (39.1%) used general-adult or mixed samples, although several of these were still recruited through universities or professional networks. Two studies recruited design- or culture-related specialists, and one involved domain experts—practicing radiologists. Overall, the evidence base relied heavily on relatively small convenience and student samples, while only a small proportion of studies examined expert or population-specific groups.

Based on the dominant form of AI-generated content encountered by participants, 10 studies (43.5%) were classified as primarily static visual, seven (30.4%) as textual or linguistic, four (17.4%) as multimodal or interactive, and two (8.7%) as audiovisual or deepfake-video studies. These primary categories were mutually exclusive for descriptive mapping. However, several studies contained more than one content type. For example, educational systems combined generated text with images or mind maps, while AI-supported design environments incorporated both conversational responses and generated visual stimuli. The content-specific synthesis therefore uses overlapping categories where necessary rather than treating all AI-generated outputs as equivalent exposures.

To increase study-level transparency, Table 2 provides a compact feature matrix for all 23 included studies. The matrix combines the principal characteristics needed to interpret the comparative evidence—review-relevant sample size, AI-generated exposure and model, comparator, eye-tracking system, sampling frequency, and core gaze indicators. More detailed information on study design, stimulus construction, calibration, preprocessing, AOIs, data-quality procedures, and outcome definitions remains available in Supplementary Tables S4–S6.

### 4.2. Types of AI-Generated Content

The included studies examined heterogeneous generated outputs, ranging from fixed textual or visual stimuli to dynamically produced responses embedded within learning, search, and professional workflows. The synthesis therefore grouped evidence by both content form and functional role. Direct provenance comparisons addressed processing of the artifact itself, whereas task-embedded studies examined generated output as feedback, assistance, or information within broader activities. These evidence classes were interpreted separately.

#### 4.2.1. Textual Content

Eleven studies exposed participants to at least one identifiable form of AI-generated textual content. The most direct text-origin comparison was conducted by Ilyas et al. [28], who compared the reading of LLM-generated literary passages with passages written by human authors. Abu-Rayyash and Lacruz [29] examined GPT-4o-generated Arabic subtitles in comparison with professionally translated subtitles while participants viewed the same television episode. Komninos et al. [30] investigated LLM-generated conversational context surrounding a mobile transcription task, comparing this condition with a baseline in which the same type of text-entry task was completed without generated dialogue.

Generated summaries represented another important textual category. Morita et al. [31] examined text summaries, generated images, and image summaries as augmentations to digital reading materials, whereas Yuan et al. [32] compared LLM-generated text and mind-map summaries with a video-learning condition that contained no pedagogical-agent summary. These studies treated generated text as instructional scaffolding rather than as a standalone artifact. Their outcomes consequently concerned not only attention to the generated material but also comprehension, retention, navigation between information sources, and cognitive load.

Two studies focused specifically on AI-generated feedback. Cao et al. [33] compared learners’ visual attention to multidimensional writing feedback generated by a large language model with feedback provided by teachers. Cosentino et al. [34] compared personalized GPT-4 feedback with human-instructor feedback during an embodied mathematics activity. In both studies, the generated text functioned as pedagogical guidance embedded within an ongoing task rather than as material viewed in isolation.

Interactive conversational output was represented by Chen et al. [35], in which university students received either ChatGPT-generated or human-expert help while revising an English essay. Xu et al. [36] examined generated answers produced through a retrieval-augmented GenAI search system and compared them with results obtained through conventional web search. Yao et al. [37] studied GPT-4-generated reasoning and revision suggestions during translation post-editing, contrasting AI-assisted and traditional workflows. Finally, Fu et al. [38] included GPT-4.1 responses as part of an AI-supported design-ideation condition. Together, these studies broadened the textual evidence beyond fixed reading stimuli to include feedback, search results, professional suggestions, and collaborative dialogue.

The textual literature was therefore methodologically diverse. Some studies compared generated and human-authored texts directly, while others evaluated the incremental effect of adding generated summaries, feedback, or assistance to a task. These forms of exposure answer related but distinct questions and were consequently retained as separate synthesis groups.

#### 4.2.2. Static Visual Content

Static visual output was the most extensively represented form of AI-generated content. Ten studies used generated visual material as the primary experimental stimulus, while three additional multimodal studies embedded generated images or visual summaries within broader educational or design tasks.

Generated faces and human-like characters were examined by Cha et al. [39] and Bozkir et al. [16]. Cha et al. compared real facial photographs with two-dimensional and three-dimensional AI-generated character images across depicted age categories. Bozkir et al. compared real faces with synthetic faces produced by PGGAN, StyleGAN, and StyleGAN2. Both studies used gaze behavior to investigate how participants inspected realistic and computer-generated facial stimuli, although their tasks differed: one emphasized free viewing and socio-perceptual evaluation, whereas the other involved realism and authenticity judgments.

Other studies examined creative or communicative visual artifacts. Guan et al. [40] compared AI-generated and human-created brand logos and additionally manipulated whether creator identity was disclosed. Zhou and Kawabata [4] examined AI-generated and human-made landscape paintings, combining free viewing with aesthetic ratings and provenance judgments. Liu et al. [41] compared AI-generated and human-drawn Tibetan cultural illustrations, with particular emphasis on cultural authenticity and symbolic interpretation. Yfantidou et al. [42] evaluated AI-generated images in bowel-cancer screening advertisements alongside hand-drawn and stock-image alternatives. Yun and Kim [43] compared AI-generated biophilic façade designs with a photographic image of an existing automated-retail storefront.

Medical and authenticity-related visual content formed another subgroup. Wong et al. [44] examined radiologists’ responses to diffusion-generated and real chest X-rays during authenticity and diagnostic judgments. Cartella et al. [45] used partially manipulated images created through diffusion-based inpainting and instruction-guided editing and compared them with their original versions. Osińska et al. [46] compared AI-generated images with authentic photographs across several categories, including faces, architecture, art, vehicles, landscapes, and animals.

Generated static visuals also appeared in three studies whose primary modality was coded as multimodal or interactive. Morita et al. [31] integrated generated story images and image summaries into digital reading materials. Fu et al. [38] used Midjourney-generated visual stimuli during concept development in addition to conversational AI support during ideation. Yuan et al. [32] presented generated mind maps as visual summaries accompanying instructional video. These task-embedded visuals were analytically distinguished from passive or evaluative image-viewing studies because gaze behavior reflected interaction with a broader information environment rather than processing of a single isolated image.

#### 4.2.3. Audiovisual and Deepfake Content

Only two studies directly examined AI-generated or synthetically manipulated video as the primary content modality. Gupta et al. [47] developed an eye-tracking and EEG database in which participants viewed real and deepfake videos and classified each video as authentic or manipulated. Holmes et al. [48] similarly compared non-experts’ gaze behavior while they attempted to identify authentic and face-replacement deepfake videos. Both studies focused on dynamic authenticity detection, but they differed in stimulus source, experimental procedure, and the granularity of the reported gaze measures.

The limited number of direct video studies contrasts with the broader static-image literature. Although Abu-Rayyash and Lacruz [29] used audiovisual material, the generated element in that study was the subtitle text rather than the video itself. Likewise, the video-learning studies included human-produced instructional videos supplemented by generated summaries or search responses. These studies were therefore classified according to the generated textual or instructional output rather than as evidence concerning AI-generated video. This distinction prevented the audiovisual category from being inflated by studies in which generative AI altered only a textual layer of an otherwise non-generated video.

#### 4.2.4. Interactive and Task-Embedded AI Outputs

Nine studies examined generated output as part of an interactive system or ongoing task. In these studies, the object of analysis was not simply a completed AI artifact; instead, generated content shaped how participants searched, learned, revised, designed, or responded to information.

Educational support constituted the largest function within this category. Morita et al. [31] provided generated summaries and images during reading, Yuan et al. [32] introduced generated text or mind-map summaries during video learning, and Cosentino et al. [34] delivered personalized formative feedback during embodied mathematics tasks. Cao et al. [33] examined GenAI writing feedback within an academic-writing interface, whereas Chen et al. [35] studied interactive ChatGPT help during essay revision. These systems differed substantially in timing and function: some supplied generated information before or alongside primary content, while others responded to learner performance or help requests.

Interactive AI was also used in creative and professional workflows. Fu et al. [38] investigated generated dialogue and visual inspiration during design ideation and concept development. Yao et al. [37] embedded GPT-4 reasoning and revision suggestions within translation post-editing. Komninos et al. [30] introduced generated conversational context around a mobile text-entry task. Xu et al. [36] allowed learners to submit queries to a GenAI search system that returned dynamically generated answers during a science-learning activity.

These interactive studies were retained because participants directly encountered identifiable generated outputs and gaze was used to examine their processing or the resulting allocation of attention. Nevertheless, they form a conceptually distinct evidence group. In direct content comparisons, gaze differences can be linked more closely to the properties or presumed origin of the stimulus. In interactive studies, gaze is additionally shaped by interface layout, task sequence, user goals, timing of the generated response, and the functional role of AI assistance.

### 4.3. Study Designs, Tasks, and Comparison Conditions

Most studies employed repeated-measures designs. Sixteen of the 23 studies (69.6%) used within-subject or repeated-measures comparisons, allowing the same participants to encounter multiple content origins, presentation conditions, or stimulus types. This approach was particularly common in studies comparing real and synthetic faces, images, artworks, medical images, and videos, as well as in reading and post-editing experiments. Five studies (21.7%) used between-subject designs. These included comparisons of AI and professional subtitles, generated and no-summary learning conditions, GenAI and traditional search, GenAI and human-instructor feedback, and ChatGPT versus human-expert help. Two studies (8.7%) used hybrid designs that combined within- and between-subject elements. Bozkir et al. [16] combined repeated exposure to real and synthetic faces with primed and unprimed participant groups, whereas Fu et al. [38] used different comparison structures across the ideation and development phases.

The experimental tasks were similarly varied. Six studies (26.1%) used free-viewing or evaluative tasks, commonly followed by ratings of interest, aesthetics, trustworthiness, cultural authenticity, or behavioral intention. A further six studies (26.1%) required participants to detect or classify content as real, synthetic, manipulated, or AI-generated. These authenticity tasks were used with faces, chest X-rays, static images, and deepfake videos. Four studies (17.4%) involved reading, transcription, or subtitle viewing, including AI-authored text, generated subtitles, interactive reading materials, and LLM-supported text-entry contexts. Three studies (13.0%) were primarily learning experiments involving generated summaries, feedback, or search responses. Two studies (8.7%) examined feedback processing or help-seeking during writing and revision. Creative ideation and concept development were the primary tasks in one study, while one study focused on professional translation post-editing. Information seeking was represented most directly by the GenAI-versus-traditional-search experiment, although search and source comparison also formed secondary components of several learning and feedback tasks.

All included studies contained an eligible comparison condition, but the comparator structure differed substantially. Nine studies (39.1%) compared AI-generated output with material or assistance produced by humans, professionals, teachers, or domain experts. This category included AI versus human-created logos, illustrations, artworks, and literary passages; LLM versus professional subtitles; GenAI versus teacher feedback; ChatGPT versus human-expert help; and AI versus human design collaboration. Eight studies (34.8%) compared synthetic, generated, or AI-manipulated content with real or authentic content. These comparisons involved faces, architectural façades, chest X-rays, static images, and deepfake videos.

Four studies (17.4%) compared an AI-generated support condition with a no-AI, no-augmentation, or traditional baseline. These included GenAI reading augmentation, LLM-generated transcription context, GPT-assisted post-editing, and generated pedagogical summaries. Importantly, these comparisons did not necessarily contrast AI-generated content with human-authored content. For example, the baseline in Morita et al. [31] removed the generated augmentation, but the underlying narratives were themselves generated. One study compared GenAI search with conventional web search, and one compared AI-generated health-advertising imagery with both hand-drawn and stock-image alternatives.

The comparator taxonomy therefore separated direct provenance comparisons from intervention and interface comparisons. AI-versus-human and synthetic-versus-real studies provided the clearest evidence about how content origin related to visual processing. Comparisons with no-AI support, traditional search, or alternative presentation formats instead addressed whether adding generated assistance changed attention, effort, or engagement within a task. Maintaining this distinction was necessary because these designs do not estimate the same underlying effect, even when all are broadly framed as comparisons involving AI-generated content. Accordingly, the review does not interpret variation across these design classes as evidence for or against a unitary effect of AI provenance.

### 4.4. Eye-Tracking Devices and Methodological Procedures

The included studies used a heterogeneous set of eye-tracking technologies, although Tobii systems clearly predominated. Twelve of the 23 studies employed a Tobii device or platform, including the Tobii Pro Spectrum, Pro Fusion, Pro Nano, TX300, T120, 4C, and mobile eye-tracking glasses (Tobii, Stockholm, Sweden). Four studies used an EyeLink 1000 Plus system (SR Research Ltd., Ottawa, ON, Canada). The remaining studies used RealEye (RealEye Sp. z o.o., Poznań, Poland), RealEyes (Realeyes OÜ, Tallinn, Estonia), iMotions WebET (iMotions A/S, Copenhagen, Denmark), GazePoint GP3 HD (Gazepoint Research Inc., Vancouver, BC, Canada), or Pupil Labs Pupil Invisible glasses (Pupil Labs GmbH, Berlin, Germany), while two reports did not identify the eye-tracker manufacturer or model. This distribution reflects the methodological diversity of the corpus, ranging from high-frequency laboratory systems to scalable webcam-based platforms suitable for remote data collection.

Eighteen studies relied on screen-based eye tracking, including both conventional remote systems positioned below a monitor and tower-mounted systems used with head stabilization. Three studies collected gaze data remotely through participants’ webcams. Abu-Rayyash and Lacruz [29] used the RealEye platform to examine Arabic subtitle processing, Yfantidou et al. [42] used RealEyes to evaluate health advertisements, and Yun and Kim [43] used iMotions WebET to study responses to AI-generated architectural façades. Two studies used mobile or head-mounted glasses: Cosentino et al. [34] recorded gaze during an embodied mathematics task using Tobii glasses, whereas Holmes et al. [48] used Pupil Invisible glasses during dynamic deepfake-video detection. The use of mobile systems enabled gaze to be recorded during embodied and moving-viewpoint tasks, but it also required more complex mapping of gaze onto changing scenes or dynamic areas of interest.

Sampling frequency was explicitly reported in 15 studies and ranged from 30 to 1200 Hz, with a median reported rate of 150 Hz. High-frequency laboratory recording was used in several cognitively demanding tasks: Cha et al. [39] recorded at 1200 Hz, Yao et al. [37] at 1000 Hz, Wong et al. [44] at 500 Hz, Bozkir et al. [16] at 600 Hz, and Gupta et al. [47] at 300 Hz. Lower-frequency systems were more common in remote and applied studies, including 30 Hz webcam tracking, 60 Hz Tobii Pro Nano recording, and 90 Hz Tobii 4C or other Tobii-based systems. In eight studies, sampling frequency was not reported. One report also contained an internal methodological ambiguity: Cosentino et al. [34] described gaze recording at 50 Hz but separately stated that pupil diameter was captured at 250 Hz. Such inconsistencies limit the reproducibility and cross-study comparability of the evidence.

Calibration procedures varied considerably in both detail and stringency. Reported approaches ranged from a one-point calibration in the mobile embodied-learning study to five-, nine-, 13-, and 39-point procedures in screen-based and webcam-based studies. Several studies supplemented calibration with drift correction or pretrial fixation checks. Yfantidou et al. [42], for example, applied a nine-point calibration, a 500 ms prestimulus drift check, and recalibration when drift exceeded 1°. Yao et al. [37] combined 13-point calibration with a one-point drift check before each trial, while Zhou and Kawabata [4] used nine-point calibration and validation, central-fixation checks, and recalibration following breaks or failed fixation checks. Other reports merely stated that calibration had been conducted without describing the number of points, validation accuracy, or acceptance rule. A smaller subset did not report calibration procedures at all.

The identification of fixations and saccades was similarly inconsistent. Nine studies reported an event-detection method or a sufficiently explicit threshold. Velocity-threshold identification was used in several studies, although the threshold varied substantially. Bozkir et al. [16] applied an I-VT threshold of 30°/s and a minimum fixation duration of 60 ms, whereas Abu-Rayyash and Lacruz [29] used a considerably higher velocity threshold in their web-based subtitle study. Yfantidou et al. [42] used I-VT with a 30°/s threshold, merged spatially and temporally adjacent fixations, and required a minimum dwell period for an AOI visit. Morita et al. [31] implemented a custom dispersion-based procedure requiring nine consecutive 90 Hz samples within a predefined pixel radius, producing a minimum fixation duration of 100 ms. Ilyas et al. [28] used the I2MC algorithm with adaptive interpolation and filtering, while Gupta et al. [47] used the EyeMMV procedure. In many other studies, fixations and saccades were reported as outcomes without disclosing the event-detection algorithm or numerical thresholds. This variation is consequential because the same raw gaze stream may produce different fixation counts and durations under different detection rules.

Areas of interest were formally defined in 16 studies. Their scope reflected the substantive diversity of the review. Reading and learning studies distinguished between main text, generated images, summaries, subtitles, video regions, feedback panels, and answer options. Applied interface studies separated AI responses, human feedback, source and target text, simulation areas, search areas, or writing interfaces. Visual-content studies defined regions around faces, eyes, mouths, logos, slogans, people, objects, façades, and signage. In contrast, seven studies analyzed whole-stimulus gaze distributions, heatmaps, saliency maps, or participant-reported features without establishing formal AOIs. The resulting measures are therefore not directly equivalent: dwell within a prespecified subtitle or slogan AOI represents a different analytical unit from whole-image fixation entropy or a qualitative heatmap.

Data-quality procedures were one of the least consistently reported methodological features. Only five studies stated explicit numerical quality or exclusion thresholds, while eight reported partial or qualitative criteria and ten provided no clearly reproducible gaze-quality rule. The most detailed remote studies specified limits for calibration accuracy, track loss, valid-gaze proportion, or data completeness. Yfantidou et al. [42], for example, excluded trials with more than 40% track loss and participants with more than 25% total track loss. Yun and Kim [43] required calibration accuracy within 1.3° and at least 90% gaze and facial-data completion. Abu-Rayyash and Lacruz [29] classified recordings by effective sampling and usable-fixation proportions, ultimately excluding 25 out of 107 participants. Ilyas et al. [28] excluded implausibly brief trials, trials lacking a recognizable multiline reading pattern, and participants with too few valid trials. Elsewhere, authors stated only that unstable, incomplete, or low-quality recordings were removed, without providing numerical thresholds. Overall, the corpus shows substantial technological sophistication but uneven reporting of the procedures needed to evaluate data reliability and reproduce the analyses. A corpus-level summary of the principal eye-tracking methods and reporting characteristics is provided in Table 3.

### 4.5. Eye-Tracking Measures Used

Fixation-based measures dominated the literature. Fixation count was analyzed in 14 studies, total fixation time or dwell time in 14, and mean fixation duration in 12. Two additional studies reported fixation rate rather than raw fixation count. These variables were used to operationalize several different constructs, including sustained attention, reading effort, information sampling, visual search, and cognitive demand. However, their interpretation varied by task. In subtitle and feedback studies, longer or more frequent fixations were generally treated as evidence of increased processing demand or rereading. In visual-design and advertising studies, the same measures were sometimes interpreted as sustained interest or engagement. Some studies normalized fixation measures by text length, word count, total viewing time, or AOI exposure, whereas others reported raw counts or durations. This variation reduced direct comparability even where the nominal outcome was the same.

Early attentional orientation was examined much less frequently. Only three studies used time to first fixation as a formal outcome, and one additional study compared first-fixation distributions. TTFF was used to assess the speed with which participants oriented toward subtitles, logos, slogans, or other task-relevant regions. Wong et al. [44] instead compared spatial patterns associated with the first fixation on authentic and synthetic radiographs. The scarcity of orienting measures means that the evidence base is much stronger regarding sustained allocation of attention than regarding the speed of initial attentional capture.

Measures of reinspection were also relatively uncommon. Four studies quantified revisits or re-fixations, with one further study using them qualitatively, and two studies reported regressions or AOI backtracking. These variables were used to infer repeated inspection, verification, rereading, or unresolved processing. Their operational definitions nevertheless differed. Some studies counted returns to a fixed AOI, others counted re-fixations within a dynamic video or image region, and reading studies calculated backward movements or backtracking relative to word count. Consequently, “reinspection” represented a family of related but non-identical behaviors rather than a single standardized outcome.

Scanning behavior was measured using several approaches. Six studies analyzed saccade amplitude or length, and two reported saccade count. Five quantitatively analyzed scanpaths or gaze trajectories, while three additional studies used scanpath patterns qualitatively. Two studies quantified AOI transitions, with another incorporating transition behavior into multimodal process coding. Entropy measures appeared in two studies: Gupta et al. [47] used fixation entropy to characterize dispersion during video viewing, whereas Cartella et al. [45] calculated entropy over saliency maps to compare the concentration of gaze on real and AI-edited images. Three studies also used saliency-map comparison metrics. These included correlation or similarity indices and Kullback–Leibler divergence between fixation distributions.

Pupillometric outcomes were comparatively rare. Three studies analyzed pupil diameter, and two examined task- or baseline-corrected changes in pupil size. Fu et al. [38] calculated baseline-corrected pupil diameter and cumulative pupil dilation during design ideation, Cha et al. [39] compared average pupil diameter across real and AI-character conditions, and Ilyas et al. [28] examined mean pupil size during AI- and human-authored text reading. Cosentino et al. [34] derived a wavelet-based pupillary activity measure intended to index cognitive load. Although these studies used pupil responses as indicators of effort, arousal, or attentional demand, reporting of luminance control, baseline procedures, blink handling, and sampling was inconsistent. Pupillometric findings were therefore interpreted alongside task and gaze evidence rather than as direct measurements of cognitive load.

Several studies introduced less conventional or study-specific indices. Abu-Rayyash and Lacruz [29] used the K-coefficient to distinguish more focal from more ambient viewing during subtitle processing. Cosentino et al. [34] calculated an information-processing index from fixation and saccade characteristics to describe global versus local processing. Other derived measures included AOI fixation-duration ratios, gaze-proportion measures, start–end variation ratios, baseline-corrected pupil indices, gaze-trajectory length, per-word normalized fixation measures, and process-transition probabilities. Heatmaps were presented in 14 studies, although they primarily served as descriptive visualizations rather than independent inferential outcomes. No study in the final corpus analyzed interpersonal or intersubject gaze alignment. The absence of gaze-alignment measures represents a notable methodological gap, particularly given the inclusion of interactive feedback, help-seeking, and human–AI collaboration contexts.

Overall, the included literature relied heavily on conventional fixation measures but showed limited convergence around a core reporting set. The use of numerous custom indices enriched individual analyses but complicated synthesis. Measures bearing similar labels were not always calculated in equivalent ways, and different studies used the same metric to support claims about attention, effort, engagement, uncertainty, or comprehension. For this reason, findings were synthesized by metric family and task context rather than by assuming that a given gaze measure represented the same psychological construct across all studies.

To complement the narrative synthesis, we also compared the distribution of eye-tracking metric families across the four evidence subgroups (Table 4). This comparison further illustrates why a common standardized effect size was not estimable. Even within the same substantive subgroup, studies rarely converged on an identical set of gaze outcomes. Reading and language studies were relatively concentrated around fixation and saccade measures, whereas authenticity and detection studies used a wider combination of fixation, scanpath, entropy, saliency, and reinspection measures. Learning studies more often analyzed gaze distribution and cross-AOI transitions within complex interfaces, while professional and creative studies predominantly used fixation-based measures. Thus, quantitative differences across subgroups primarily concerned the availability and operationalization of outcome families rather than repeated estimates of a common effect.

### 4.6. Visual Attention to AI-Generated Content

Across the heterogeneous study designs included in this review, no single modality-independent pattern of visual attention emerged. This absence of convergence should not be interpreted as evidence that AI-generated content inherently lacks a characteristic gaze response; it may also reflect substantial differences in modality, task, comparator, stimulus properties, interface structure, and the functional role of the generated content across studies. Where eye-tracking procedures were incompletely reported, particularly for sampling, calibration, event detection, or AOI specification, fine-grained differences in fixation, dwell, transition, or scanpath measures were interpreted more cautiously. Some studies found that generated content attracted longer or more concentrated inspection; others observed reduced attention, redistribution across interface regions, or no reliable difference from the comparison condition. The direction of the effect depended on the content modality, task, comparator, source disclosure, stimulus characteristics, and gaze metric. Direct provenance comparisons also produced different patterns from studies in which generated content functioned as feedback, a summary, search support, or an interactive aid.

#### 4.6.1. Initial Attention Capture

Evidence concerning initial attentional capture was limited. Three studies reported TTFF and one additional study analyzed first-fixation distributions. Collectively, these findings did not support the proposition that AI-generated content consistently attracts gaze more rapidly than human-generated or authentic content.

Guan et al. [40] found that initial attention to logos varied with innovation, semantic relevance, and whether creator identity had been disclosed. The results did not indicate a simple early-orienting advantage for AI-generated logos. In the subtitle study by Abu-Rayyash and Lacruz [29], TTFF to the subtitle region did not differ significantly between GPT-4o and professional subtitles, although participants in the AI condition oriented more rapidly toward the picture region. Yfantidou et al. [42] reported the clearest disadvantage for AI-generated visual content: AI images delayed first fixation on the slogan relative to hand-drawn and older stock-image conditions. Wong et al. [44], using first-fixation maps rather than TTFF, found highly similar initial gaze distributions for real and synthetic chest X-rays.

The small number of studies, different AOIs, and divergent tasks preclude a general conclusion about rapid attention capture. Early gaze appeared to be influenced less by AI provenance alone than by the salience and task relevance of specific elements. Moreover, a shorter TTFF would indicate faster orientation but would not, by itself, demonstrate preference, engagement, or superior processing.

#### 4.6.2. Sustained Attention

Sustained-attention findings were markedly heterogeneous. Several studies found greater fixation or dwell on AI-generated content. GPT-4o subtitles elicited more fixations, longer mean fixation duration, longer total subtitle-viewing time, and a larger share of gaze than professional subtitles [29]. Komninos et al. [30] similarly found that adding LLM-generated conversational context substantially increased fixation duration and count across the prompt and response regions compared with a baseline transcription task. Generated summaries also attracted considerable attention: in Yuan et al. [32], text-summary and mind-map conditions accounted for 14.8% and 12.4% of fixation time, respectively, compared with only 0.8% in the corresponding region of the no-summary control. Osińska et al. [46] found that fixations on AI images were approximately 20 ms longer than those on authentic photographs, although fixation frequency and most other global measures did not differ by origin.

In other studies, AI-generated content received less sustained attention. Yfantidou et al. [42] found that AI-generated health-advertising images produced shorter slogan dwell, fewer fixations, and fewer revisits than hand-drawn advertisements. Liu et al. [41] observed that human-drawn Tibetan illustrations attracted more sustained attention to culturally diagnostic details, whereas AI illustrations sometimes diverted attention toward vivid colors, supporting characters, or text. During AI-assisted translation post-editing, participants spent less fixation time and made fewer fixations on the source text than during traditional post-editing, especially under the lighter editing brief [37]. In the FakeET study, real videos produced more fixations and re-fixations than deepfakes, despite little difference in mean fixation duration [47].

A further group of studies reported null or strongly conditional effects. Bozkir et al. [16] found no reliable real-versus-GAN difference in free-viewing time, with task phase and priming influencing gaze more consistently than actual origin. Cao et al. [33] found no significant difference between GenAI and teacher feedback in fixation time or fixation count; feedback dimension was more influential than source. Zhou and Kawabata [4] reported that actual AI versus human authorship did not affect dwell or fixation count, whereas paintings believed to be human-made received longer viewing. Holmes et al. [48] found no difference in fixation duration or count between authentic and deepfake videos. Cha et al. [39] identified selective age-by-image-type effects rather than an overall AI-character advantage.

Interactive studies often showed redistribution rather than a simple increase or decrease in attention. GenAI search reduced sustained attention to the lecture video while increasing switching between the search and simulation regions [36]. GenAI feedback redirected gaze toward correct options and the projected number line, whereas human feedback generated more attention to the provider, task text, and incorrect options [34]. AI-supported design ideation produced a more balanced distribution between the assistant and the design canvas [38]. Collectively, these results show that the question of whether AI content “holds attention longer” is underspecified. The answer depends on which AOI is considered and whether longer viewing reflects productive engagement, cognitive effort, uncertainty, or verification.

#### 4.6.3. Reinspection and Verification

Evidence of reinspection and verification was less extensive than fixation-based evidence and did not reveal a consistent AI-origin effect. Formal revisit or re-fixation measures were reported in only four studies, regressions or backtracking in two, and several additional studies inferred verification from prolonged or repeated inspection without calculating a dedicated revisit variable.

AI-generated advertising images produced fewer returns to the slogan than hand-drawn advertisements, indicating weaker repeated engagement with the central message [42]. In contrast, selected AI-generated façade variants generated higher revisit counts in particular façade or signage AOIs, although highly complex combinations could divert attention away from functional signage [43]. Gupta et al. [47] found more re-fixations on real videos overall, but difficult deepfakes generated more re-fixations than easier deepfakes, suggesting that reinspection was partly driven by detection difficulty rather than synthetic origin alone. Osińska et al. [46] reported descriptive revisit differences across participant subgroups and image categories, but no general AI-versus-real revisit effect.

Reading and feedback studies provided similarly conditional evidence. Cao et al. [33] found no difference in backtracking between GenAI and teacher feedback; instead, completeness and grammar/vocabulary feedback generated more returns than structure and coherence feedback. Regression proportions in the AI- and human-text conditions of Ilyas et al. [28] were descriptively similar and were not reported as a significant origin effect. In the subtitle study, greater fixation count and dwell under GPT-4o were interpreted as increased rereading and scrutiny, but revisits were not measured directly. Synthetic chest X-rays produced slightly longer maximum fixations and more divergent prolonged-fixation saliency maps, suggesting localized scrutiny rather than greater overall viewing [44].

Interactive studies suggested that verification may also take the form of cross-referencing. Cosentino et al. [34] found that learners receiving GenAI feedback more frequently coordinated attention between the correct option and the number line, which the authors interpreted as a verification-oriented strategy. In Chen et al. [35], participants receiving human-expert help more often returned to earlier assistance, whereas ChatGPT users more often requested immediate follow-up help or progressed without an explicit evaluation phase. These findings are consistent with differences in the organization of checking behavior, although gaze transitions or revisits alone cannot establish that verification occurred.

Accordingly, the available gaze evidence does not establish that users systematically verify AI-generated content more often. Reinspection patterns varied with task difficulty, content quality, available comparison information, and task instructions, but verification should be inferred only when repeated inspection was linked to an explicit checking task, behavioral evidence, or complementary measures.

#### 4.6.4. Scanpath Organization

Scanpath and gaze-organization findings revealed two partially contrasting patterns. In several direct AI comparisons, gaze became more concentrated or focal. Fu et al. [38] reported shorter gaze-trajectory length during AI-supported ideation than during designer-to-designer collaboration, suggesting a narrower and more economical search pattern. Abu-Rayyash and Lacruz [29] observed an increase in the subtitle K-coefficient from 0.10 for professional subtitles to 0.30 for GPT-4o subtitles, indicating a shift toward more focal, detail-oriented processing. Gupta et al. [47] found that deepfake videos produced shorter, more compact, face-centered scanpaths with lower entropy, whereas authentic videos elicited broader exploration of the scene. Cartella et al. [45] likewise found lower saliency-map entropy for AI-edited images than for genuine images, indicating more spatially concentrated viewing.

A different pattern emerged when generated content served as an interactive or organizational aid. Mind-map summaries produced networked global-to-local exploration, while text summaries produced more linear top-to-bottom reading and the no-summary control remained concentrated on the video [32]. GenAI search increased cross-area saccades between the simulation and search regions, reflecting more distributed information switching than conventional search [36]. GenAI feedback in embodied mathematics produced more balanced global and local processing and a different AOI-transition structure than human feedback [34]. Morita et al. [31] found that generated text summaries supported broader coverage of the main document, whereas generated images and image summaries increased cross-region gaze shifts. Chen et al. [35] identified a more nonlinear help-seeking pathway in the ChatGPT condition, although gaze was incorporated into multimodal process coding rather than a conventional scanpath metric.

Other studies found limited origin-related differences. Bozkir et al. [16] observed longer saccades during evaluative rating than during free viewing, but this pattern applied across real and GAN faces. Wong et al. [44] found similar initial and final saliency distributions for real and synthetic X-rays, with greater divergence only in maps based on the longest and shortest fixations. Holmes et al. [48] reported no difference in saccade count between authentic and deepfake videos. Osińska et al. [46] found no general origin effect on saccade magnitude, although experienced participants used fewer, longer fixations and shorter saccades while searching for generation artifacts. Ilyas et al. [28], by contrast, found larger saccadic amplitudes during AI-text reading, together with shorter but more frequent fixations.

These results do not support a universal “AI scanpath”. More focal gaze sometimes reflected efficient access to an AI aid, but in authenticity tasks it could also reflect narrowed inspection of suspected artifacts. Broader or more fragmented gaze sometimes represented inefficient search, yet in multimodal learning it could indicate active integration across sources. Scanpath organization therefore requires interpretation in relation to task goals and information structure rather than along a simple focused-versus-dispersed quality continuum.

#### 4.6.5. Attention to Specific Areas of Interest

Formal AOIs or ROIs were defined in 16 studies, while the remaining seven used whole-stimulus measures, saliency maps, heatmaps, or qualitative feature descriptions. AOI selection closely followed content modality and task demands.

In face and deepfake research, attention was typically analyzed around the eyes, mouth, face, and body. Cha et al. [39] visualized fixation-duration patterns for the eyes and mouth across real, two-dimensional, and three-dimensional characters. Bozkir et al. [16] did not define formal AOIs, but participants frequently reported using the eyes, hair, ears, overall facial appearance, and background as cues when judging synthetic faces. Gupta et al. (2020) [47] contrasted gaze centered on the manipulated face or mouth with attention to other people and the wider scene. Holmes et al. [48] used dynamic AOIs for the eyes, mouth, and body, finding that the eyes attracted the greatest fixation and saccade activity regardless of video authenticity. Osińska et al. [46] used category-specific AOIs, including eyes, mouth and nose, ears, hair, and neck for faces; muzzle, paws, and body for animals; and logos, number plates, wheels, drivers, structural lines, vegetation, and background features for other image categories. These detailed AOI analyses showed that attention to particular features varied with expertise and stimulus category, but no single feature reliably distinguished AI from authentic content.

Textual and educational studies partitioned interfaces into content-specific regions. Abu-Rayyash and Lacruz [29] separated subtitles from the picture area. Morita et al. [31] distinguished the primary document, generated image, text summary, and image summary. Komninos et al. [30] analyzed the generated prompt and response bubbles. Yao et al. [37] compared source- and target-text inspection, while Yuan et al. [32] distinguished the video and generated-summary regions. Xu et al. (2026) divided the learning interface into the lecture video, virtual simulation, and search area. Cao et al. [33] separated GenAI feedback, teacher feedback, and the learner’s original abstract, and further coded structure, coherence, completeness, and grammar/vocabulary feedback. Cosentino et al. (2025) [34] analyzed the feedback provider, task text, correct and incorrect options, and the projected number line. Chen et al. [35] coded gaze within the dialog box, reply messages, essay-writing tool, and reading or essay content. These AOIs allowed interactive studies to show that AI often redistributed attention across task components rather than changing overall viewing time.

Design and communication studies used AOIs tied to persuasive or functional content. Guan et al. [40] defined separate regions for individual AI- and human-created logos and adjacent creator labels. Yfantidou et al. [42] distinguished the slogan, people or faces, and objects within screening advertisements, revealing that image type altered attention to the message rather than merely to the image as a whole. Liu et al. [41] examined faces, Tibetan cultural symbols, supporting characters, and text, although the AOI procedure was incompletely specified. Yun and Kim [43] distinguished the glass façade, side-signage region, and main signage area, showing that visually complex generated designs could increase façade engagement while simultaneously reducing visibility of functional information.

Several studies retained a whole-stimulus approach. Zhou and Kawabata [4] analyzed entire paintings rather than predefined regions, Cartella et al. [45] compared whole-image saliency entropy, and Wong et al. [44] constructed bias and saliency maps over complete radiographs rather than anatomical AOIs. Ilyas et al. [28] treated each passage as a whole reading stimulus. These approaches were appropriate for global distribution or reading-pattern questions but offered less information about which precise content elements accounted for origin-related effects.

Taken together, AOI findings show that the location of attention was often more informative than overall fixation time. AI-generated content did not uniformly receive more or less attention; instead, it frequently changed which elements users prioritized. This included shifting attention between subtitles and imagery, source and target text, feedback and task content, correct and incorrect options, central messages and peripheral design elements, or manipulated facial regions and the broader scene. At the same time, the heterogeneity of AOI definitions, and the absence of formal AOIs in nearly one-third of the studies, limits direct quantitative comparison across modalities.

### 4.7. Cognitive Processing and Cognitive Load

The evidence on cognitive processing was organized into four overlapping domains: reading and language processing; authenticity and detection; learning and information integration; and professional or creative task performance. An important distinction was made between studies that formally measured cognitive load or effort and those that inferred processing demands from gaze behavior alone. Seven studies explicitly operationalized load or effort through instruments such as NASA-TLX, task-specific effort ratings, pupillometry, physiological measures, or multimodal cognitive-load indices. In the remaining studies, fixation duration, rereading, gaze concentration, scanpath organization, or task performance provided indirect process evidence but were not treated as direct measures of cognitive load. Interpretations of cognitive effort based primarily on gaze were given greater weight when the underlying eye-tracking procedures were technically well documented and when gaze findings converged with behavioral, subjective, or physiological measures. Where such convergent evidence was absent, fixation duration, rereading, scanpath concentration, transitions, and pupil responses were described primarily as gaze or processing patterns rather than as direct indicators of cognitive load or effort.

#### 4.7.1. Reading and Language Processing

The language-focused studies showed that AI-generated text did not produce a uniform processing advantage. Instead, its effects depended on output quality, linguistic function, task requirements, and the extent to which readers had to evaluate or integrate the generated material. In the most direct AI-versus-human text comparison, Ilyas et al. [28] found that AI-generated literary passages elicited shorter mean fixations, a higher fixation rate, and larger saccadic amplitudes than human-authored passages. This pattern suggests a distinguishable reading strategy, but not necessarily easier or more effective comprehension. Effects also varied across the authorial styles that the model attempted to imitate, indicating that textual style and readability moderated the relationship between source and eye movements. Although comprehension questions were administered, the corresponding comprehension results were not reported, preventing a direct determination of whether the altered gaze pattern translated into equivalent understanding.

The clearest evidence of increased processing demands emerged in the subtitle study by Abu-Rayyash and Lacruz [29]. Viewers of GPT-4o-generated Arabic subtitles made more fixations, showed longer mean fixation durations, spent more total time in the subtitle region, and allocated a larger proportion of gaze to subtitles than viewers of professionally translated subtitles. The higher K-coefficient further indicated a shift toward more focal, detail-oriented processing. These eye-movement differences converged with poorer performance on comprehension-related outcomes, including factual recall, cultural-reference recognition, and narrative coherence. In this context, greater visual attention to AI-generated subtitles represented additional processing effort and competition with the audiovisual narrative rather than more effective engagement.

Komninos et al. [30] examined LLM-generated conversational context embedded around a mobile transcription task. The presence of generated prompt–response material substantially increased fixation duration and fixation count within the conversational areas relative to the phrase-only baseline. Nevertheless, the eye-tracking experiment did not demonstrate a corresponding improvement or impairment in the principal text-entry outcomes. The generated context therefore changed what participants processed during the task, but evidence that this additional processing improved performance was limited.

Feedback studies produced more differentiated results. Cao et al. [33] found no significant difference in fixation duration, fixation count, or backtracking between GenAI-generated and teacher-generated writing feedback when the feedback addressed the same dimension. Instead, processing demand varied according to feedback content: completeness and grammar or vocabulary feedback attracted more attention and backtracking than structure and coherence feedback. This finding suggests that the semantic and evaluative demands of the feedback mattered more than whether it was generated by AI or a teacher.

Cosentino et al. [34] similarly found that GenAI and human feedback produced different processing strategies rather than a simple difference in total attention. Learners receiving GPT-4 feedback allocated more gaze to the correct answer and the embodied number-line representation, whereas learners receiving human feedback spent more time on the feedback provider, task text, and incorrect options. The GenAI condition also showed a lower pupil-derived cognitive-load index, but this reduction did not produce a significant advantage in task performance. The pattern is therefore consistent with more targeted or verification-oriented processing rather than unequivocally superior learning.

In translation post-editing, Yao et al. [37] found that GPT-4 assistance reduced fixation duration and fixation count on the source text, particularly under a light post-editing brief. However, AI assistance did not reduce total task time and was associated with greater technical editing activity and pause-based effort. Self-reported cognitive effort also did not differ clearly between AI-assisted and traditional conditions. Taken together, these results are consistent with a redistribution of observable task effort away from repeated source-text consultation toward additional editing and coordination activity. Because these processes were captured through multiple gaze, pause, editing, and self-report measures, the interpretation extends beyond gaze alone. The reduction in one form of visual effort therefore did not imply a reduction in overall cognitive work.

Across the language-focused evidence, AI-generated output could accelerate or alter reading patterns, increase local scrutiny, or reduce consultation of existing source material. However, these effects were not consistently associated with improved understanding or efficiency. Output quality, language proficiency, text difficulty, feedback dimension, and the type of editing or reading task were central to interpreting whether gaze differences reflected fluency, difficulty, or strategic redistribution of effort.

#### 4.7.2. Authenticity and Detection

Nine studies examined cognitive processing in relation to synthetic faces, AI-generated or AI-edited images, artificial artworks, medical images, or deepfake videos. The strongest cross-study pattern was not a stable gaze signature of AI-generated content but increasing difficulty as synthetic content became more realistic and fewer visible anomalies remained available.

Bozkir et al. [16] found that participants were more successful in distinguishing real faces from outputs of older generative architectures than from more recent StyleGAN and StyleGAN2 images. Priming participants to expect synthetic content improved some classification outcomes, but task phase and priming influenced fixation rate and saccade length more consistently than actual image origin. Observers therefore did not appear to use a single reliable visual strategy that separated all synthetic faces from real ones. Cha et al. [39] likewise reported selective age-by-image-type effects rather than a general cognitive or attentional cost of AI-generated characters. Greater fixation count and pupil dilation emerged only for particular combinations of depicted age and two- or three-dimensional representation.

Deepfake-video studies reached similar conclusions. In the FakeET study, detection accuracy was highest for real videos, lower for easier deepfakes, and lowest for difficult deepfakes. Deepfake viewing was more spatially concentrated around manipulated facial regions, whereas real videos elicited wider exploration and more re-fixations. Difficult deepfakes also generated more repeated inspection than easier ones. Yet the more focal gaze pattern did not compensate for increasing realism: classification performance still deteriorated as the manipulation became harder to detect. Holmes et al. [48] similarly reported only moderate detection performance and found no significant difference in fixation duration, fixation count, or saccade count between authentic and deepfake videos. Attention to the eyes, mouth, and body also did not reliably distinguish correct from incorrect decisions.

The still-image evidence reinforced the distinction between focused search and successful recognition. Cartella et al. [45] found lower saliency-map entropy for diffusion-edited images than for their original counterparts, indicating that viewers concentrated gaze within a narrower set of regions when inspecting manipulated content. Osińska et al. [46] observed slightly longer fixations on AI-generated images than on real photographs, but most global eye-movement measures did not differ by source. Recognition accuracy varied substantially across image categories, suggesting that the perceptual consequences of generation were not uniform across faces, art, architecture, vehicles, animals, and landscapes. Participants with greater experience in digital graphics or AI imagery tended to use fewer, longer fixations and shorter saccades, consistent with more selective artifact-oriented search.

The art studies demonstrated that actual and perceived provenance could produce different cognitive effects. Zhou and Kawabata [4] found that objective AI versus human authorship did not significantly alter aesthetic ratings or major fixation measures. In contrast, paintings that participants believed were human-made received longer viewing, regardless of their actual origin. Human-made paintings were also identified more accurately than AI-generated paintings. These findings indicate that source belief can organize attention independently of objective provenance. Liu et al. [41] found a different origin-related pattern for Tibetan cultural illustrations: human-drawn images received stronger cultural-authenticity evaluations and more attention to culturally diagnostic symbols, whereas AI-generated illustrations were often valued for fantasy, vividness, and visual innovation. AI familiarity improved source-identification accuracy but did not remove the perceived cultural gap.

In the medical-imaging study, radiologists’ first and last fixation patterns were broadly similar for authentic and diffusion-generated chest X-rays. Synthetic images nevertheless produced slightly longer maximum fixations and greater divergence in saliency maps based on the most prolonged inspections. This pattern suggests additional localized scrutiny of particular regions rather than a wholesale change in diagnostic viewing. Because the report did not fully model radiologist experience, image-level dependence, and diagnostic category, these differences should be interpreted cautiously.

Overall, authenticity judgments became less reliable as generative realism increased, and concentrated gaze on suspected anomalies did not consistently support accurate classification. The evidence therefore does not indicate that people possess a generalizable visual strategy for recognizing AI-generated content. Detection was shaped by generation method, content category, realism, participant expertise, prior expectations, and whether the task required passive viewing, explicit source classification, or professional diagnosis.

#### 4.7.3. Learning and Information Integration

The educational studies examined how generated summaries, feedback, search responses, and conversational assistance influenced comprehension, retention, cognitive load, and the integration of information across multiple interface regions. Generated summaries produced the clearest learning benefits, although the magnitude and mechanism depended on their format.

Morita et al. [31] found that adding AI-generated images, text summaries, or image summaries to digital narratives improved post-reading scores relative to the unaugmented condition, with the largest reported improvement occurring for image summaries. Eye tracking showed that participants differed substantially in the extent to which they used the supplementary content. Summary conditions generally attracted more consistent attention than isolated generated images, while broader document coverage was interpreted by the original authors as potentially supporting structural processing of the narrative; however, the gaze pattern alone does not establish successful information integration. However, these relationships were mostly descriptive, and performance benefits differed according to whether participants preferred textual or visual materials.

More robust evidence was provided by Yuan et al. [32], who compared generated text summaries, generated mind maps, and a no-summary control during video learning. Both generated-summary conditions improved immediate test performance, while the mind-map condition produced the strongest learning and delayed-retention outcomes. It also reduced extraneous load and supported greater germane processing. Eye-tracking patterns differed systematically by format: text summaries produced relatively linear top-to-bottom scanning, whereas mind maps generated networked global-to-local exploration. Time allocated to the summary region was positively associated with learning outcomes. These findings suggest that the benefit of generated support depended not only on the presence of additional content but on whether its visual organization facilitated relations among concepts.

GenAI search produced more qualified benefits. Xu et al. [36] found that students using GenAI search shifted attention more frequently between the search and simulation areas, whereas traditional-search users maintained more sustained attention on the lecture video. GenAI search improved retention, but did not produce a significant transfer advantage, and cognitive-load ratings did not differ between conditions. The resulting pattern suggests that generated answers facilitated access to and organization of task-relevant information but did not necessarily support deeper abstraction or application to new problems.

The feedback studies likewise showed changes in processing without uniform performance gains. GenAI and teacher writing feedback attracted comparable attention when pedagogically constrained to equivalent feedback dimensions, while completeness and language-focused feedback produced greater processing demands regardless of source. In embodied mathematics learning, GPT-4 feedback was associated with lower pupil-derived cognitive load and more attention to correct options and the number-line representation. Nevertheless, task performance did not differ significantly from the human-feedback condition. The generated feedback appeared to change how learners verified and organized information, rather than simply increasing achievement.

Chen et al. [35] extended this pattern to conversational help-seeking. Students receiving ChatGPT assistance followed a more nonlinear and iterative pathway, frequently moving directly from asking for help to processing or implementing the answer. Those receiving human-expert help showed a more sequential pattern that included explicit diagnosis and evaluation of the assistance. ChatGPT users spent a smaller proportion of the process overtly evaluating help and were more likely to seek immediate follow-up assistance. Because the study did not report a corresponding essay-quality or learning endpoint, this more direct pathway cannot be interpreted as more effective learning; it may instead reflect cognitive offloading or reduced monitoring.

Across the educational evidence, AI-generated support was most consistently beneficial when it organized or summarized content in a way that made relationships explicit. Interactive feedback and search systems more often changed information-selection and verification strategies than they improved performance. Retention gains were more common than transfer gains, and lower cognitive load did not automatically correspond to better task outcomes.

#### 4.7.4. Professional and Creative Tasks

Professional and creative studies showed that generative AI redistributed effort across task phases rather than simply removing cognitive demands. In product-design ideation, Fu et al. [38] found that GPT-4.1 assistance reduced self-reported mental demand relative to designer–designer collaboration and produced shorter gaze trajectories and more balanced attention between the assistant and the design workspace. Overall workload and most physiological indicators, however, did not differ significantly. During concept development, both conventional image references and AI-generated visual stimuli reduced workload relative to a no-stimulus control, but their creative contributions differed: generated stimuli were associated more strongly with novelty and elaboration, whereas curated images tended to support resolution and feasibility. The advantage of AIGC was therefore phase-specific, being clearer in divergent exploration than in convergent refinement.

Translation post-editing presented a similar trade-off. GPT-4 assistance reduced source-text fixation effort but did not reduce total time and increased technical editing and pause-related activity. This suggests that the technology removed some search or consultation demands while introducing coordination and judgment costs. Because translation quality was not reported as an outcome, it remains unclear whether this redistribution of effort produced better professional output.

AI-generated logos, advertisements, and architectural designs further illustrated that creative performance and audience processing depended on the properties of the generated artifact. Logo attention was jointly shaped by creator disclosure, innovation, and semantic relevance. AI-generated health-advertising images attracted less attention to the principal slogan than hand-drawn alternatives, indicating that visual novelty or automation did not ensure effective message processing. AI-generated biophilic façades increased visual exploration and positive evaluation under some design combinations, but excessive complexity diverted attention from functional signage. These findings demonstrate that successful generative design depends on maintaining a balance between novelty, semantic clarity, visual complexity, and task-relevant communication.

Across professional and creative contexts, the strongest conclusion is that AI changes the location and composition of cognitive effort. It can reduce search, retrieval, or ideation demands while increasing the need to screen, interpret, verify, and integrate model outputs. Benefits were therefore conditional on workflow phase, task brief, output relevance, and the degree of continued human evaluation.

### 4.8. User Engagement and Related Outcomes

Engagement was operationalized heterogeneously across the studies. Some reports equated engagement with fixation duration, dwell time, or revisits, whereas others examined interaction behavior, source evaluation, task persistence, learning performance, or affective ratings. To avoid treating these outcomes as interchangeable, the synthesis distinguished visual, behavioral, evaluative, and learning engagement. Claims about engagement based on gaze alone were weighted cautiously, particularly in studies with limited technical reporting, and stronger interpretations were reserved for findings supported by both adequately documented eye-tracking procedures and complementary behavioral or evaluative outcomes.

#### 4.8.1. Visual Engagement

Visual engagement was the most frequently represented engagement dimension, but its meaning depended heavily on the task. Greater fixation duration or dwell was observed for GPT-generated subtitles, LLM-generated conversational context, generated summaries, and some AI-generated images. In these studies, however, greater visual attention was often interpreted in the original studies as reflecting increased processing demand, scrutiny, or uncertainty rather than stronger liking; however, such psychological interpretations were retained only where supported by task characteristics or complementary outcomes. The subtitle study provided the clearest example: AI-generated subtitles attracted more gaze but supported poorer comprehension than professional subtitles.

Conversely, AI-generated advertising images received less slogan-directed attention than hand-drawn images, and AI-generated Tibetan illustrations directed gaze less consistently toward culturally diagnostic details than human drawings. Several other studies found no general origin effect, including comparisons of AI and teacher feedback, authentic and deepfake video, actual AI and human art, and real and GAN-generated faces. In interactive systems, AI often redistributed gaze among interface components rather than changing total attention. Thus, visual engagement with AI-generated content ranged from intensified scrutiny to reduced message focus, source-equivalent processing, or task-dependent reallocation.

#### 4.8.2. Behavioral Engagement

Behavioral engagement was reflected in prompting, searching, revising, clicking, answering, selecting, editing, and continuing to work with generated support. GenAI search increased information-search behavior and transitions between search and simulation regions. ChatGPT help produced more direct, iterative help-seeking and more frequent movement from asking to implementing assistance, whereas human-expert help produced more explicit evaluation and review of earlier advice. AI-assisted post-editing changed the distribution of edits and pauses without reducing total completion time. In design tasks, generative support expanded ideation and was associated with different creative outcomes across divergent and convergent phases.

These behavioral effects were not uniformly beneficial. More frequent interaction could indicate productive exploration, but it could also reflect the need to repair, clarify, or repeatedly evaluate AI output. Similarly, rapid progression from receiving to applying ChatGPT assistance may represent efficiency but may also indicate reduced monitoring. Behavioral engagement therefore required interpretation alongside output quality, task performance, and evidence of evaluation.

#### 4.8.3. Evaluative Engagement

Direct measures of evaluative engagement were less common than gaze measures. Formal liking or aesthetic-preference outcomes were reported in three studies, with an additional study providing broader aesthetic appraisals. A direct trustworthiness measure appeared in one study of AI-generated characters; trust and source credibility were discussed in several others but were not always measured as distinct outcomes.

The strongest evaluative finding concerned perceived provenance. In the art study, actual AI or human authorship did not significantly alter aesthetic ratings, but paintings believed to be human-made received longer viewing. Similarly, logo processing changed after creator labels were presented, and the direction of the effect depended on innovation and semantic relevance. These findings indicate that evaluative engagement can be shaped by what participants believe about the source, even when the underlying content or actual provenance does not produce the same effect.

Aesthetic responses also depended on cultural and design quality. Human-drawn Tibetan illustrations were rated more highly for cultural authenticity and emotional depth, whereas AI images were valued for novelty and visual fantasy. AI façade variants produced favorable evaluations when design features were balanced, but increasingly complex combinations created a dissociation between visual exploration and functional clarity. Across these studies, greater gaze did not consistently correspond to stronger liking, trust, or perceived quality.

#### 4.8.4. Learning Engagement

Learning engagement was represented through comprehension, retention, task performance, feedback uptake, and help-seeking. Formal comprehension or learning outcomes were reported in five studies, with one additional reading study collecting but not reporting comprehension results. Generated summaries most consistently supported learning engagement: both text and visual-summary conditions improved learning relative to no-summary controls, with generated mind maps showing particularly strong retention and integration effects.

Other interventions changed engagement without clearly improving performance. GenAI search increased active exploration and retention but did not improve transfer. GenAI mathematics feedback reduced pupil-derived load and altered verification behavior without increasing task scores. GenAI and teacher writing feedback received comparable attention, but the study did not report subsequent revision quality. ChatGPT help changed the structure of help-seeking, yet no corresponding essay-quality outcome was available. The evidence therefore supports a distinction between engaging with AI support and learning effectively from it.

Recognition and detection outcomes were reported in seven studies, covering faces, images, art, illustrations, and deepfake videos. In several cases, gaze became more focused as participants searched for anomalies, but this did not consistently predict successful recognition. Likewise, performance-related outcomes were common across the corpus, but formal statistical relationships between gaze and performance were reported in only a subset of studies.

No study in the final corpus formally assessed continuation intention as an outcome of the eye-tracking component, and no study included a dedicated, fully reported measure of user satisfaction linked to gaze. Two reports collected broader task-experience or evaluative information, but this was not equivalent to a validated satisfaction outcome. These absences represent clear gaps in the engagement literature.

Overall, the relationship between gaze and engagement was mostly correlational, descriptive, or inferred from parallel outcomes. Only a minority of studies formally tested whether gaze predicted liking, trust, comprehension, recognition, or task performance. The evidence therefore supports cautious statements about the behavioral expression of engagement, but not a simple conclusion that increased gaze toward AI-generated content represents greater user acceptance.

### 4.9. Moderators and Boundary Conditions

The heterogeneity of findings was partly explained by differences in modality and task. Static images, continuous text, subtitles, dynamic video, interactive feedback, and generated search responses impose different perceptual and cognitive demands. Authenticity-detection tasks encouraged anomaly search and concentrated fixation on potentially diagnostic details, whereas free-viewing tasks produced broader aesthetic or exploratory patterns. Reading tasks emphasized fixation duration, regressions, and saccades, while interactive systems generated transitions among multiple AOIs. Consequently, the direction of an “AI effect” often reflected the type of task and metric rather than content origin alone.

Expertise was one of the clearest participant-level boundary conditions. Radiologists approached synthetic medical images as domain experts, while most face and deepfake studies relied on non-experts. Participants with experience in digital imaging or AI generation tended to use more selective artifact-oriented strategies when judging AI images. AI proficiency was associated with better source identification in the Tibetan-illustration study, and language proficiency moderated the disruption associated with GPT-generated subtitles. In contrast, several studies collected expertise or familiarity information but did not incorporate it into statistical models. The evidence therefore suggests an expertise effect, but its form depends on whether expertise concerns the content domain, the task, or familiarity with generative technology.

Participant age was rarely tested as a formal moderator. Most samples were young adults or university students, limiting the ability to determine whether cognitive and attentional responses vary across the lifespan. Notable exceptions included the targeted sample of adults aged 40–65 in the health-advertising study and the broader age range in the AI-image-recognition study. Cha et al. [37] experimentally manipulated the depicted age of AI characters, but this concerned stimulus age rather than participant age. Age-related conclusions should therefore remain limited.

AI familiarity was also more often described than tested. Some participants had little previous experience with GenAI search or feedback, whereas others were frequent users of generative systems. The few studies that examined familiarity more directly suggested that it improved source recognition or encouraged more efficient artifact search. Nevertheless, familiarity could also increase trust or reduce critical monitoring, as suggested by the more direct help-seeking pathways observed with ChatGPT. More systematic interaction tests are needed to distinguish beneficial expertise from automation-related overreliance.

Disclosure and source belief were particularly influential. Creator labels changed gaze toward AI and human logos, while perceived authorship predicted viewing time for artworks more strongly than actual authorship. These studies demonstrate that actual provenance, disclosed provenance, and inferred provenance should not be treated as equivalent. A participant may visually process content according to what they believe its source to be, even when that belief is incorrect.

Realism and output quality constituted major boundary conditions in authenticity studies. Newer GAN faces were more difficult to distinguish from real faces than older synthetic outputs, and difficult deepfakes reduced classification accuracy more than easier manipulations. Generated subtitles that were fluent at the surface level nevertheless imposed greater visual and comprehension demands when translation choices, cultural references, or segmentation were suboptimal. AI cultural illustrations could be visually appealing while remaining semantically or culturally less faithful. Thus, surface realism and semantic quality represented distinct dimensions.

Complexity also moderated attention. Highly complex AI façade designs increased exploration but diverted attention from functional signage. Mind maps supported integration when their relational structure reduced extraneous processing, whereas interfaces requiring frequent movement among video, simulation, and search regions increased attentional switching. In design and translation tasks, complexity varied by workflow phase and brief: AI was more beneficial during divergent ideation or light post-editing than in later evaluative or coordination-intensive stages.

Semantic coherence and relevance were especially prominent in logos, subtitles, cultural illustrations, feedback, and educational summaries. High semantic relevance supported cognitive evaluation and sustained attention to logos, while feedback completeness and linguistic specificity attracted more processing than broad structural comments. Generated materials were more useful when they aligned closely with the source, learning goal, or cultural context. Where semantic fit was weak, greater gaze often reflected confusion or verification.

Finally, the quality of content matching placed important limits on causal interpretation. Several studies used strong matched comparisons, such as the same video with different subtitles, paired original and edited images, matched authentic and deepfake videos, or a common architectural base image. Others compared stimuli from different databases, production processes, or levels of visual complexity. Position confounding was present when AI and teacher feedback occupied fixed sides of the interface, while some face studies used only one stimulus for each condition cell. The MMAT assessment similarly identified residual stimulus, position, order, and repeated-measures confounding as recurrent concerns. Findings from poorly matched comparisons cannot be attributed solely to AI provenance.

### 4.10. Methodological Quality and Reporting Completeness

#### 4.10.1. Internal Validity

All 23 studies passed both MMAT screening questions: each stated a sufficiently clear research objective, and the collected data were relevant to addressing that objective. Sixteen reports were classified as quantitative non-randomized studies, five as randomized comparative trials, and two as mixed-methods studies. Methodological quality was evaluated at the criterion level; no summed MMAT score or minimum exclusion threshold was applied.

Among the 18 applicable quantitative non-randomized components, measurement appropriateness was generally strong: 16 were rated Yes and two Can’t tell. Exposure was implemented as intended in all 18. However, representativeness was a recurrent weakness: only three components received Yes, 13 received No, and two were rated Can’t tell. Small student samples, single-site recruitment, and narrowly specialized convenience samples were common. Complete outcome data were adequately reported in 13 components, while one received No and four received Can’t tell. Confounding was the other major weakness: only seven components adequately accounted for confounders, compared with 10 rated No and one Can’t tell. Frequent concerns included unmatched stimuli, fixed screen positions, non-counterbalanced condition order, differences in content difficulty or complexity, and analyses that did not fully account for repeated observations within participants and stimuli.

Among the five randomized studies, only one described randomization sufficiently to receive Yes; the remaining four stated that participants were randomly allocated but did not adequately explain sequence generation or allocation procedures. Baseline comparability was demonstrated in two studies and was unclear in three. Complete outcome data were available in four, whereas one subtitle study excluded approximately 23% of recruited participants following gaze-quality screening. Blinding was the weakest randomized-trial criterion: four studies received No, largely because participants could identify the search tool, feedback source, or visible intervention, and assessor blinding was not reported. Adherence to the assigned condition was satisfactory in all five studies.

Both mixed-methods studies provided an adequate rationale for combining quantitative and qualitative data. However, the strength of their qualitative components differed. The post-editing study integrated interview material with gaze, pause, and rating data and explicitly discussed discrepancies across measures, but the qualitative collection and coding procedures were not documented sufficiently. The Tibetan-illustration study provided an appropriate mixed-methods rationale but did not clearly report a formal integration strategy, transparent theme development, or participant quotations supporting its qualitative interpretations. Consequently, neither mixed-methods study met the criterion requiring all methodological components to achieve high quality.

#### 4.10.2. Eye-Tracking Reporting Completeness and Potential Validity Concerns

Eye-tracking reporting was uneven across the corpus. The device and model were fully reported in 18 studies, partially reported in three, and absent in two. Sampling rate was fully reported in 14 studies and partially reported in one, but was missing in eight. Calibration was adequately described in 11 studies, partially described in six, and absent in six. Validation or drift-checking procedures were less common, being fully reported in seven, partially in three, and absent in 13.

Only nine studies clearly identified the fixation- or saccade-detection algorithm, and only seven fully reported the numerical thresholds required to reproduce event classification. Twelve did not report an event algorithm, while 14 omitted numerical event thresholds. This is particularly important because fixation counts and durations are sensitive to the selected velocity, dispersion, and minimum-duration parameters.

AOI reporting was comparatively stronger: 16 studies provided clear AOI or ROI definitions, one was partially documented, and six relied on whole-stimulus measures, heatmaps, or qualitative feature descriptions. Preprocessing was fully described in 14 studies and partially described in six. In contrast, missing-data handling was fully reported in only seven studies, and explicit participant- or trial-exclusion rules were fully reproducible in only five. Ten studies provided no clear numerical exclusion criteria.

Pupil-processing procedures were applicable to only a small part of the corpus. Two studies fully described relevant preprocessing and two did so partially. Several reports interpreted pupil size as an indicator of cognitive demand or arousal without fully documenting baseline correction, blink interpolation, luminance control, or temporal alignment. Luminance or visual-property control was nevertheless reported adequately in 15 studies and partially in six.

Only five studies fully reported accessible data or code, with another 11 providing partial access or stating that materials were available on request. These findings indicate that reporting was strongest for the identification of hardware and principal outcomes, but considerably weaker for signal validation, event detection, missing data, and analytic reproducibility. Viewed specifically as potential threats to eye-tracking validity, the most recurrent concerns concerned calibration/validation, drift control, event detection, data-quality thresholds, AOI specification, and control of visual stimulus properties. Although calibration was adequately reported in 11 studies, explicit validation or drift procedures were fully described in only seven. Event-detection algorithms were identified in nine studies, and numerical thresholds were fully reproducible in seven, limiting confidence that nominally similar fixation outcomes were derived in comparable ways. Only five studies reported explicit numerical participant- or trial-level data-quality criteria. AOI definition was generally stronger, but six studies relied primarily on whole-stimulus or qualitative spatial analyses, and one provided only partial AOI documentation. Visual-property or luminance control was also inconsistent across comparisons. These limitations do not necessarily imply that the original studies were methodologically invalid; rather, incomplete reporting prevents several potential sources of measurement bias from being ruled out. The criterion-level eye-tracking heatmap in Supplementary Figure S1 summarizes these patterns without reducing them to a single quality score.

##### Sensitivity-Oriented Interpretation by Technical Reporting Completeness

Applying the predefined descriptive reporting criterion, eight studies provided higher technical reporting completeness across the core eye-tracking domains: Abu-Rayyash and Lacruz [29], Bozkir et al. [16], Yfantidou et al. [42], Yun and Kim [43], Yao et al. [37], Wong et al. [44], Zhou and Kawabata [4], and Ilyas et al. [28]. The remaining studies were retained in the synthesis but had more substantial omissions in one or more core areas such as sampling frequency, calibration or validation, event detection, numerical thresholds, or AOI specification. This classification reflects reporting completeness rather than an assumption that incompletely reported procedures were necessarily methodologically inadequate.

Importantly, several major conclusions of the review were also observed within the higher-reporting subgroup. Abu-Rayyash and Lacruz [29] showed that greater fixation and dwell on AI-generated subtitles coincided with poorer comprehension rather than a positive engagement advantage. Bozkir et al. [16] found no stable real-versus-synthetic gaze signature and showed that detection difficulty increased with synthetic-face realism. Zhou and Kawabata [4] demonstrated that perceived rather than actual authorship was more strongly associated with viewing time. Yao et al. [37] showed redistribution of visual effort during AI-assisted post-editing rather than a uniform reduction in overall effort. Yfantidou et al. [42] found reduced attention to task-relevant slogan information in AI-generated advertisements, while Wong et al. [44] observed only localized rather than global differences in scrutiny of synthetic medical images. These findings support the broader interpretation that gaze responses are task- and context-dependent rather than uniformly determined by AI provenance.

At the same time, several more specific claims in the broader corpus rely partly or primarily on studies with incomplete technical reporting. In particular, conclusions based on detailed scanpath organization, subtle AOI-transition patterns, or inferred processing differences were treated more cautiously where sampling, event-detection, calibration, or gaze-quality procedures were not sufficiently documented. The sensitivity-oriented comparison therefore did not overturn the principal synthesis, but it reduced confidence in fine-grained cross-study comparisons and reinforced the need to distinguish robust directional patterns from method-dependent interpretations. The resulting sensitivity-oriented grouping is summarized in Table 5.

#### 4.10.3. Reproducibility of AI-Generated Content

Reporting of AI-generation procedures showed a similarly uneven profile. The model or provider was adequately identified in 20 studies and partially identified in two; only one failed to report the generation system. Exact model version or snapshot was fully documented in 11 studies, partially documented in eight, and absent in four. This distinction is important because models bearing the same commercial name may change substantially across versions or deployment dates.

Prompt or template information was fully reported in seven studies and partially reported in eight. Four prompt-based studies did not report the prompt adequately, while prompt criteria were not applicable to several studies that used fixed public GAN or deepfake datasets. Generation settings were the least reproducible procedural element: only one study reported them sufficiently, 11 provided partial information, and eight omitted them. Generation date was fully reported in only one study and absent in 19 applicable studies.

Candidate-output counts and selection procedures were also inconsistently documented. Eleven studies clearly described selection or curation, including expert rating, filtering, or paired stimulus construction, while five provided partial information. Human editing or post-processing was fully documented in only three studies and partially in nine. In some cases, it was unclear whether the presented output was a raw model generation, a researcher-selected candidate, or an edited final stimulus.

Comparator matching was among the better-reported reproducibility dimensions: 15 studies provided a strong account of content or presentation matching, seven did so partially, and one provided clearly inadequate matching. However, even studies with visually standardized displays sometimes retained substantive differences in semantic content, realism, quality, or production process.

The generated stimuli or outputs were fully available in 10 studies and partially available in eight. Data or code availability was fully adequate in four studies and partial in 11. The resulting evidence base is therefore more reproducible with respect to which model was used than how the exact experimental outputs were generated, selected, modified, and presented.

The full item-level judgments are provided in Supplementary Tables S9–S11. In Supplementary Figures S1 and S2, green indicates sufficiently reported information, amber partial reporting, red absent or insufficient reporting, and grey not applicable. A study may use a technically rigorous eye-tracking protocol while providing insufficient information to reproduce its AI stimuli, or it may publish complete generation materials while underreporting gaze preprocessing. Keeping these domains separate provides a more precise assessment of the evidential strengths and limitations of the literature. The full item-level judgments are provided in Supplementary Tables S9–S11, with green indicating sufficiently reported information, amber partial reporting, red absent or insufficient reporting, and grey not applicable. A cross-domain synthesis of the comparative evidence, including the predominant patterns and principal boundary conditions identified across the review, is presented in Table 6.

## 5. Discussion

### 5.1. Summary of Key Findings and Contributions

This systematic review synthesized 23 comparative studies using eye tracking, gaze-based measures, or pupillometry to examine human responses to AI-generated content. The evidence covered direct provenance comparisons and task-embedded uses of generated feedback, summaries, search responses, and professional assistance. Across these heterogeneous designs, no modality-independent gaze pattern emerged. This lack of convergence should not be treated as an inherent property of AI-generated content, because the studies addressed different questions and varied in modality, task, comparator, interface structure, stimulus quality, and measurement strategy. Findings from direct artifact comparisons were therefore interpreted separately from findings concerning AI support within broader activities.

A recurring descriptive pattern, particularly in task-embedded studies, was redistribution of attention rather than a uniform increase or decrease. Participants shifted gaze among functional regions of learning, search, feedback, translation, and design interfaces [34,36,37,38]. These observations concern the organization of attention within specific tasks and should not be generalized as an intrinsic effect of AI provenance.

In some task-embedded studies, generated support reduced retrieval, search, or organization demands while participants also engaged in checking, comparing, or evaluating outputs [31,32,37,38]. Because these demands and measures varied substantially, this pattern is best treated as a candidate trade-off between access and evaluation rather than a common effect of AI-generated content.

A further contribution concerns the distinction between actual, disclosed, and perceived provenance. Objective origin did not always influence attention or evaluation, whereas disclosure labels and beliefs about authorship sometimes did [4,40]. Source beliefs should therefore be treated as a boundary condition rather than assumed to reflect objective provenance.

These conclusions must be interpreted in light of the evidence base. Most studies used small convenience samples, and representativeness, confounding, randomization reporting, technical eye-tracking reporting, and AI-stimulus reproducibility were uneven. The literature is therefore rapidly expanding but remains methodologically and conceptually fragmented.

### 5.2. Interpreting Visual Attention to AI-Generated Content

#### 5.2.1. Gaze Measures Are Not Direct Indicators of Preference, Quality, or Detection

A central implication of this review is that gaze measures cannot be interpreted independently of task context and accompanying outcomes. Fixations indicate that a location was selected for overt visual inspection, but they do not establish why it was selected or whether the information was understood, trusted, preferred, or successfully integrated. Longer fixation duration, greater dwell time, and repeated viewing may reflect interest or engagement, but they may also indicate uncertainty, processing difficulty, confusion, verification, or unsuccessful interpretation [10,13].

The subtitle study illustrates this ambiguity particularly clearly. GPT-4o-generated subtitles received more fixations, longer mean fixation durations, greater total viewing time, and a larger share of gaze than professionally translated subtitles. Considered alone, these indicators could be described as stronger visual engagement. However, the AI-generated subtitles were also associated with greater focal processing and poorer comprehension-related performance. Greater gaze therefore reflected additional processing burden and competition with the audiovisual narrative rather than a straightforward advantage in engagement or content quality [29].

The same distinction applies to authenticity detection. AI-edited images and deepfake videos sometimes generated concentrated gaze around suspected manipulation regions, yet this concentration did not consistently support successful classification. Participants often focused on faces, eyes, mouths, or suspected artifacts, but these strategies did not reliably differentiate authentic from synthetic material or distinguish correct from incorrect decisions [45,47,48]. A narrow or highly focal scanpath may therefore indicate systematic inspection without demonstrating that the inspected cues are diagnostically valid.

Conversely, the absence of a gaze difference does not establish psychological equivalence. AI-generated and teacher-generated feedback could attract similar fixation times while producing different verification strategies or patterns of attention across task components. Authentic and deepfake videos could elicit comparable fixation and saccade metrics while remaining difficult to classify accurately [33,34,48]. Equivalent eye-movement measures do not demonstrate equivalent trust, comprehension, memory, or decision quality.

The findings also caution against using gaze as a direct measure of preference. Actual AI or human authorship did not significantly alter major fixation measures in the art study, yet paintings perceived as human-made received longer viewing. Similarly, AI images sometimes attracted longer fixations because users were searching for generation artifacts rather than because they preferred the images [4,46]. Source beliefs may guide attention, while visual inspection may subsequently influence evaluation; the relationship is therefore reciprocal rather than unidirectional.

Pupil responses require similar caution. Pupil diameter and task-evoked dilation are influenced not only by processing effort, but also by luminance, arousal, fatigue, uncertainty, and emotional activation. Pupillometric differences should therefore be interpreted as convergent evidence rather than a pure measure of cognitive load, particularly when baseline correction, luminance control, blink treatment, or temporal alignment are underreported [11].

A defensible interpretive principle is therefore to treat gaze as context-dependent process evidence. Fixations, dwell, regressions, revisits, transitions, entropy, scanpaths, and pupil responses become psychologically meaningful only when considered alongside task instructions, stimulus properties, comparison conditions, participant characteristics, and behavioral or self-report outcomes. Throughout the present synthesis, terms such as effort, verification, integration, trust, and engagement are therefore used as psychological interpretations only when they are supported by task structure, behavioral performance, self-report, or additional physiological evidence; otherwise, findings are described at the level of observable gaze behavior.

#### 5.2.2. Modality- and Task-Dependent Patterns

In reading and language tasks, observed gaze differences varied with linguistic quality, readability, task purpose, and the need to coordinate generated text with other information. Direct text-origin comparisons and task-embedded assistance were therefore interpreted separately. AI-generated subtitles were associated with greater visual demand and poorer comprehension than professional subtitles, whereas post-editing assistance reduced source-text consultation but introduced additional editing and evaluative activity [28,29,37].

In static visual and authenticity tasks, participants often concentrated on suspected anomalies in generated faces, manipulated images, synthetic radiographs, artworks, or deepfake videos. However, focused inspection did not consistently support accurate classification, particularly as synthetic realism increased [16,47]. These findings concern task-specific inspection strategies rather than a general gaze response to AI-generated imagery.

Educational and interactive studies primarily showed differences in how gaze was distributed across summaries, videos, search areas, simulations, feedback panels, and task representations [32,34,36]. These patterns describe cross-region allocation and switching; successful information integration was inferred only when corresponding learning or performance outcomes were available.

Professional and creative studies showed phase-specific differences rather than a generalized benefit of AI assistance. AI support was sometimes associated with reduced retrieval or search demands during ideation, while evaluation, refinement, and coordination introduced additional task activity in later phases [37,38].

Task instructions shaped gaze independently of modality. Free viewing, aesthetic evaluation, authenticity detection, learning, revision, and professional decision-making activate different attentional goals. Accordingly, direct provenance comparisons and task-embedded AI studies should remain analytically distinct and should not be combined into a generalized “AI effect” on gaze.

### 5.3. Methodological Challenges in Eye-Tracking Research on AI-Generated Content

The first major methodological challenge concerns sampling and statistical power. Most studies used small convenience samples, often comprising university students or young adults. Only a limited number examined domain experts, children, older adults, or participants recruited from a clearly defined target population. This constrains generalizability because trust, detection, reading, and engagement may vary with age, culture, language proficiency, professional expertise, and prior exposure to AI.

Stimulus confounding represented a second major problem. AI and comparator content were not always matched for semantic content, length, readability, visual complexity, luminance, emotional valence, realism, or production quality. Some studies drew AI and real images from different source pools, while others used fixed screen positions or only one stimulus for each experimental condition [33,39,46]. Under such conditions, differences attributed to AI provenance may partly reflect stimulus quality, complexity, position, or database origin.

AI-stimulus construction involves a difficult trade-off between control and ecological validity. Selecting only the most realistic or successful model outputs improves experimental comparability but underrepresents the variable-quality content users encounter in practice. Presenting unfiltered outputs increases ecological validity but introduces uncontrolled heterogeneity. Studies should therefore state clearly whether outputs were raw, selected, curated, edited, or regenerated.

Repeated-measures structure was not always addressed appropriately. Many studies contained multiple observations per participant and multiple responses per stimulus, yet some analyses did not clearly model both participant- and item-level dependence. Mixed-effects models with participant and stimulus random effects are preferable when observations are nested or crossed, as demonstrated in some of the more rigorous studies [4].

Randomized studies also showed reporting weaknesses. In most cases, authors stated that participants had been randomly assigned but did not describe sequence generation or allocation procedures. Baseline comparability was frequently unclear, and blinding was uncommon. Although participant blinding may be impossible when AI interfaces or feedback sources are visible, outcome coding, data cleaning, and statistical analysis can sometimes still be conducted without knowledge of condition.

Eye-tracking methodological reporting was inconsistent, and several omissions correspond to recognized threats to the validity of eye-movement research. Sampling rate, calibration accuracy, validation and drift correction, fixation-detection algorithms, event thresholds, missing-data handling, and participant-exclusion criteria were absent or incomplete in a substantial proportion of studies. Calibration and drift are particularly important because spatial measurement error can shift recorded gaze relative to the intended stimulus region, potentially altering AOI-based estimates. Event-detection choices can likewise produce different fixation counts and durations from the same underlying gaze stream. In pupillometric studies, insufficient control of luminance, baseline definition, blink handling, and temporal windows can complicate interpretation of pupil changes. Finally, AOI construction introduces an additional source of analytic flexibility: poorly justified boundaries, differences in AOI size, post hoc region selection, and inconsistent treatment of dynamic AOIs can alter dwell, fixation, revisit, and transition estimates. These factors should therefore be considered alongside general study-design quality when evaluating evidence from eye-tracking experiments [13,49].

Heatmaps were widely used, but they should be interpreted as descriptive visualizations rather than inferential evidence. A heatmap does not represent the temporal sequence of viewing and cannot, by itself, demonstrate a statistically meaningful difference. Similarly, qualitative descriptions of “more focused” or “broader” gaze should ideally be supported by formal entropy, scanpath, transition, or spatial-distribution metrics.

AOI construction was another source of heterogeneity. Some studies prespecified meaningful regions such as subtitles, slogans, faces, feedback panels, and summary areas. Others relied on whole-stimulus metrics or post hoc visual interpretation. Dynamic video and mobile-eye-tracking studies additionally require explicit reporting of how AOIs were tracked, resized, corrected, and handled when they overlapped.

Pupillometry was especially vulnerable to overinterpretation. Not all studies documented luminance control, baseline correction, blink interpolation, or temporal windows, despite interpreting pupil changes as evidence of processing effort. Pupil data should therefore be triangulated with performance, self-report, and other physiological measures rather than treated as a standalone cognitive-load indicator [11].

Consequently, lack of methodological reporting was not treated merely as a reporting-quality issue; it directly informed the confidence placed in cross-study interpretations, particularly when conclusions depended on small differences in fixation duration, AOI transitions, scanpath organization, or pupil response.

Finally, the reproducibility of AI-generated stimuli was frequently insufficient. Although most papers named the provider or model family, exact model versions, prompts, generation settings, generation dates, candidate-output counts, selection criteria, and human editing were inconsistently reported. A label such as “GPT-4,” “ChatGPT,” or “Midjourney” does not define a stable stimulus-generation procedure, particularly when commercial systems change over time.

Interactive outputs introduce an additional reproducibility problem because they depend on prior conversation, retrieval context, system instructions, and user behavior. Reproducing an initial prompt is not sufficient if the participant saw a response shaped by a longer interaction history. Studies should preserve the complete input–output sequence that produced the analyzed exposure.

### 5.4. Gaps in the Literature and Open Questions

The first major gap concerns initial attentional capture. Only a small number of studies used TTFF or first-fixation measures. The field can therefore make stronger claims about sustained attention and information allocation than about whether AI content attracts attention rapidly or automatically.

Trust and evaluative outcomes were also underrepresented. Trust, credibility, and authenticity were frequently discussed but rarely measured as distinct constructs. Only one study in the final corpus included a direct trustworthiness outcome, while several others addressed related source beliefs indirectly [4,39,40]. Continuation intention was not formally measured in any of the final eye-tracking components, and dedicated satisfaction measures were effectively absent.

Formal links between gaze and functional outcomes were uncommon. Many papers reported gaze and performance in parallel without testing whether eye movements predicted comprehension, detection accuracy, trust, decision quality, or learning. As a result, claims that a gaze pattern represents successful processing often remain inferential.

Dynamic and socially interactive contexts are underdeveloped. Most studies examined static content or tightly controlled interfaces. Few investigated long-term use, repeated exposure, collaborative settings, or evolving conversational relationships with AI. No final included study analyzed interpersonal or intersubject gaze alignment, despite the increasing use of AI agents in collaborative and educational interaction.

The population base remains narrow. Student and young-adult samples dominated, while evidence from children, older adults, low-literacy users, diverse linguistic populations, and users with disabilities was limited. Expert samples were also rare. It remains unclear whether expertise produces genuinely more diagnostic visual strategies or only greater confidence in existing strategies.

Few studies independently manipulated actual origin, disclosed origin, and perceived origin. The available logo and art findings suggest that these dimensions can exert different effects [4,40]. Factorial research is needed to determine when gaze responds to perceptual content and when it responds to expectations about source.

The field also lacks standardized operational definitions. Fixation duration, dwell, engagement, cognitive load, and verification were measured and interpreted in different ways. Custom metrics may capture task-specific processes, but they limit cross-study comparison and currently make a pooled meta-analysis difficult.

Finally, the temporal stability of findings is uncertain. Generative systems evolve rapidly, and results based on one model version may not generalize to later versions with different fluency, realism, or interface behavior. Replication across model generations should therefore become a central research priority.

### 5.5. Recommendations for Future Research and Practice

#### 5.5.1. Study Design and Stimulus Construction

Future studies should use adequately powered and, where feasible, preregistered designs. Primary gaze outcomes, behavioral outcomes, moderators, data-quality thresholds, and exclusion criteria should be defined in advance.

Multiple stimuli should represent each condition. Studies should avoid treating one face, image, text, or response as representative of an entire AI or human category. Analyses should model participant- and stimulus-level variability using appropriate multilevel methods.

AI and comparator stimuli should be matched on modality-specific properties, including length, readability, topic, semantic content, grammatical quality, resolution, luminance, contrast, complexity, realism, valence, and cultural relevance. Matching should be documented empirically rather than asserted.

Raw, selected, curated, human-edited, and hybrid outputs should be treated as distinct forms of exposure. Researchers should report the number of generated candidates, selection criteria, excluded outputs, and any post-processing.

Studies examining source effects should distinguish actual provenance from source disclosure and perceived provenance. Factorial designs can separate the effect of the artifact from the effect of labeling.

Interactive comparisons should isolate the function of AI where possible. AI versus no-tool comparisons conflate generated content, additional information, interface design, personalization, and response timing. Matched human feedback or retrieved non-generative support may provide more interpretable comparators.

#### 5.5.2. Reporting of AI Systems and Experimental Materials

Reports should state the provider, exact model name and version, access route, generation date, system prompt, user prompt, generation settings, seed where applicable, and the full output presented to participants.

For API-based systems, relevant parameters such as temperature, top-p, maximum tokens, and retrieval settings should be reported. Interactive studies should preserve the full conversation and retrieval history, not only the initial prompt.

Researchers should document the number of candidate outputs, regeneration attempts, selection procedures, human editing, cropping, color adjustments, grammar correction, and other transformations.

Stimuli, prompts, output-selection records, and generation code should be deposited in a stable repository whenever legally and ethically possible. For proprietary systems, a time-stamped archive is especially important.

#### 5.5.3. Eye-Tracking Measurement and Analysis

Eye-tracking reports should identify the device, sampling frequency, viewing distance, calibration and validation procedure, accepted accuracy, drift-correction rules, event-detection algorithm, numerical thresholds, interpolation, smoothing, blink treatment, missing-data procedures, and participant/trial exclusions. Future methodological work should also develop and validate a dedicated eye-tracking risk-of-bias instrument that distinguishes incomplete reporting from demonstrable threats to internal validity and that explicitly addresses calibration accuracy, drift, visual-environment control, AOI construction, event detection, data loss, and preprocessing flexibility.

AOIs should be defined prospectively and accompanied by diagrams or machine-readable coordinates. Dynamic AOIs should include information on tracking, resizing, overlap, and frame-level correction.

A limited core set of theoretically justified measures is preferable to a large set of weakly motivated variables. Depending on the task, this might include one orienting measure, one sustained-attention measure, one reinspection or transition measure, and one functional outcome.

Heatmaps should remain descriptive supplements. Repeated observations should be analyzed using hierarchical or mixed-effects models, with participant and item variance represented explicitly.

Pupillometry studies should report baseline windows, luminance control, blink interpolation, smoothing, normalization, and temporal windows. Pupil findings should be interpreted in combination with performance, self-report, or other physiological evidence.

#### 5.5.4. Linking Gaze to Functional Outcomes

Future research should test whether gaze differences predict outcomes that matter: comprehension, calibrated trust, detection, memory, learning transfer, decision quality, revision quality, or behavioral choice.

Multilevel regression, mediation, moderation, and out-of-sample prediction could determine whether attention to a summary predicts retention, whether artifact-focused gaze predicts correct detection, or whether repeated checking of an AI response predicts calibrated reliance.

Engagement should be measured multidimensionally. Visual, behavioral, evaluative, and learning engagement should not be collapsed into one construct. Greater dwell should not be labeled positive engagement unless it converges with preference, persistence, satisfaction, or successful performance [24].

Longitudinal designs are needed to assess whether initial verification costs diminish with experience, whether reduced effort leads to skill loss, and whether repeated exposure recalibrates trust.

#### 5.5.5. Cumulative and Open Research Practices

The field would benefit from benchmark datasets containing carefully matched AI and human content across text, image, and video modalities. These resources should include several model generations, validated difficulty levels, provenance information, normative ratings, and low-level stimulus properties.

Preregistered multisite replications would improve power and cultural generalizability. Common tasks could be repeated across languages, age groups, expertise levels, and model versions.

A reporting checklist integrating eye-tracking standards with AI-specific information should be developed. Core AI items should include model version, prompt, date, settings, candidate count, selection, editing, and stimulus availability. Progress toward cumulative and interoperable research infrastructures may also require collaboration across universities, technology providers, professional organizations, and data repositories. This need parallels broader evidence that the introduction of generative AI is supported by inter-organizational networks and ecosystem-level collaboration rather than isolated organizational adoption [50]. 

Future systematic reviews in this rapidly developing area should broaden database coverage by searching specialized computing and human–computer interaction sources, particularly ACM Digital Library and IEEE Xplore, in addition to multidisciplinary citation databases such as Scopus and Web of Science. This is especially important for newly published conference research, for which indexing in multidisciplinary databases may lag behind publication in the publisher’s digital library. Updated searches close to manuscript submission, together with backward and forward citation searching and systematic attempts to retrieve initially unavailable reports, would reduce the risk of database-coverage and availability bias and provide a more comprehensive representation of this rapidly evolving evidence base.

Open materials should include de-identified gaze data, event-level data, AOI definitions, stimuli where permissible, exclusion logs, and analysis code. These practices would improve independent verification and may enable future meta-analysis once sufficiently comparable studies accumulate and compatible effect estimates can be derived.

### 5.6. Implications for Theory and Practice

#### 5.6.1. Theoretical Implications

The interpretations in this section are theory-generating and extend beyond the direct descriptive findings. The reviewed evidence suggests an organizational framework rather than a confirmed causal model. Stimulus properties, source information, and task goals may be associated with differences in orienting and information selection. Observable gaze processes can then be considered alongside candidate interpretations involving fluency, uncertainty, verification-related demand, cognitive effort, and information integration. These interpretations remain analytically separate from functional outcomes such as comprehension, evaluation, detection, and behavior.

AI provenance is not a directly visible perceptual feature but is inferred from content cues, source labels, prior beliefs, and contextual expectations. Actual, disclosed, and perceived provenance may therefore be associated with different patterns of attention and evaluation.

The review also suggests the concept of an AI-related verification burden as a candidate interpretive framework. In some task contexts, AI support was associated with reduced search, retrieval, or production demands alongside increased checking, comparison, or evaluative activity. These patterns are consistent with a possible shift toward verification-related demands, but they should not be interpreted as evidence of a universal cognitive mechanism. Gaze behavior alone cannot establish verification burden, and this interpretation is strongest where eye-tracking findings converge with task performance, behavioral indicators, self-report, or complementary physiological measures.

Cognitive load theory provides one useful, but non-deterministic, framework for interpreting these patterns. Intrinsic load is expected to vary primarily with the inherent complexity of the task and material and may be accompanied by longer fixation, increased rereading, or greater pupil response when more elements must be processed simultaneously. Extraneous load may arise from avoidable presentation or interface demands, such as poorly segmented AI-generated subtitles, competing interface regions, visually complex generated designs, or repeated switching between generated output and source information; relevant gaze manifestations may include increased dwell, regressions, cross-AOI transitions, or dispersed search. Germane processing, where considered in the learning literature, may involve sustained inspection and coordinated transitions that support integration of task-relevant information. Importantly, these mappings are not one-to-one: none of these eye-tracking indicators can independently identify a specific type of cognitive load. Cognitive-load interpretations require convergence with task structure, performance, subjective load measures, or complementary physiological evidence.

Processing fluency should likewise be distinguished from accuracy and reliability. Highly fluent or realistic AI content may be easy to process while remaining difficult to evaluate critically. Conversely, less fluent output may increase gaze and effort but make limitations easier to recognize [20,21].

**Figure 2:**
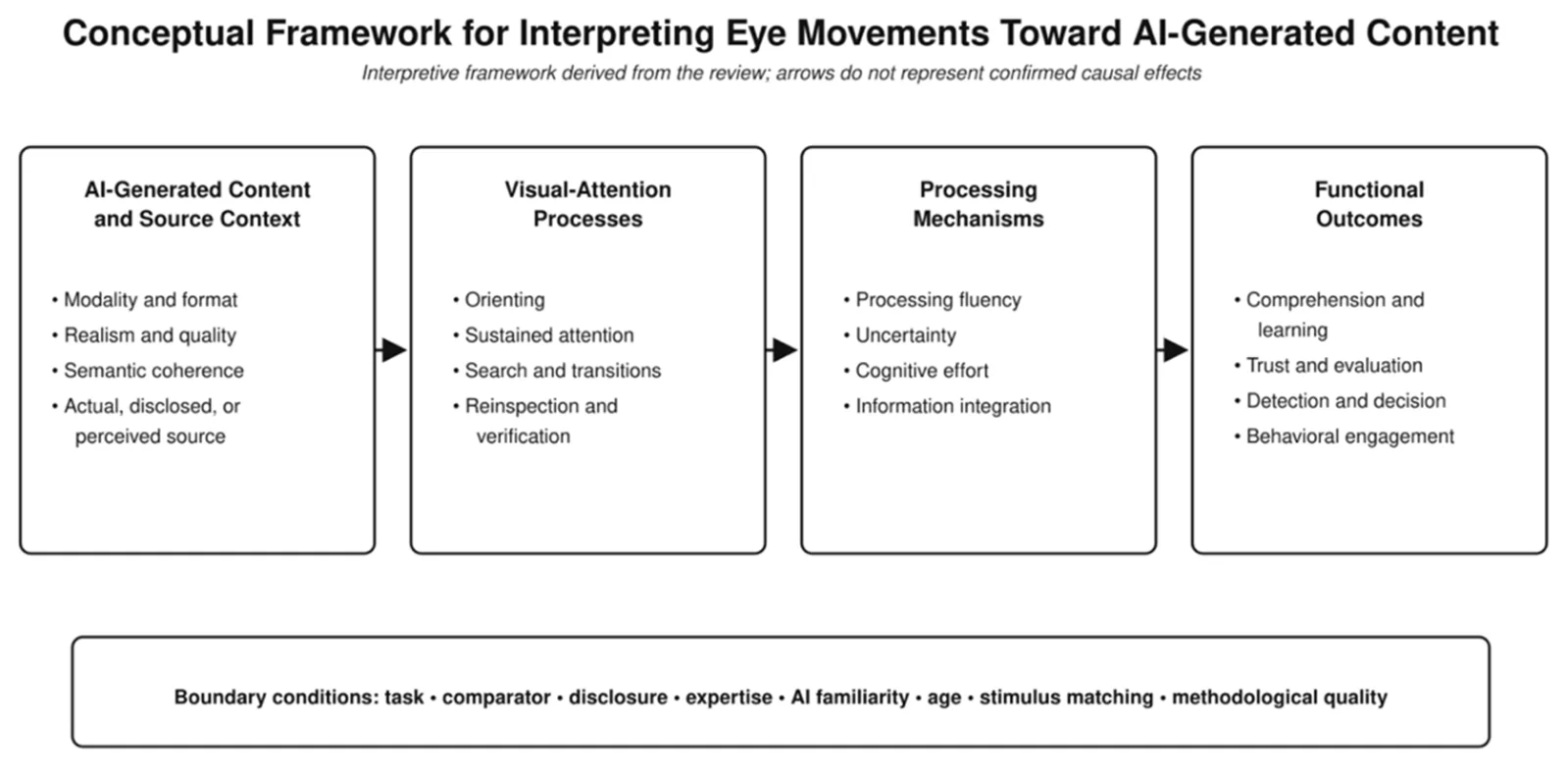
Evidence-informed framework separating exposure and boundary conditions, observable eye-movement processes, candidate processing interpretations, and functional outcomes. Arrows indicate theoretically plausible relationships suggested by the review and do not represent established causal pathways.

Finally, engagement should be conceptualized as multidimensional. Attention may be sustained because content is useful or appealing, but also because it is confusing, suspicious, or difficult. Human–AI engagement theories should distinguish productive involvement from verification, repair, and cognitive friction. The resulting evidence-informed framework is presented in Figure 2.

##### Testable Propositions Derived from the Review

The framework generates several propositions that can be tested prospectively in future eye-tracking experiments. These propositions are intended as theory-generating implications of the review rather than hypotheses tested by the present systematic review.

P1—Verification-burden proposition. When AI-generated content is perceived as uncertain, potentially inaccurate, or difficult to authenticate, users will show greater reinspection and verification behavior—such as longer dwell, more revisits or regressions, and more transitions to comparison information—than when equivalent content is perceived as reliable, with the magnitude of this effect moderated by task demands and expertise.

P2—Extraneous-load proposition. When AI-generated information introduces avoidable presentation or coordination demands, such as poor segmentation, excessive interface switching, or irrelevant visual complexity, gaze will become more fragmented or repeatedly redirected across information sources, and these patterns will be associated with higher independently measured extraneous cognitive load and poorer task performance.

P3—Structured-integration proposition. When AI-generated support organizes task-relevant information into a coherent structure, coordinated gaze allocation across complementary information regions will be associated with improved comprehension or retention relative to unstructured or absent AI support, provided that output quality is sufficiently high.

P4—Provenance-belief proposition. Perceived or disclosed provenance will moderate gaze allocation independently of actual provenance: identical or closely matched content will be inspected differently when users believe it to be AI-generated rather than human-generated, particularly in tasks requiring credibility, authenticity, or quality evaluation.

#### 5.6.2. Practical Implications

In education, generated summaries and structured visual representations can support comprehension and retention, particularly when they make conceptual relations explicit [31,32]. GenAI search and feedback should nevertheless be designed to encourage source checking, explanation, and transfer rather than only rapid answer access [34,36].

In translation and media accessibility, automated subtitles and suggestions should be professionally reviewed for segmentation, timing, cultural adequacy, and narrative coherence. Increased visual attention to automated output may indicate additional processing burden rather than improved accessibility [29,37].

In design and advertising, novelty should not be equated with communication effectiveness. Visually complex generated designs may attract attention while reducing visibility of slogans or signage. Semantic relevance, cultural fidelity, and visual hierarchy remain essential [40,41,42,43]. In organizational and corporate communication, these considerations extend beyond visual attention to the credibility, transparency, stakeholder interpretation, and strategic coherence of AI-mediated messages, particularly when organizations use AI to communicate socially sensitive or value-laden information [51].

For misinformation and deepfake detection, simply instructing users to inspect the eyes, mouth, or face is unlikely to be sufficient. Participants already attended to these regions, yet gaze did not consistently predict successful classification. Training should emphasize validated diagnostic cues, contextual inconsistencies, source verification, and uncertainty management [47,48].

In healthcare, synthetic content should be evaluated with expert participants and clinically meaningful outcomes. Gaze can reveal changes in scrutiny, but diagnostic accuracy and patient-relevant consequences must remain primary [44].

Adaptive systems should not treat gaze as an unambiguous indicator of interest, confusion, trust, or disengagement. Gaze-based personalization should combine eye movements with performance, interaction history, explicit feedback, and uncertainty estimates.

### 5.7. Strengths and Limitations of the Review

A principal strength of this review is its strict comparative scope. Included studies were required to compare AI-generated content with human-generated, authentic, professional, traditional, or no-AI conditions. This improved interpretability and prevented the review from becoming a general inventory of AI systems using eye tracking.

The review also integrated multiple content modalities while preserving the distinction between direct artifact comparisons and interactive AI-support studies. Study-level extraction covered participants, models, content, comparators, tasks, devices, gaze measures, cognitive and behavioral outcomes, moderators, and generation procedures.

The review retained three analytically separate domains: general study-design quality assessed with MMAT, eye-tracking technical reporting completeness and potential validity concerns, and AI-stimulus reporting and reproducibility. These domains were not collapsed into a single score. The sensitivity threshold distinguishing higher from more limited technical reporting completeness was descriptive and review-specific; it was not a validated risk-of-bias cutoff, and missing reporting was not treated as evidence that a procedure had not been performed.

The review retained null findings and distinguished actual, disclosed, and perceived provenance. Missing information was coded as unreported rather than inferred.

Several limitations should nevertheless be considered. The strict comparator criterion excluded potentially informative non-comparative studies. The conclusions therefore apply specifically to comparative evidence.

Substantial heterogeneity in modalities, tasks, comparators, metrics, and analyses precluded a meaningful pooled meta-analysis. The synthesis consequently emphasizes patterns and boundary conditions rather than an overall effect size. This heterogeneity also limits the level at which substantive conclusions can be drawn. In particular, the absence of a common gaze pattern across studies cannot be interpreted as evidence for a stable characteristic of AI-generated content, because differences among studies may arise from distinct tasks, interfaces, comparator structures, stimulus properties, and operational definitions. Conclusions were therefore restricted to evidence classes and boundary conditions rather than generalized to AI-generated content as a unitary category. This heterogeneity also prevented defensible quantitative pooling within the four synthesis subgroups and required conclusions to remain specific to evidence classes and boundary conditions rather than to AI-generated content as a unitary category.

The search was restricted to English-language peer-reviewed journal articles and full conference papers published from 2015 onward. Scopus, Web of Science Core Collection, and PubMed were searched, whereas the ACM Digital Library and IEEE Xplore were not searched as separate databases. However, Scopus indexes substantial journal and conference content published by ACM and IEEE, resulting in considerable overlap with these specialized digital libraries. Recently published papers may still be affected by indexing delays of up to approximately 12 weeks. Consequently, some very recent studies, particularly conference papers, may have been missed. The language and publication-type restrictions may also have excluded relevant non-English studies, dissertations, technical reports, and recent preprints.

A further limitation concerns report availability. Of the 100 reports sought for full-text retrieval, 27 could not be obtained and therefore could not undergo eligibility assessment. These records were not excluded on substantive eligibility grounds; rather, their eligibility could not be determined because the full reports were unavailable. This creates a potential source of availability bias and means that the conclusions represent the retrievable evidence identified through the databases searched rather than an exhaustive census of all potentially relevant studies. In addition, terminology in this area is unstable, and potentially relevant studies may use terms such as synthetic media, automated writing, computer-generated imagery, or virtual characters rather than generative AI.

The rapid development of AI systems also limits temporal generalizability. Results obtained with one model snapshot or interface may not transfer to later versions. Finally, MMAT assesses general study-design quality but does not directly evaluate eye-tracking-specific procedures. The purpose-built eye-tracking and AI-stimulus matrices increased transparency regarding technical reporting completeness, potential validity concerns, and reproducibility, but they are not validated risk-of-bias instruments. An unreported procedure cannot be assumed to have been omitted. The assessments should therefore be interpreted as structured indicators of transparency and potential concerns rather than definitive bias classifications.

## 6. Conclusions

Future research should combine well-powered comparative designs, carefully matched stimuli, transparent generation records, standardized eye-tracking reporting, and multilevel analyses linking gaze to comprehension, trust, detection, decision quality, and behavior. Eye tracking offers a sensitive method for examining how people encounter AI-generated information, but its value does not lie in treating gaze as a direct readout of the mind. Its strongest contribution is as one component of a theoretically grounded account of how users select, inspect, verify, understand, evaluate, and act on increasingly synthetic information.

Across 23 comparative studies, the available evidence did not support a modality-independent gaze signature of AI-generated content. Observed differences varied with modality, task, comparator, interface structure, output quality, source belief, participant expertise, and measurement choices. Direct provenance comparisons and studies of task-embedded AI support addressed different questions and were therefore interpreted separately. Longer or more concentrated viewing was not treated as evidence of preference, engagement, comprehension, trust, or cognitive load unless supported by task characteristics or complementary behavioral, subjective, performance-based, or physiological outcomes.

The principal contribution of this review is therefore contextual and methodological rather than the identification of a universal AI effect. Eye tracking can reveal how visual attention is allocated and reorganized, but it cannot independently establish higher-level psychological states. Confidence in cross-study interpretation is further limited by small samples, heterogeneous gaze measures, incomplete technical reporting, and limited documentation of AI-stimulus generation. Future studies should use carefully matched stimuli, transparent generation records, standardized eye-tracking procedures, and functional outcomes that connect gaze with comprehension, detection, decision quality, or behavior.

## Figures and Tables

**Table 1:** Overview of the included evidence base.

Dimension	Summary	Interpretive Note
Publication period	2020–2026	18/23 studies published in 2025–2026
Publication type	17 journal articles; 6 full conference papers	Journal articles predominated
Final eye-tracking sample	778 participants	Range 10–82; median 30; mean 33.8
Primary modality	Static visual 10; textual/linguistic 7; multimodal/interactive 4; audiovisual/deepfake 2	Primary categories were mutually exclusive
Study design	Within-subject 16; between-subject 5; hybrid 2	Repeated-measures designs predominated
Participant group	Students/learners 11; general/mixed adults 9; design/cultural specialists 2; experts 1	Student samples were common
Primary domain	Visual/synthetic media 7; education 6; design/communication 5; language/translation 4; medical imaging 1	Interdisciplinary evidence base

**Table 2:** Key methodological and analytical characteristics of the 23 included studies.

Study	N	AI-Generated Exposure/Model	Comparator	Eye Tracker	Sampling Rate	Core Gaze Indicators
Zhou & Kawabata [4]	34	Disco Diffusion paintings	Human-made paintings	Tobii Pro Spectrum	300 Hz	Fixation count, mean/total fixation duration
Bozkir et al. [16]	22	PGGAN/StyleGAN/StyleGAN2 faces	Real faces	Tobii Pro Spectrum	600 Hz	Fixation rate, saccade length
Ilyas et al. [28]	12	Microsoft Bing Copilot literary passages; version/model NR	Human-authored passages	Tobii 4C	90 Hz	Fixation duration/rate, saccade amplitude, pupil, regressions
Abu-Rayyash & Lacruz [29]	82	GPT-4o Arabic subtitles	Professional subtitles	RealEye	30 Hz	Fixation count/duration, dwell, TTFF, K-coefficient
Komninos et al. [30]	13	Llama 3 8B conversational context	Baseline transcription	Tobii T120	NR	Fixation count, total fixation duration
Morita et al. [31]	24	Text/image summaries; ChatGPT + image-generation models	No GenAI augmentation	Tobii Pro, model NR	90 Hz	Fixation duration, AOI ratio, scanpaths
Yuan et al. [32]	80	Doubao text/mind-map summaries	Video without generated summary	Model NR	1000 Hz	Total fixation time, fixation-time proportion, scan patterns
Cao et al. [33]	10	DeepSeek-R1 feedback	Teacher feedback	EyeLink 1000 Plus	NR	Per-word fixation duration/count, backtracking
Cosentino et al. [34]	30	GPT-4 personalized feedback	Human-instructor feedback	Tobii mobile glasses	50 Hz gaze *	AOI time, transitions, pupil-derived load
Chen et al. [35]	38	ChatGPT 4.0 help responses	Human-expert help	Tobii Pro Nano	NR	ROI gaze stays, process transitions
Xu et al. [36]	57	GLM-4/Qwen2 RAG-generated answers	Traditional web search	EyeLink 1000 Plus	NR	Dwell, fixation count, cross-AOI saccades/transitions
Yao et al. [37]	26	GPT-4 post-editing suggestions	Traditional post-editing	EyeLink 1000 Plus	1000 Hz	Source/target fixation duration and count per word
Fu et al. [38]	18	GPT-4.1 responses; Midjourney visuals	Human designer/conventional images/no stimulus	Tobii Pro Fusion	120 Hz	Fixation count/duration, pupil measures, gaze trajectory
Cha et al. [39]	24	Midjourney v6.1 2D/3D characters	Real facial photographs	Tobii Pro Spectrum	1200 Hz	Fixation count/duration, pupil diameter
Guan et al. [40]	54	ChatGPT-4o/Midjourney v5.2 logos	Human-created logos	Tobii Pro Nano	60 Hz	Fixation count/duration, TTFF
Liu et al. [41]	38	ERNIE/WenxinYiyan illustrations	Human-drawn illustrations	Tobii Pro, model NR	NR	Gaze duration, scanning range, heatmaps
Yfantidou et al. [42]	42	AI-generated advertising images; model NR	Hand-drawn and stock images	RealEyes	NR	TTFF, dwell, fixation count, revisits
Yun & Kim [43]	30	SDXL + ControlNet façade designs	Real storefront photograph	iMotions WebET	30.19 Hz	Dwell, fixation count/duration, revisits
Wong et al. [44]	16	RoentGen synthetic chest X-rays	Real chest X-rays	EyeLink 1000 Plus	500 Hz	Fixation profiles, saccades, saliency metrics
Cartella et al. [45]	20	Stable Diffusion/InstructPix2Pix edited images	Original images	Model NR	NR	Fixations, scanpaths, saliency entropy
Osińska et al. [46]	36	GPT-4o images/synthetic faces	Authentic photographs	GazePoint GP3 HD	150 Hz	Fixation count/duration, saccades, revisits, gaze paths
Gupta et al. [47]	40	Deepfake videos	Real videos	Tobii TX300	300 Hz	Fixation count/duration, re-fixations, entropy, scanpath
Holmes et al. [48]	32	DFDC deepfake videos	Authentic videos	Pupil Invisible	NR	Fixation duration/count, saccade count, dynamic AOIs

* Cosentino et al. separately reported pupil acquisition at 250 Hz; this discrepancy is retained as reported by the original study.

**Table 3:** Summary of eye-tracking methods and reporting.

Feature	Corpus Summary	Implication
Device family	Tobii (12); EyeLink (4); webcam platforms (3); mobile/head-mounted glasses (2); other/unspecified (2)	Hardware and remote methods varied substantially
Sampling rate	Reported in 15 studies; 30–1200 Hz; median 150 Hz	Eight studies did not report a usable rate
Calibration/validation	Calibration adequately reported in 11; validation/drift fully in 7	Validation was less consistent
Event detection	Algorithm identified in 9; numerical thresholds fully in 7	Limits reproducibility
AOIs	Formal AOIs/ROIs in 16 studies	Seven used whole-stimulus/qualitative region analysis
Data quality	Explicit numerical criteria in 5; partial/qualitative in 8	Ten lacked reproducible thresholds
Common measures	Fixation count (14), total fixation/dwell (14), mean fixation duration (12)	Orienting, transition, entropy, and pupil measures were less common

**Table 4:** Descriptive quantitative comparison of eye-tracking metric families across synthesis subgroups.

Synthesis Subgroup	Studies (*n*)	Fixation Count	Mean Fixation Duration	Total Fixation/Dwell	Reinspection/Regression	Scanpath/Saccade/Transition/Entropy	Pupil Measures
Reading and language processing	3	2/3	2/3	2/3	1/3	2/3	1/3
Authenticity and detection	9	5/9	6/9	3/9	2/9	7/9	1/9
Learning and information integration	6	2/6	1/6	5/6 *	1/6	5/6 ^†^	1/6
Professional and creative tasks	5	5/5	3/5	4/5	2/5	1/5	1/5

* Chen et al. reported ROI gaze stays/process-level evidence rather than a conventional fixation-duration estimate and is not counted as a formal total-fixation measure. ^†^ Includes formal and clearly reported process-level scanpath/transition analyses; Chen et al. and Yuan et al. contributed qualitative/process-based gaze-organization evidence.

**Table 5:** Sensitivity-oriented grouping according to eye-tracking technical reporting completeness.

Reporting Group	Studies	Interpretation
Higher technical reporting completeness	Abu-Rayyash & Lacruz [29]; Bozkir et al. [16]; Yfantidou et al. [42]; Yun & Kim [43]; Yao et al. [37]; Wong et al. [44]; Zhou & Kawabata [4]; Ilyas et al. [28]	≥5 of 7 core eye-tracking technical domains fully reported; greater confidence in interpreting gaze-based findings
More limited technical reporting completeness	Remaining 15 studies	One or more substantial reporting gaps; findings retained but fine-grained gaze interpretations treated more cautiously

Note. Core domains were device/model, sampling rate, calibration, validation/drift control, event-detection algorithm, numerical event thresholds, and AOI/ROI definition. This grouping is a descriptive sensitivity device and not a validated quality score or risk-of-bias classification. Full criterion-level judgments are reported in Supplementary Table S10A.

**Table 6:** Cross-domain synthesis of comparative evidence.

Evidence Domain	Representative Content/Tasks	Predominant Pattern	Boundary Conditions
Reading and language	AI text, subtitles, feedback, post-editing	AI altered reading strategy; subtitles showed the clearest processing burden	Text quality, proficiency, task brief
Authenticity and detection	Synthetic faces, images, art, radiographs, deepfakes	More realistic synthetic content was harder to identify; focal anomaly search did not guarantee accuracy	Realism, category, expertise, matching
Learning and integration	Summaries, feedback, GenAI search, help-seeking	Structured summaries supported learning; interactive support changed allocation more consistently than performance	Format, prior knowledge, interface, transfer
Professional and creative work	Design ideation, translation, advertising, architecture	AI redistributed effort across search, ideation, evaluation, and coordination	Workflow stage, relevance, complexity
Engagement	Visual, behavioral, evaluative, learning	Greater gaze often reflected effort or verification rather than acceptance	Actual versus perceived provenance

## Data Availability

No new data were created or analyzed in this study. Data sharing is not applicable to this article.

## Supplementary Materials

The following supporting information can be downloaded at https://www.mdpi.com/article/10.3390/jemr19050097/s1: Table S1: PECOS framework and operational eligibility criteria; Table S2A: Database coverage, final search dates, limits, and record counts; Table S2B: Confirmed Scopus search strategies; Table S3A: Primary reasons for full-text exclusion; Table S3B: Citation-level list of reports excluded following full-text eligibility assessment; Table S4A: Bibliographic, geographic, and sample characteristics; Table S4B: Study design, task, AI-generated exposure, comparator, and modality; Table S5A: Hardware, sampling, calibration, validation, and event detection; Table S5B: Preprocessing, AOIs, data quality, measures, and caveats; Table S6A: Fixation, orienting, and reinspection measures; Table S6B: Saccade, scanpath, transition, entropy, pupil, saliency, and derived measures; Table S7A: Cognitive processing, load, performance, and gaze–cognition relationships; Table S7B: Visual, behavioral, evaluative, and learning engagement; Table S8A: Participant, source, and additional moderators; Table S8B: Modality, task, quality, complexity, coherence, and matching; Table S9A: Compact MMAT profile and narrative appraisal; Table S9B: Full MMAT criterion-level audit trail; Table S10A: Hardware, sampling, calibration, validation, event detection, and AOIs; Table S10B: Preprocessing, missing data, exclusions, pupil processing, visual controls, multiplicity, and data availability; Figure S1: Eye-tracking reporting-completeness heatmap; Table S11A: Model, version, prompt, settings, generation date, and candidate count; Table S11B: Selection, editing, matching, availability, and reproducibility notes; Figure S2: AI-stimulus reproducibility heatmap; Table S12: Study-by-domain evidence map; Table S13: Completed PRISMA 2020 Checklist.

## Abbreviations

AIGC	Artificial intelligence-generated content
AI	Artificial intelligence
AOI	Area of interest
EEG	Electroencephalography
ET	Eye tracking
GenAI	Generative artificial intelligence
HCI	Human–computer interaction
LLM	Large language model
MMAT	Mixed Methods Appraisal Tool
PECOS	Population–Exposure–Comparator–Outcomes–Study design
PRISMA	Preferred Reporting Items for Systematic Reviews and Meta-Analyses
ROI	Region of interest
SWiM	Synthesis Without Meta-analysis
TTFF	Time to first fixation
