# Supplementary material for: Eye Tracking and AI-Generated Content: A Systematic Literature Review of Visual Attention, Cognitive Processing, and User Engagement
Source: J Eye Mov Res. 2026 Sep 4;19(5):97. doi: 10.3390/jemr19050097 (PMC13600100; doi:10.3390/jemr19050097)
Supplement: Supplementary file 1 [file jemr-19-00097-s001.zip › jemr-4489280-supplementary.pdf]

Journal of
Eye Movement Research

MDPI

Supplementary Materials

Eye Tracking and AI-Generated Content: A Systematic Literature Review of Visual Attention, Cognitive Processing, and User Engagement

Hakile Resulbegoviq, Valentina Hlebec, Mirjana Pejić Bach and Irina Stamatović

Scope: These supplementary materials document the review framework, search-strategy information, study-selection totals and citation-level exclusions, study characteristics, eye-tracking methods and measures, cognitive and engagement outcomes, moderators, criterion-level MMAT judgments, eye-tracking reporting completeness, AI-stimulus reproducibility, evidence mapping, and the completed PRISMA 2020 Checklist. Only the 23 final included studies are represented in the evidence matrices. NR indicates not reported in the examined full text.

S1. Review Framework and Eligibility Criteria

Supplementary Table S1. PECOS framework and operational eligibility criteria.

PECOS/domain	Inclusion rule	Exclusion rule
Population	Human participants of any age, expertise, or setting.	No human participants; model-only, benchmark, or simulation studies.
Exposure	Direct viewing, reading, evaluation, or task-based processing of identifiable AI-generated content, including text, images, summaries, feedback, subtitles, synthetic faces, medical images, video, deepfakes, or interactive outputs.	AI used only to analyze gaze, classify data, adapt an interface, or support a task without participant exposure to an identifiable generated output.
Comparator	Human-generated, teacher-generated, professionally produced, authentic/real, traditional, no-AI, or no-augmentation condition.	Only comparisons among AI models, prompts, personalization strategies, or generated variants without an eligible non-AI/human/real comparator.
Outcomes	Eye-tracking, gaze, or pupillometric outcomes, with associated cognitive, evaluative, behavioral, or learning outcomes where reported.	No eligible gaze outcome; gaze used only as system input.
Study design	Original comparative empirical research, including within-subject, between-subject, controlled laboratory, user, observational comparative, and mixed-methods studies.	Reviews, theoretical papers, editorials, preprints without a peer-reviewed version, posters, theses, book chapters, extended abstracts, late-breaking reports, and non-peer-reviewed reports.
Other limits	English-language, peer-reviewed journal articles or full conference papers; 2015 through the final search date in 2026;	Published before 2015, non-English, ineligible publication type

S2. Search-Strategy Documentation

Three databases were searched: Scopus, Web of Science Core Collection, and PubMed. The verified database totals were 503, 381, and 12 records, respectively (896 records in total). Searches were limited to English-language publications from 2015 through 2026. Three databases were searched: Scopus, Web of Science Core Collection, and PubMed. The final searches were conducted on 2 July 2026 and yielded 503, 381, and 12 records, respectively (896 records in total). Searches were limited to English-language publications from 2015 through 2026. Database coverage, platforms, search dates, limits, and record counts are reported in Supplementary Table S2A.

Supplementary Table S2A. Database coverage, final search dates, limits, and record counts.

Database	Platform	Search date	Limits	Records	Status
Scopus	Elsevier	2 July 2026	2015–2026; English	503	Confirmed
Web of Science Core Collection	Clarivate	2 July 2026	2015–2026; English	381	Confirmed

 https://doi.org/10.3390/jemr19050097

Database	Platform	Search date	Limits	Records	Status
PubMed	U.S. National Library of Medicine	2 July 2026	2015–2026; English	12	Confirmed

Supplementary Table S2B. Confirmed Scopus search strategies.

Version	Available draft query	Status
Broad	TITLE-ABS-KEY(("eye tracking" OR "eye-tracking" OR eyetracking OR "gaze tracking" OR gaze OR "eye movement*" OR "eye-movement*" OR fixation* OR saccad* OR "visual attention" OR "pupil dilation" OR pupillometr* OR scanpath* OR "scan path*" OR "dwell time" OR "reading time") AND ("AI-generated" OR "AI generated" OR "artificial intelligence generated" OR "machine-generated" OR "machine generated" OR "computer-generated" OR "computer generated" OR "algorithmically generated" OR "LLM-generated" OR "large language model*" OR LLM OR LLMs OR ChatGPT OR GPT OR "generative AI" OR GenAI OR "synthetic content" OR "synthetic media" OR "AI-authored" OR "AI authored" OR "machine-authored" OR "machine authored" OR "automated text" OR "automated journalism" OR deepfake*) AND (perception OR perceive* OR processing OR attention OR engagement OR readability OR credibility OR trust OR trustworthiness OR "cognitive load" OR "visual processing" OR "reading behavior" OR "reading behaviour" OR "user experience" OR preference OR authenticity OR "emotional response" OR "decision making")) AND PUBYEAR > 2014	Confirmed
Narrow	TITLE-ABS-KEY(("eye tracking" OR "eye-tracking" OR eyetracking OR "gaze tracking" OR "eye movement*" OR fixation* OR "pupil dilation" OR pupillometr* OR scanpath* OR "dwell time" OR "reading time") AND (("AI-generated" OR "AI generated" OR "machine-generated" OR "machine generated" OR "LLM-generated" OR "large language model*" OR ChatGPT OR GPT OR "generative AI" OR GenAI OR "AI-authored" OR "AI authored" OR "machine-authored" OR "machine authored" OR "synthetic media" OR deepfake*) W/5 (content OR text OR article* OR news OR image* OR face* OR advertisement* OR "social media" OR post* OR visual* OR design* OR video*)) AND ("human-generated" OR "human generated" OR "human-authored" OR "human authored" OR "human-written" OR "human written" OR "human-created" OR "human created" OR "human-made" OR "human made" OR authentic OR real)) AND PUBYEAR > 2014	Confirmed

The Scopus search strategies shown above were confirmed for the final review documentation.

S3. Full-Text Exclusions

Supplementary Table S3A. Primary reasons for full-text exclusion.

Primary reason	Reports (n)
No eligible comparator	28
Eye tracking did not assess direct processing of AI-generated content	12
Ineligible publication type or publication status	5
No direct exposure to AI-generated content	3
Non-empirical or no eligible human-participant study	2
Total excluded after full-text assessment	50

Supplementary Table S3B. Citation-level list of reports excluded following full-text eligibility assessment.

DOI	Source	Year	Title	Authors
10.1016/j.jclinane.2026.112163	Journal of Clinical Anesthesia	2026	Generative AI in preanesthetic consultations: Effects on efficiency, documentation workload, quality, and physician-patient interaction: A simulation trial	Rahrisch A.; Braun J.; Ganter M.T.; Roche T.R.; Spahn D.R.; Tscholl D.W.; Kaserer A.
10.1016/j.jdent.2023.104793	Journal of Dentistry	2024	Impact of artificial intelligence on dentists' gaze during caries detection: A randomized controlled trial	Arsiwala-Scheppach L.T.; Castner N.J.; Rohrer C.; Mertens S.; Kasneci E.; Cejudo Grano de Oro J.E.; Schwendicke F.
10.3758/s13423-025-02756-9	Psychonomic Bulletin & Review	2025	Sentence processing by humans and machines: Large language models as a tool to better understand human reading	Kaye N.G.; Gordon P.C.

DOI	Source	Year	Title	Authors
10.1007/s00266-024-04240-6	Aesthetic Plastic Surgery	2024	A Gaze into the Eyebrow Standards of Asian Versus Caucasian Women	Zeng R.; Moellhoff N.; Cotofana S.; Giunta R.; Wiggenhauser S.; Glaue E.; Yi K.-H.; Philipp-Dormston W.G.; Merkel C.; Frank K.
10.2196/59946	Journal of Medical Internet Research	2025	Effect of Artificial Intelligence Helpfulness and Uncertainty on Cognitive Interactions with Pharmacists: Randomized Controlled Trial	Tsai C.-C.; Kim J.Y.; Chen Q.; Rowell B.; Yang X.J.; Kontar R.; Whitaker M.; Lester C.
10.1145/3715669.3728427	ACM Symposium on Eye Tracking Research and Applications, ETRA 2025	2025	Investigating the Cognitive Differences in Scene Processing and Aesthetic Evaluation When Viewing Human- vs. AI-generated Imagery	Huang L.F.
10.1080/09544828.2026.2616582	Journal of Engineering Design	2026	A proposed Human-AI collaborative Kansei Engineering methodology for heritage-inspired designs: a study using surveying and eye-tracking with Africans and Asians	Atef S.; Ali I.; Romouzy-Ali A.; Kato M.; Eltawil A.B.
10.1145/3772363.3798752	CHI Conference Proceedings	2026	Overwhelmed by AI? How Task Difficulty and Creative Potential Moderate Cognitive Load in Human-AI Co-creation	Zhang C.; Ge Q.; Han T.
10.1145/3719160.3736633	CUI 2025 Proceedings	2025	DesignMinds: Enhancing Video-Based Design Ideation with a Vision-Language Model and a Context-Injected Large Language Model	He T.H.; Stankovic A.; Niforatos E.; Kortuem G.
10.1145/3649902.3655661	Proceedings of ETRA 2024	2024	SARA: Smart AI Reading Assistant for Reading Comprehension	Thaqi E.; Mantawy M.O.; Kasneci E.
10.1145/3772318.3790379	CHI Conference Proceedings	2026	Skin-Deep Bias: How Avatar Appearances Shape Perceptions of AI Hiring	Lau K.H.C.; Stark P.; Bozkir E.; Kasneci E.
10.1007/978-3-032-16284-7_37	Smart Innovation, Systems and Technologies	2026	First Insights into Neuromarketing Responses to Artificial Intelligence Versus Human-Crafted Ads	Castro A.V.C.; Alvarado-Herrera A.; Romero K.I.Q.; Sánchez Guzmán A.C.; Simonian H.
10.3390/bs16030349	Behavioral Sciences	2026	Human-like, Animal-like, or Object-like? The Impact of LLM-Based Virtual Doctor Avatar Design on User Emotion, Physiology, and Experience	Zhang H.; Wang S.Y.; Peng R.
10.1145/3772363.3798566	CHI Conference Proceedings	2026	When LLMs See Where You Struggle: A Gaze-to-Context	Bae G.; Park J.K.

DOI	Source	Year	Title	Authors
			Approach for Reader-Driven Reading Assistance	
10.1140/epjqt/s40507-025-00310-z	EPJ Quantum Technology	2025	AI support meets AR visualization for Alice and Bob: personalized learning based on individual ChatGPT feedback in an AR quantum cryptography experiment for physics lab courses	Coban A.; Dzsotjan D.; Küchemann S.; Durst J.; Kuhn J.; Hoyer C.
10.1145/3795011.3795058	Augmented Humans 2026	2026	Shared Gaze on AI Node Maps: Mitigating Empathy Fog in AI-Mediated Dyadic Discussion	Yamashita J.; Saito H.; Sakurada K.; Takashita S.; Muramoto G.; Inami M.
10.1007/978-981-95-4499-8_31	Communications in Computer and Information Science	2026	Effects of Personalized Generative AI on Student Creativity: Evidence from Creative Performance, Self-efficacy, and Attention Allocation	Chen Y.; Wu X.; Zhong Y.; Huang C.
10.1145/3725840	Proceedings of the ACM on Human-Computer Interaction	2025	OASST-ETC Dataset: Alignment Signals from Eye-tracking Analysis of LLM Responses	Lopez-Cardona A.; Idesis S.; Barreda-Ángeles M.; Abadal S.; Arapakis I.
10.1145/3750069.3750397	CHItaly 2025 Proceedings	2025	Eyes on the Code: Mapping Critical Thinking Through Eye-Tracking for Student-LLM Coding Interactions	Sqalli M.T.
10.16910/jemr.17.5.3	Journal of Eye Movement Research	2024	Understanding consumer perception and acceptance of AI art through eye tracking and BERT-based sentiment analysis	Yu T.; Xu J.; Pan Y.
10.3758/s13428-025-02677-4	Behavior Research Methods	2025	EMTeC: A corpus of eye movements on machine-generated texts	Bolliger L.S.; Haller P.; Cretton I.C.R.; Reich D.R.; Kew T.; Jäger L.A.
10.3233/faia250634	Frontiers in Artificial Intelligence and Applications	2025	Real-Time Gaze Awareness in Conversational Agents: Enhancing Collaboration and Personalization in VR Art Experiences	Javdani Rikhtehgar D.; Wang S.H.; Schlobach S.; Heylen D.
10.1145/3769994.3770034	Koli Calling 2025 Proceedings	2025	Student Engagement with GenAI's Tutoring Feedback: A Mixed Methods Study	Jacobs S.; Haas J.; Kiesler N.
10.1109/HRI61500.2025.10974124	ACM/IEEE International Conference on Human-Robot Interaction	2025	Evaluating Efficiency and Engagement in Scripted and LLM-Enhanced Human-Robot Interactions	Schreiter T.; Rüppel J.V.; Hazra R.; Rudenko A.; Magnusson M.; Lilienthal A.J.
10.1016/j.jag.2026.105151	International Journal of Applied Earth Observation	2026	An attention-aware map interaction framework based on eye tracking and generative models	Wang T.X.; Du P.; Dang P.; Liu T.; Li P.P.

DOI	Source	Year	Title	Authors
	and Geoinformation			
10.14236/ewic/bcshci2025.13	BCS HCI 2025	2025	From Queries to Prompts: Comparing User Experience in Generative AI Tools and Search Engines	Zubair M.; Alhassan M.A.; Bello F.
10.1080/10447318.2025.2454954	International Journal of Human-Computer Interaction	2025	Consumers' Emotional Responses to AI-Generated Versus Human-Generated Content: The Role of Perceived Agency, Affect and Gaze in Health Marketing	Diwanji V.S.; Geana M.; Pei J.; Nguyen N.; Izhar N.; Chaif R.H.
10.47989/ir31iconf64165	Information Research	2026	See, trust, and interact: how AI disclosure shapes high school students' trust	Chen N.; Lai Z.Y.; Liu Y.C.; Li J.; Wang R.; Yan P.
10.1145/3686215.3688382	ICMI 2024 Companion	2024	Detecting when Users Disagree with Generated Captions	Bhatti O.S.; Sriram H.; Selim A.M.; Conati C.; Barz M.; Sonntag D.
10.1145/3806019	Proceedings of the ACM on Human-Computer Interaction	2026	Investigating Mutual Gaze in Real-Time LLM-Driven Avatar Interaction on 2D Screens	Drewes H.; Dinc A.; Alt F.
10.1145/3789514.3792037	HotMobile 2026 Proceedings	2026	GazeSummary: Exploring Gaze as an Implicit Prompt for Personalization in Text-based LLM Tasks	Ding J.; Chen K.; Zhang Y.; Wang Y.; Liu X.; Patel S.; Gadre A.
10.5281/zenodo.15870207	International Conference on Educational Data Mining	2025	Examining the Role of LLM-Driven Interactions on Attention and Cognitive Engagement in Virtual Classrooms	Özdel S.; Sarpkaya C.; Bozkir E.; Gao H.; Kasneci E.
10.1016/j.caeai.2025.100489	Computers and Education: Artificial Intelligence	2025	Engagements with GPT responses and learner prompts in ChatGPT-based learning of English argumentative writing logic and their impacts	Zhang R.F.; Zou D.; Xie H.R.; Wang F.L.
10.1016/j.ijhcs.2025.103714	International Journal of Human-Computer Studies	2026	Understanding trust toward human versus AI-generated health information through behavioral and physiological sensing	Sun X.; Ma R.; Wei S.; Cesar P.; Bosch J.A.; El Ali A.
10.1016/j.paid.2025.113415	Personality and Individual Differences	2025	Loneliness, personality, and attention to AI-generated images depicting social threat: An eye-tracking study	Pfeifer J.; de Winter J.; Dodou D.; Eisma Y.B.
10.1109/FG61629.2025.11099302	IEEE FG 2025	2025	Analysis of Human Perception in Distinguishing Real and AI-Generated Faces: An Eye-Tracking Based Study	Huang J.; Gopalakrishnan S.; Mittal T.; Zuena J.; Pytlarz J.
10.3390/jtaer20040339	Journal of Theoretical and Applied	2025	Trust or Skepticism? Unraveling the Communication	Jiang S.F.; Zheng W.Q.; Kong H.Y.

DOI	Source	Year	Title	Authors
	Electronic Commerce Research		Mechanisms of AIGC Advertisements on Consumer Responses	
10.15199/48.2025.03.66	Przeglad Elektrotechniczny	2025	Eye tracking and exploring AI potential in describing and generating images: a contribution to research on generative art	Osińska V.; Szalach A.; Piotrowski D.M.; Martinez J.C.
10.36868/ijcs.2025.04.01	International Journal of Conservation Science	2025	Architectural Visual Design – Eye Tracking Analysis of Church Interiors Created Using Artificial Intelligence	Kocki W.; Dzieńkowski M.
10.1371/journal.pone.0326498	PLOS ONE	2025	Emotional impact of AI-generated vs. human-composed music in audiovisual media: A biometric and self-report study	Fišer N.; Martín-Pascual M.Á.; Andreu-Sánchez C.
10.1109/ACCESS.2024.3503059	IEEE Access	2024	Gaze-Driven Adaptive Learning System with ChatGPT-Generated Summaries	Santhosh J.; Dengel A.; Ishimaru S.
10.3390/bs15121684	Behavioral Sciences	2025	From Gaze to Music: AI-Powered Personalized Audiovisual Experiences for Children's Aesthetic Education	Liu J.H.; Liu J.; Yan H.
10.1145/3795926.3795982	ICADI 2025 Proceedings	2026	Research on Generative AI Creation Systems Based on Visual Language Modeling: Human-Machine Collaboration and Cognitive Feedback Mechanisms	Liu S.; Yim J.
10.1109/ACCESS.2026.3686928	IEEE Access	2026	A Usability Study of Interpretable Aesthetic Evaluation as Decision Support in Full-Site AI Website Generators for Novice Users	Shi H.L.; Chu C.
—	CAADRIA 2024 Proceedings	2024	Empirical Insights into Architectural Aesthetics: A Neuroscientific Perspective	Hartanto E.; Chen A.; Koh I.
10.1016/j.neuron.2026.04.005	Neuron	2026	Scaffolding human and AI instruction: Neural alignment and learning gains in online education	Peng Y.; Nastase S.A.; Huang Y.; Li Y.; Guo Z.; Li P.
10.65102/is2026780	Ingegneria Sismica	2026	Visual Symbol Construction in Generative AI Short Videos and the Mechanisms of User Attention Capture: Evidence from an Eye-Tracking Experiment	Zhao C.; Peng X.
10.1177/10711813251357920	Proceedings of the Human Factors and	2025	Eyes Don't Lie: Indifferent to AI-Generated Depression Screenings	Shahsavar Y.; Choudhury A.

DOI	Source	Year	Title	Authors
	Ergonomics Society			
—	International Conference on Computers in Education, ICCE 2024	2024	Enhancing Language Learning Through Multimodal AI-Driven Feedback on Picture Descriptions: An Eye-Tracking Study	Zhao R.B.; Xie Z.W.; Zhuang Y.P.; Li H.X.; Yu P.L.H.
—	Economics, Management & Business 2023	2023	Capturing Attention: Investigating the Impact of AI-Generated and Photographer-Captured Product Photos through Neuromarketing	Kusá A.; Belicková P.

The 27 reports that could not be retrieved are not included in this table because their eligibility could not be assessed.

One primary reason was assigned per report. The aggregate categories in Supplementary Table S3A sum to 50 full-text exclusions. Supplementary Table S3B provides the citation-level list of these 50 excluded reports. These materials are distinct from the PRISMA 2020 Checklist provided in Supplementary Appendix S13.

S4. Detailed Characteristics of Included Studies

Supplementary Table S4A. Bibliographic, geographic, and sample characteristics.

RefID	Study	Country / setting	Primary domain	Final N	Participants	Publication / venue	DOI
LR0026	Morita et al. (2025)	Germany	Educational technology / HCI / cognitive augmentation	24	University students or professionals based in Germany; diverse national backgrounds	Conference paper: The Augmented Humans International Conference 2025 (AHs 2025)	https://doi.org/10.1145/3745900.3746066
LR0039	Fu et al. (2026)	China	Industrial design / ergonomics / human–AI co-creativity	18	Design-trained participants, mostly junior designers; familiar with digital sketching	Journal article: Displays, 91, 103275	https://doi.org/10.1016/j.displa.2025.103275
LR0042	Cha et al. (2026)	Republic of Korea	Cognitive neuroscience / character design / UX	24	Healthy, right-handed university students	Journal article: Frontiers in Human Neuroscience, 20, 1723608	https://doi.org/10.3389/fnhum.2026.1723608
LR0123	Guan et al. (2025)	China and Republic of Korea	Brand communication / logo design / marketing	54	Adults recruited for the eye-tracking Study 2 in China and South Korea	Journal article: Frontiers in Communication, 10, 1667077	https://doi.org/10.3389/fcomm.2025.1667077
LR0125	Abu-Rayyash & Lacruz (2025)	Not explicitly reported	Audiovisual translation / subtitling / media accessibility	82	Native Arabic speakers randomly assigned to subtitle conditions	Journal article: Journal of Eye Movement Research, 18, Article 29	https://doi.org/10.3390/jemr18040029
LR0144	Komninos et al. (2024)	Not explicitly reported	HCI / mobile text entry / transcription-task methodology	13	Convenience sample from the student population of a local university	Conference paper: International Conference on Mobile and Ubiquitous Multimedia (MUM '24)	https://doi.org/10.1145/3701571.3701586
LR0152	Bozkir et al. (2024)	Not explicitly reported	Applied perception / HCI / synthetic faces	22	Adults with some computer-science familiarity; low prior familiarity with deepfakes	Journal article: ACM Transactions on Applied Perception, 22(2), Article 6	https://doi.org/10.1145/3696667

RefID	Study	Country / setting	Primary domain	Final N	Participants	Publication / venue	DOI
LR0166	Liu et al. (2026)	China	Cultural heritage / illustration / computational aesthetics	38	Graduate students and educators in art design, computer science and cultural digitization; all frequent AI users	Journal article: Electronics, 15, Article 15	https://doi.org/10.3390/electronics15010015
LR0167	Yfantidou et al. (2025)	United Kingdom	Health communication / advertising / behavioral science	42	UK adults in the bowel-screening age range; White and South Asian participants recruited with demographic quotas	Journal article: Information, 16, 935	https://doi.org/10.3390/info16110935
LR0172	Yun & Kim (2026)	Republic of Korea	Architecture / environmental design / retail UX	30	Adults recruited through university online communities and professional social networks	Journal article: Buildings, 16, 876	https://doi.org/10.3390/buildings16040876
LR0197	Yao et al. (2025)	Hong Kong SAR, China	Translation studies / translator education / post-editing	26	Postgraduate Master of Translation and Interpreting students from three Hong Kong universities	Journal article: The Interpreter and Translator Trainer	https://doi.org/10.1080/1750399X.2025.2535239
LR0202	Yuan et al. (2026)	China	Multimedia learning / educational technology / cognitive neuroscience	80	Undergraduate and graduate education-major students	Journal article: Education Sciences, 16, 39	https://doi.org/10.3390/educsci16010039
LR0228	Wong et al. (2025)	United States	Radiology / medical imaging / clinical decision support	16	Practicing radiologists from multiple specialties and experience levels	Conference paper: Symposium on Eye Tracking Research and Applications (ETRA '25)	https://doi.org/10.1145/3715669.3726789
LR0258	Gupta et al. (2020)	Not explicitly reported	Multimodal interaction / deepfake perception / HCI	40	Mostly undergraduate and graduate students; naïve to the experimental purpose	Conference paper: International Conference on Multimodal Interaction (ICMI '20)	https://doi.org/10.1145/3382507.3418857
LR0273	Cartella et al. (2024)	Not explicitly reported	Signal processing / computer vision / manipulated-image perception	20	Human participants; demographic and recruitment details NR	Journal article: IEEE Signal Processing Letters	https://doi.org/10.1109/LSP.2024.3375288
LR0280	Xu et al. (2026)	China	Science education / simulation-based learning / information search	57	University undergraduates from diverse majors with limited prior GenAI-search experience	Journal article: Education Sciences, 16, 769	https://doi.org/10.3390/educsci16050769
LR0317	Cao et al. (2025)	China / Macao SAR, China	EFL academic writing / feedback processing	10	Chinese undergraduates, non-English majors, Mandarin L1, without formal academic-writing training	Conference paper: 5th International Conference on Educational Technology (ICET 2025)	https://doi.org/10.1109/ICET67421.2025.11380530
LR0321	Cosentino et al. (2025)	Norway	Embodied mathematics / formative feedback / educational technology	30	School students with no prior experience of the body-scale number-line system or GenAI	Journal article: British Journal of Educational Technology	https://doi.org/10.1111/bjet.13587
LR0334	Zhou & Kawabata (2023)	Japan	Empirical aesthetics / visual perception / AI art	34	Japanese undergraduates naïve to art criticism	Journal article: i-Perception, 14(5)	https://doi.org/10.1177/20416695231209846
LR0386	Chen et al. (2025)	China	Learning analytics / help-seeking / ESL writing	38	Chinese university students learning English as a second language	Journal article: Computers & Education, 226, 105198	https://doi.org/10.1016/j.compedu.2024.105198
LR0525	Ilyas et al. (2025)	Denmark	Reading / HCI / AI-authored literary text	12	Technical University of Denmark students with	Conference paper: Symposium on Eye Tracking Research	https://doi.org/10.1145/3715669.3726846

RefID	Study	Country / setting	Primary domain	Final N	Participants	Publication / venue	DOI
					varied English proficiency	and Applications (ETRA '25)	
LR0558	Osińska et al. (2025)	Poland	Visual recognition / information and communication research	36	Adults from varied occupations; participants differed in digital-image work and AI-image-generation experience	Journal article: Journal of Eye Movement Research, 18, Article 61	https://doi.org/10.3390/jemr18060061
LR0560	Holmes et al. (2025)	Australia	Communication / media psychology / deepfake detection	32	Australian non-experts recruited through student channels and snowball networks	Journal article: Studies in Communication and Media, 14(4), 550–569	https://doi.org/10.5771/2192-4007-2025-4-550

Supplementary Table S4B. Study design, task, AI-generated exposure, comparator, and modality.

RefID	Study	Design	Primary task	AI-generated content / model	Comparator	Primary modality
LR0026	Morita et al. (2025)	Within-subject / repeated measures	Reading / transcription / subtitle viewing	AI-generated text summaries, sentence-aligned images, and image summaries; underlying stories and questions were also AI-generated [ChatGPT for stories, summaries and questions; DALL-E/diffusion model for images; CLIP for summary-image selection]	Baseline document without GenAI augmentation; underlying baseline narrative was still AI-generated	Multimodal / interactive
LR0039	Fu et al. (2026)	Hybrid within- and between-subject	Creative ideation / concept development	GPT-4.1 conversational ideation responses and Midjourney-generated visual stimuli [GPT-4.1; Midjourney]	Human designer collaboration; conventional image-search stimuli; no-additional-stimulus control	Multimodal / interactive
LR0042	Cha et al. (2026)	Within-subject / repeated measures	Free viewing / evaluation	AI-generated 2D and 3D female character faces across six depicted age categories [Midjourney version 6.1]	Licensed real facial photographs standardized for pose, background, size, luminance and contrast	Static visual
LR0123	Guan et al. (2025)	Within-subject / repeated measures	Free viewing / evaluation	AI-generated brand logos varying in innovation and semantic relevance [ChatGPT-4o and Midjourney version 5.2]	Human-created logos at corresponding innovation and semantic-relevance levels	Static visual
LR0125	Abu-Rayyash & Lacruz (2025)	Between-subject	Reading / transcription / subtitle viewing	Arabic subtitles generated by an LLM and presented over audiovisual material [GPT-4o (March 2025); no post-editing reported]	Professionally produced Amazon Prime Arabic subtitles	Textual / linguistic
LR0144	Komninos et al. (2024)	Within-subject / repeated measures	Reading / transcription / subtitle viewing	LLM-generated conversational prompt–response context surrounding transcription phrases [Llama 3 8B Instruct]	Baseline transcription phrase without LLM-generated conversational context	Textual / linguistic
LR0152	Bozkir et al. (2024)	Hybrid within- and between-subject	Detection / authenticity classification	Synthetic face images generated by successive GAN architectures [PGGAN, StyleGAN and StyleGAN2]	Real face images	Static visual
LR0166	Liu et al. (2026)	Within-subject / repeated measures	Free viewing / evaluation	AI-generated Tibetan children's-book script, prompts and illustrations [ERNIE / WenxinYiyan 4.5 Turbo text and text-to-image functions]	Human-drawn illustrations created from the same ERNIE-generated script and prompts	Static visual
LR0167	Yfantidou et al. (2025)	Within-subject / repeated measures	Free viewing / evaluation	AI-generated advertising images [Specific image-generation model NR]	Hand-drawn images and older stock images	Static visual
LR0172	Yun & Kim (2026)	Within-subject / repeated measures	Free viewing / evaluation	AI-generated biophilic façade variations [Stable Diffusion XL with ControlNet Depth]	High-resolution photograph of an existing automated-retail storefront	Static visual
LR0197	Yao et al. (2025)	Within-subject / repeated measures	Post-editing	GPT-4 chain-of-thought reasoning and post-editing suggestions [GPT-4]	Traditional post-editing without GPT-generated suggestions	Textual / linguistic
LR0202	Yuan et al. (2026)	Between-subject	Learning	LLM-generated mind-map summaries and text summaries synchronized with instructional video [Doubao large-language-model pedagogical agent]	Original instructional video without pedagogical-agent summary	Multimodal / interactive

RefID	Study	Design	Primary task	AI-generated content / model	Comparator	Primary modality
LR0228	Wong et al. (2025)	Within-subject / repeated measures	Detection / authenticity classification	Synthetic chest X-ray images conditioned on radiology reports [RoentGen latent diffusion model]	Corresponding real MIMIC-CXR chest X-rays and reports	Static visual
LR0258	Gupta et al. (2020)	Within-subject / repeated measures	Detection / authenticity classification	Deepfake videos from the Google/Jigsaw DeepFake Detection dataset [Specific generation model NR]	Real videos	Audiovisual / deepfake video
LR0273	Cartella et al. (2024)	Within-subject / repeated measures	Detection / authenticity classification	Partially manipulated images created through inpainting and instruction-guided editing [Stable Diffusion 2.0 inpainting and InstructPix2Pix]	Original real images	Static visual
LR0280	Xu et al. (2026)	Between-subject	Learning	RAG-supported generated answers to learner search queries [GLM-4 and Qwen2 with retrieval-augmented generation]	Baidu traditional web search	Multimodal / interactive
LR0317	Cao et al. (2025)	Within-subject / repeated measures	Feedback processing / help-seeking	Multidimensional academic-writing feedback [DeepSeek-R1]	Feedback generated by two professional teacher mentors	Textual / linguistic
LR0321	Cosentino et al. (2025)	Between-subject	Learning	Personalized formative hints and suggestions displayed as text [GPT-4 API]	Human instructor feedback	Textual / linguistic
LR0334	Zhou & Kawabata (2023)	Within-subject / repeated measures	Free viewing / evaluation	AI-generated landscape paintings [Disco Diffusion]	Human-made paintings from the Vienna Art Picture System	Static visual
LR0386	Chen et al. (2025)	Between-subject	Feedback processing / help-seeking	Interactive ChatGPT responses to context-specific help requests [ChatGPT 4.0]	Experienced human teacher/expert providing one-to-one chat support	Textual / linguistic
LR0525	Ilyas et al. (2025)	Within-subject / repeated measures	Reading / transcription / subtitle viewing	LLM-generated passages imitating three human authors' styles [Microsoft Bing Copilot]	Human-authored passages by the same three authors	Textual / linguistic
LR0558	Osińska et al. (2025)	Within-subject / repeated measures	Detection / authenticity classification	AI-generated architecture, art, car, landscape and pet images plus synthetic faces [GPT-4o image generation; ThisPersonDoesNotExist for faces]	Authentic photographs from Creative Commons, professional and private sources	Static visual
LR0560	Holmes et al. (2025)	Within-subject / repeated measures	Detection / authenticity classification	Face-replacement deepfake videos [Deepfake Detection Challenge (DFDC) stimuli; exact generation model NR]	Authentic videos	Audiovisual / deepfake video

S5. Detailed Eye-Tracking Methodology

Supplementary Table S5A. Hardware, sampling, calibration, validation, and event detection.

RefID	Study	Device family	Device / platform	Tracking mode	Sampling rate	Calibration	Validation / drift check	Fixation / saccade filter
LR0026	Morita et al. (2025)	Tobii	Tobii Pro eye tracker; model NR	Screen-based remote hardware	90 Hz	Calibration performed before recording; participants looked at calibration dots; point count and acceptance criteria NR	NR	Custom dispersion-threshold/I-DT procedure: 9 consecutive samples within 50 px; subsequent dispersion threshold 80 px; minimum fixation 100 ms
LR0039	Fu et al. (2026)	Tobii	Tobii Pro Fusion	Screen-based remote hardware	120 Hz	Eye-tracker calibration procedure NR; pre-task baseline/rest measurements were reported for physiological normalization	NR	Fixation/saccade event-detection algorithm NR
LR0042	Cha et al. (2026)	Tobii	Tobii Pro Spectrum	Screen-based remote hardware	1,200 Hz	Pre-measurement eye-tracker calibration/quality check reported; point count and numerical acceptance criteria NR	Participants were excluded if gaze tracking was insufficiently stable; all 24 final participants completed the study	Fixation-detection algorithm and thresholds NR

RefID	Study	Device family	Device / platform	Tracking mode	Sampling rate	Calibration	Validation / drift check	Fixation / saccade filter
LR0123	Guan et al. (2025)	Tobii	Tobii Pro Nano	Screen-based remote hardware	60 Hz	Nine-point calibration; a 3,000-ms central fixation correction preceded the first-stage display	No numerical calibration-acceptance threshold reported	Tobii Pro Lab used; fixation-detection algorithm and thresholds NR
LR0125	Abu-Rayyash & Lacruz (2025)	RealEye	RealEye webcam-based platform	Web-based webcam eye tracking	30 Hz	Automated 39-point calibration; repeated automatically when precision thresholds were not met; viewing distance approximately 60 cm	Average system accuracy 1.5–2.0° visual angle; equipment checks required webcam ≥720p, stable ≥5 Mbps internet and adequate front lighting	I-VT; velocity threshold 150°/s; reported minimum-fixation range 80–400 ms; 200-ms noise-reduction window
LR0144	Komninos et al. (2024)	Tobii	Tobii T120 integrated monitor	Screen-based remote hardware	NR (device model reported as Tobii T120)	Individual eye-tracker calibration conducted before the experiment; exact procedure and thresholds NR	NR	Fixation-detection algorithm and thresholds NR
LR0152	Bozkir et al. (2024)	Tobii	Tobii Pro Spectrum	Screen-based remote hardware with chin rest	600 Hz	Five-point calibration before each recording phase	NR	I-VT; velocity threshold 30°/s; minimum fixation duration 60 ms
LR0166	Liu et al. (2026)	Tobii	Tobii Pro eye tracker; model NR	Screen-based remote hardware	NR	NR	NR	Fixation-detection algorithm and thresholds NR
LR0167	Yfantidou et al. (2025)	RealEyes	RealEyes remote webcam platform	Web-based webcam eye tracking	NR	Nine-point calibration/validation; mean error ≤0.6°, maximum <1.0°; recalibration for drift >1.0° or approximately every 12 trials	500-ms pre-stimulus drift check; trials flagged for drift >1.0°	I-VT 30°/s; minimum fixation ≥60 ms; adjacent fixations merged if separated by <75 ms and <0.5°; AOI visit required ≥100-ms dwell
LR0172	Yun & Kim (2026)	iMotions	iMotions Online/WebET with participant webcam	Web-based webcam eye tracking	30.19 Hz	Thirteen-point calibration; required accuracy within 1.3°	Final mean accuracy 0.8° and reported tracking-quality ratio 98%	Platform-derived fixation events; algorithm and thresholds NR
LR0197	Yao et al. (2025)	EyeLink	EyeLink 1000 Plus	Screen-based tower system with head stabilization	1,000 Hz	Thirteen-point calibration at session start; one-point drift check before each trial	NR	Event-detection algorithm NR; EyeLink Data Viewer used for fixation cleaning
LR0202	Yuan et al. (2026)	Unspecified	Eye tracker model/manufacturer NR	Screen-based; model NR	1,000 Hz	NR	Sub-pixel accuracy claimed; calibration/validation procedure NR	Fixation-detection algorithm and thresholds NR
LR0228	Wong et al. (2025)	EyeLink	EyeLink 1000 Plus	Screen-based tower system	500 Hz	Thirteen-point calibration and validation	NR	Fixations classified when saccade velocity was <30°/s and acceleration <8,000°/s²
LR0258	Gupta et al. (2020)	Tobii	Tobii TX300	Screen-based remote hardware	300 Hz	Exact calibration procedure NR; experiment divided into four 25-trial blocks to reduce calibration error and fatigue	NR	EyeMMV two-step spatial-dispersion procedure for fixation/saccade discovery; exact thresholds NR
LR0273	Cartella et al. (2024)	Unspecified	Screen-based eye tracker; model NR	Screen-based; model NR	NR	Eye tracker calibrated at the beginning of each experiment; point count and acceptance criteria NR	NR	Velocity-threshold fixation identification (I-VT); numerical velocity threshold NR
LR0280	Xu et al. (2026)	EyeLink	EyeLink 1000 Plus	Screen-based tower system with head stabilization	NR	Nine-point calibration and validation before the experiment	Participants with poor calibration or low valid-gaze ratio were excluded; numerical cutoffs NR	EyeLink Data Viewer preprocessing; fixation/saccade event-detection algorithm and thresholds NR
LR0317	Cao et al. (2025)	EyeLink	EyeLink 1000 Plus	Screen-based tower system	NR	NR	NR	Fixation-detection algorithm and thresholds NR
LR0321	Cosentino et al. (2025)	Tobii	Tobii mobile eye-tracking glasses; specific model NR	Mobile/head-mounted glasses	50 Hz gaze; paper	One-point calibration	NR	Fixation/saccade event-detection algorithm and thresholds NR

RefID	Study	Device family	Device / platform	Tracking mode	Sampling rate	Calibration	Validation / drift check	Fixation / saccade filter
					separately states pupil diameter captured at 250 Hz			
LR0334	Zhou & Kawabata (2023)	Tobii	Tobii Pro Spectrum	Screen-based remote hardware with chin rest	300 Hz	Nine-point calibration and validation; recalibration after each 20-stimulus break or if central fixation failed	Central fixation check before each trial	Tobii velocity-threshold identification fixation filter; exact numerical thresholds NR
LR0386	Chen et al. (2025)	Tobii	Tobii Nano Pro	Screen-based remote hardware	NR	NR	NR	No conventional fixation filter reported; gaze coordinates were overlaid on synchronized screen recordings for process coding
LR0525	Ilyas et al. (2025)	Tobii	Tobii 4C	Screen-based remote hardware	90 Hz	Individual calibration using Tobii software before the six trials; exact point count/threshold NR	Visual inspection of gaze coordinates, identified fixations and derived velocity	I2MC; fixation candidates >40 ms and <0.7° separation retained; candidates <40 ms excluded; saccades checked against main-sequence duration/amplitude criteria
LR0558	Osińska et al. (2025)	GazePoint	GazePoint GP3 HD	Screen-based remote hardware	150 Hz	Double nine-point calibration	NR	Gazepoint Analysis Standard Edition derived fixations/saccades; algorithm and thresholds NR
LR0560	Holmes et al. (2025)	Pupil Labs	Pupil Invisible glasses	Mobile/head-mounted glasses	NR in main paper; equipment specifications referred to OSF supplement	Glasses calibrated to each participant; procedure/acceptance criteria NR	NR	Processed in Pupil Labs/iMotions 10; fixation/saccade algorithm and thresholds NR

Supplementary Table S5B. Preprocessing, AOIs, data quality, measures, and caveats.

RefID	Study	Preprocessing / signal handling	Formal AOIs	Data-quality thresholds / exclusions	Measures captured	Key methodological caveat	Evidence location
LR0026	Morita et al. (2025)	AOI ratio calculated from fixation duration; no interpolation, smoothing, or formal track-loss rule reported	Document/main text; generated image; text summary; image summary	No explicit valid-gaze threshold or participant/trial exclusion rule reported; all 24 participants appear in the analysis	Fixation duration; AOI fixation-duration ratio; gaze scanpaths; heatmaps	Baseline meant no GenAI augmentation, but the underlying stories and questions were themselves AI-generated	Sections 3.5, 4.3 and 5.3; Figures 9–11
LR0039	Fu et al. (2026)	Baseline correction and within-participant normalization for pupil measures; no appreciable data loss reported after preprocessing	AOI-left and AOI-right; left contained collaborator/AI/stimulus and right contained brainstorming or design canvas; 1:2 area ratio	No numerical gaze-validity threshold reported; authors stated that no appreciable data loss occurred	Fixation count; fixation duration; heatmaps; baseline-corrected pupil diameter; cumulative pupil dilation; gaze trajectory length	The ideation and development phases used different comparison structures, and session-level dependence complicates interpretation	Sections 2.4, 3.3 and 5.1–5.4; Figures 3–4
LR0042	Cha et al. (2026)	Condition-level fixation and pupil measures computed within facial AOIs; detailed event preprocessing NR	Whole facial stimulus; feature-level eyes and mouth AOIs used for heatmaps	Qualitative stability criterion reported; no numerical gaze-quality threshold	Total fixation duration; average fixation duration; fixation count; average pupil diameter; heatmaps	Only one stimulus represented each age-by-image-type cell, leaving stimulus identity partly confounded with condition	Sections 2.1–2.5 and 3.2; Tables 1–2; Figure 6
LR0123	Guan et al. (2025)	Six of 60 samples excluded for low sampling rate; threshold	Eight logo AOIs spanning AI/human creator × innovation × semantic	Six samples excluded for low sampling rate;	Fixation count; total fixation duration; time to first fixation;	The unlabeled phase always preceded the labeled phase,	Sections 4.1–4.4; Figures 5–8; Table 6

RefID	Study	Preprocessing / signal handling	Formal AOIs	Data-quality thresholds / exclusions	Measures captured	Key methodological caveat	Evidence location
		defining low sampling rate NR	relevance; creator labels added in the second phase	no exact valid-gaze criterion	heatmaps; average fixation duration; custom start/end and start–end variation ratios	creating possible order and carryover effects	
LR0125	Abu-Rayyash & Lacruz (2025)	Raw coordinates filtered, fixations mapped to AOIs, and condition metrics derived	Subtitle strip at bottom of frame; picture/narrative area comprising the remainder	Very Low quality = 0 Hz/no usable fixations; Low = ≥1 Hz with <50% fixations; 25 of 107 datasets excluded	Fixation count; mean fixation duration; total fixation time/dwell; gaze proportion; TTFF; K-coefficient	The generated element was subtitle text; the underlying television episode was human-produced	Sections 3.1–3.4 and 4.1–4.4; Tables 2, 4–6
LR0144	Komninos et al. (2024)	NR	Blue prompt bubble and purple response bubble	No explicit track-loss, trial-exclusion or participant-exclusion threshold reported	Total fixation duration; fixation count; prompt share of prompt+response fixation duration; heatmaps	Only Study 1 used eye tracking; sampling frequency was not explicitly reported	Study 1, Sections 3.1–3.4; Figures 2–4
LR0152	Bozkir et al. (2024)	Missing gaze periods <100 ms considered; up to 22-ms gaps linearly interpolated; 30-ms median smoothing	No formal AOI partition in the main quantitative analysis; participants reported facial cues such as hair, eyes, ears and background	All 22 participants analyzed; explicit signal interpolation/smoothing described, but no participant-level quality threshold	Fixation rate; saccade length; viewing/rating time; explicit feature explanations	The paper refers to visual scanpaths but operationalizes them through fixation rate and saccade length rather than a dedicated scanpath-similarity measure	Sections 3.2–3.6 and 4.1–4.3; Figures 4–5
LR0166	Liu et al. (2026)	Very brief gaze lengths during source judgment were described as lacking interpretive value; formal exclusion cutoff NR	No standardized AOI scheme reported; analyses/heatmaps focused on faces, cultural-detail regions, supporting characters and text	No formal valid-gaze or track-loss threshold reported	Gaze/fixation duration; scanning range; heatmaps; clicks; questionnaire and interview responses	Eye-tracking apparatus and preprocessing were underreported, and stimulus-production conditions were not fully matched	Sections 3.4 and 4.1–4.3; Figures 7 and 11
LR0167	Yfantidou et al. (2025)	No interpolation of gaps ≤100 ms; remaining micro-saccade-like events <60 ms removed; 99th-percentile winsorization; duration outcomes log-transformed	Slogan; people/faces; objects	Trial excluded for >40% track loss; participant excluded for >25% total track loss	TTFF; total AOI fixation duration/dwell; fixation count; revisit count	Image type incorporated unavoidable differences in luminance, contrast and visual complexity	Sections 2.2–2.4 and 3.1–3.4; Tables 1–6
LR0172	Yun & Kim (2026)	Two-stage iMotions quality filtering; gaze/facial completion ≥90%; min–max normalization of gaze metrics	AOI 1 glass façade; AOI 2 signage sides; AOI 3 main signage area	Accuracy ≤1.3° and data completion ≥90%; 18 of 48 participants excluded across primary/external quality filters	Dwell time; average fixation duration; fixation count; revisit count; heatmaps	Findings were largely AOI-descriptive, and webcam-based precision varied across participants	Sections 3.4–3.9 and 4.1; Table 4; Figures 5–6
LR0197	Yao et al. (2025)	Fixations <100 ms or >1,200 ms removed; observations outside mean ±2.5 SD excluded	Source text and target text; AI suggestions/chain-of-thought were present in the interface but not the primary normalized gaze AOIs	Explicit fixation-duration and outlier-removal criteria; no participant-level valid-gaze threshold reported	Source- and target-text fixation duration per word; fixation count per word; keylogging and retrospective interviews	The AI output was embedded in a post-editing workflow; gaze was not isolated to the suggestion panel	Sections 4.3–4.4 and 5.3; Table 7
LR0202	Yuan et al. (2026)	NR	A1 video-content area; A2 summary area	No gaze-validity threshold, track-loss criterion or eye-tracking exclusion rule reported	Total fixation duration/time; fixation-time percentage; heatmaps; qualitative scanning patterns	Device model, calibration and fixation-filter details were not reported	Sections 4.2–4.3, 5.3.2 and 6.3; Table 4; Figure 4
LR0228	Wong et al. (2025)	Bias/fixation maps created for first, last, longest and shortest fixations; participant-level quality thresholds NR	No named AOIs; whole-radiograph fixation and saliency/bias maps	No explicit missing-gaze or participant/trial exclusion threshold	Saccade amplitude/direction; first/last/longest/shortest fixation duration; bias maps; correlation, KL divergence and similarity saliency metrics	Repeated observations were not clearly modeled at both participant and image levels	Sections 3.1–3.5; Tables 1–3; Figure 3
LR0258	Gupta et al. (2020)	Some gaze files lost due to eye closure/tracker error; missing-data quantity reported descriptively, without a	No formal AOIs; whole video and manipulated face/mouth region interpreted through gaze heatmaps	Data loss acknowledged but no numerical quality threshold	Fixation count; mean fixation duration; fixation entropy; number of re-fixations; scanpath length;	Video exposure counts were unequal and the exact event-detection thresholds were not reported	Sections 3.1–3.2; Figures 1 and 3

RefID	Study	Preprocessing / signal handling	Formal AOIs	Data-quality thresholds / exclusions	Measures captured	Key methodological caveat	Evidence location
		formal exclusion threshold			heatmaps/saliency maps		
LR0273	Cartella et al. (2024)	Fixations convolved with a fovea-sized Gaussian kernel to create saliency maps	No formal AOIs; saliency maps captured whole-image gaze concentration	No track-loss, trial-exclusion or participant-exclusion criterion reported	Recorded scanpaths; fixations; saliency maps; saliency-map entropy	The eye-tracker model, sampling rate and I-VT threshold were not reported	Section II.C and Section III, Saliency Entropy; Figures 1–2
LR0280	Xu et al. (2026)	Blinks, temporary gaze loss and device interference removed; trials with excessive data loss or continuously unstable gaze excluded	Video lecture; virtual simulation experiment; search-engine area	Qualitative exclusion for poor calibration, low valid-gaze ratio, excessive data loss or unstable recording; 9 of 66 participants excluded	AOI dwell time; fixation count; saccade count between AOIs; behavioral transition sequences	The eye-tracking analysis operated at broad interface-AOI level rather than direct word-level processing of generated answers	Sections 3.4–3.6 and 4.1; Tables 2–3; Figure 4
LR0317	Cao et al. (2025)	Metrics normalized by feedback word count	GenAI feedback, teacher feedback and the student's original abstract; analyses also separated four feedback dimensions	No gaze-validity, track-loss or exclusion threshold reported; all 10 participants analyzed	Fixation duration per word; fixation count/points per word; AOI backtracks per word; heatmaps	GenAI feedback was always displayed on the left and teacher feedback on the right	Sections IV.C–D and V; Figures 3–4; Tables I–II
LR0321	Cosentino et al. (2025)	Homography mapped mobile field-of-view gaze to common coordinates; first 30 s used for pupil normalization; brightness normalization; blink-related pupil gaps interpolated; wavelet-based pupillary activity	Feedback provider; task text; correct option; incorrect option; projected number line	Four of 34 students excluded for incomplete multimodal data; no numerical valid-gaze threshold	Proportion of time in AOIs; AOI transition proportions; fixation/saccade pattern–based information processing index; pupil-activity cognitive-load index	The paper reports 50-Hz gaze sampling but separately states 250-Hz pupil capture; the device/event-processing description is not fully reconciled	Data collection and preprocessing; Measurements; Figures 3–9
LR0334	Zhou & Kawabata (2023)	Tobii Pro Lab fixation metrics; all 1,360 free-viewing trials analyzed	No predefined AOIs; whole-painting metrics and fixation-duration heatmaps	No participant/trial exclusion threshold reported; all trials retained	Total fixation duration/dwell; fixation count; mean fixation duration; heatmaps	Participants were not told the source before viewing, and classification accuracy for AI art was low	Apparatus, Procedure and Data Analysis; Free-Viewing Results; Discussion
LR0386	Chen et al. (2025)	Trace, gaze and conversation streams synchronized into screen recordings; three researchers coded segmented videos; Krippendorff's alpha 0.82 for 50% double-coded videos	Dynamic interface ROIs: ChatGPT/teacher dialog box, reply messages, essay-writing tool and reading/essay content	No numerical gaze-quality threshold reported; all 38 participants appear in analysis	ROI gaze stays; back-and-forth gaze movements; multimodal stage/activity coding; first-order Markov/process-transition models	These findings are process-level, not conventional eye-movement effect estimates	Sections 3.2–3.3 and 4.1–4.2; Figures 3–6; Tables 3–5
LR0525	Ilyas et al. (2025)	Adaptive interpolation/filtering in I2MC; Savitzky–Golay velocity estimate; trials <10 s or without a reading pattern across ≥3 successive lines excluded; participant excluded if ≤2 valid trials	No formal AOIs; whole-text reading	Explicit trial and participant criteria; one participant and 12 low-quality trials excluded, leaving 12 participants/66 trials	Mean fixation duration; fixation rate; mean saccadic amplitude; mean pupil size; regression ratio	Final sample was 12 participants, and textual difficulty differed across passages/authors	Sections 2.2–2.6 and 3.1–3.4; Tables 1–2; Figure 2
LR0558	Osińska et al. (2025)	Zero-value rows/columns removed; fixation subset retained; saccade magnitude/vector derived between current and prior fixations	Faces: eyes, nose, ears, hair, neck/mouth; pets: muzzle, body, paws/background; cars: logo/plate, wheels, driver/interior, road/background; landscapes and architecture: structural/environmental regions	No numerical gaze-validity or track-loss threshold reported; all 36 participants analyzed	Fixation count; mean fixation duration; saccade magnitude/vector; gaze paths; AOI viewing-time percentage; average AOI revisits; heatmaps	AI and real images came from different source pools and were not fully matched on low-level visual properties	Methods 4; Sections 5.2–5.3 and 6–7; Tables 3–6 and A5
LR0560	Holmes et al. (2025)	Dynamic AOIs manually repositioned frame-by-frame to follow the moving face/body; practice trials excluded	Screen, body, head, eyes and mouth; reported inferential results focused on eyes, mouth and body	No numerical data-quality or trial-exclusion threshold in the main paper; further technical details referred to OSF	Average fixation duration; fixation count; saccade count; AOI fixation distributions	Sampling rate and event-processing parameters were not reported in the main article	Study 2, Sections 4.1–4.3; Table 3; Figures 1–2

S6. Eye-Tracking Measure Matrix

Supplementary Table S6A. Fixation, orienting, and reinspection measures.

RefID	Study	Fixation count	Mean fixation duration	Total fixation / dwell	Fixation rate	TTFF	First-fixation distribution	Revisit / re-fixation	Regression / backtrack
LR0026	Morita et al. (2025)	No	No	Yes	No	No	No	No	No
LR0039	Fu et al. (2026)	Yes	Yes	No	No	No	No	No	No
LR0042	Cha et al. (2026)	Yes	Yes	Yes	No	No	No	No	No
LR0123	Guan et al. (2025)	Yes	Yes	Yes	No	Yes	No	No	No
LR0125	Abu-Rayyash & Lacruz (2025)	Yes	Yes	Yes	No	Yes	No	No	No
LR0144	Komninos et al. (2024)	Yes	No	Yes	No	No	No	No	No
LR0152	Bozkir et al. (2024)	No	No	No	Yes	No	No	No	No
LR0166	Liu et al. (2026)	No	No	Yes	No	No	No	No	No
LR0167	Yfantidou et al. (2025)	Yes	No	Yes	No	Yes	No	Yes	No
LR0172	Yun & Kim (2026)	Yes	Yes	Yes	No	No	No	Yes	No
LR0197	Yao et al. (2025)	Yes	No	Yes	No	No	No	No	No
LR0202	Yuan et al. (2026)	No	No	Yes	No	No	No	No	No
LR0228	Wong et al. (2025)	No	Yes	No	No	No	Yes	No	No
LR0258	Gupta et al. (2020)	Yes	Yes	No	No	No	No	Yes	No
LR0273	Cartella et al. (2024)	No	No	No	No	No	No	No	No
LR0280	Xu et al. (2026)	Yes	No	Yes	No	No	No	No	No
LR0317	Cao et al. (2025)	Yes	No	Yes	No	No	No	No	Yes
LR0321	Cosentino et al. (2025)	No	Yes	Yes	No	No	No	No	No
LR0334	Zhou & Kawabata (2023)	Yes	Yes	Yes	No	No	No	No	No
LR0386	Chen et al. (2025)	No	No	Qualitative	No	No	No	Qualitative	No
LR0525	Ilyas et al. (2025)	No	Yes	No	Yes	No	No	No	Yes
LR0558	Osińska et al. (2025)	Yes	Yes	No	No	No	No	Yes	No
LR0560	Holmes et al. (2025)	Yes	Yes	No	No	No	No	No	No

Yes = analyzed as an outcome; Qualitative = used descriptively/process-wise; No = not reported as an analyzed outcome.

Supplementary Table S6B. Saccade, scanpath, transition, entropy, pupil, saliency, and derived measures.

RefID	Study	Saccade count	Saccade amplitude / length	Scanpath / gaze trajectory	AOI transitions	Entropy	Pupil diameter	Pupil dilation / change	K-coefficient	Information processing index	Gaze alignment	Saliency-map metrics	Heatmaps	Custom / other derived measure details
LR0026	Morita et al. (2025)	No	No	Yes	No	No	No	No	No	No	No	No	Yes	AOI fixation-duration ratio
LR0039	Fu et al. (2026)	No	No	Yes	No	No	Yes	Yes	No	No	No	No	Yes	BCPD, CPD, gaze asymmetry and GTL
LR0042	Cha et al. (2026)	No	No	No	No	No	Yes	No	No	No	No	No	Yes	No
LR0123	Guan et al. (2025)	No	No	No	No	No	No	No	No	No	No	No	Yes	Start/end and start–end variation ratios
LR0125	Abu-Rayyash & Lacruz (2025)	No	Yes	No	No	No	No	No	Yes	No	No	No	No	Gaze-proportion distribution
LR0144	Komninos et al. (2024)	No	No	No	No	No	No	No	No	No	No	No	Yes	Prompt share of fixation duration
LR0152	Bozkir et al. (2024)	No	Yes	No	No	No	No	No	No	No	No	No	No	Viewing/rating time
LR0166	Liu et al. (2026)	No	No	Qualitative	No	No	No	No	No	No	No	No	Yes	Scanning range and clicks
LR0167	Yfantidou et al. (2025)	No	No	No	No	No	No	No	No	No	No	No	No	No
LR0172	Yun & Kim (2026)	No	No	No	No	No	No	No	No	No	No	No	Yes	No
LR0197	Yao et al. (2025)	No	No	No	No	No	No	No	No	No	No	No	No	Fixation duration/count per word
LR0202	Yuan et al. (2026)	No	No	Qualitative	No	No	No	No	No	No	No	No	Yes	Fixation-time percentage
LR0228	Wong et al. (2025)	No	Yes	No	No	No	No	No	No	No	No	Yes	Yes	First/last/longest/shortest fixation profiles
LR0258	Gupta et al. (2020)	No	No	Yes	No	Yes	No	No	No	No	No	Yes	Yes	Scanpath length and fixation entropy

RefID	Study	Saccade count	Saccade amplitude / length	Scanpath / gaze trajectory	AOI transitions	Entropy	Pupil diameter	Pupil dilation / change	K-coefficient	Information processing index	Gaze alignment	Saliency-map metrics	Heatmaps	Custom / other derived measure details
LR0273	Cartella et al. (2024)	No	No	Yes	No	Yes	No	No	No	No	No	Yes	Yes	Saliency entropy
LR0280	Xu et al. (2026)	Yes	No	No	Yes	No	No	No	No	No	No	No	No	Behavioral sequence transitions
LR0317	Cao et al. (2025)	No	No	No	No	No	No	No	No	No	No	No	Yes	Per-word normalized metrics
LR0321	Cosentino et al. (2025)	No	Yes	No	Yes	No	No	Yes	No	Yes	No	No	No	Wavelet pupillary activity / cognitive-load index
LR0334	Zhou & Kawabata (2023)	No	No	No	No	No	No	No	No	No	No	No	Yes	No
LR0386	Chen et al. (2025)	No	No	Qualitative	Qualitative	No	No	No	No	No	No	No	No	ROI-stay and process-transition coding
LR0525	Ilyas et al. (2025)	No	Yes	No	No	No	Yes	No	No	No	No	No	No	Regression ratio
LR0558	Osińska et al. (2025)	No	Yes	Yes	No	No	No	No	No	No	No	No	Yes	AOI viewing-time percentage and saccade vector
LR0560	Holmes et al. (2025)	Yes	No	No	No	No	No	No	No	No	No	No	No	Dynamic AOI fixation distributions

No final included study analyzed interpersonal or intersubject gaze alignment.

S7. Cognitive, Behavioral, Evaluative, and Learning Outcomes

Supplementary Table S7A. Cognitive processing, load, performance, and gaze–cognition relationships.

RefID	Study	Synthesis subgroup	Cognitive constructs	Measures used	Cognitive-load finding	Performance / learning finding	Gaze–cognition relationship	Overall direction code	Critical caveat
LR0026	Morita et al. (2025)	4.7.3 Learning and information integration	Comprehension, retention, dual coding and cross-modal information integration	Ten-item post-reading tests, distraction task, post-survey; gaze AOI ratios and scanpaths	No formal cognitive-load scale or pupillometric load measure	Image-only augmentation improved scores slightly (+1.25%); text and image-summary conditions improved scores by 4.58% and 7.50%, respectively, and summary conditions outperformed baseline	Summary viewing and broader document coverage were interpreted as supporting integration; the relationship was primarily descriptive and varied by learner preference	Improved learning; cognitive load not measured	The baseline removed GenAI augmentation but retained AI-generated stories/questions; generated questions were not independently validated
LR0039	Fu et al. (2026)	4.7.4 Professional and creative tasks	Mental workload, cognitive effort, visual search, autonomic regulation and creativity	NASA-TLX, pupil measures, HRV, gaze trajectory length and CPSS creativity ratings	AI ideation reduced mental-demand ratings, but overall workload and most physiological differences were non-significant; development-phase load was lowest with curated images and intermediate with AIGC	AIGC supported novelty/elaboration in development and showed positive but not uniformly significant creativity trends in ideation	Shorter scanpaths and more balanced assistant/canvas viewing converged directionally with lower mental demand, but physiological evidence was mostly non-significant	Mixed/trade-off: selective load reduction and creativity benefit	Small specialized sample; hybrid within-/between-subject phases and session-level analysis complicate causal inference
LR0042	Cha et al. (2026)	4.7.2 Authenticity, synthetic content and detection	Visual processing, emotional arousal and prefrontal engagement	Fixation outcomes, pupil diameter, fNIRS HbO and socio-perceptual ratings	No formal cognitive-load scale; pupil and HbO were treated cautiously as non-specific arousal/engagement indicators	Selective age × image-type effects: adult 2D/3D characters received more fixations than real adults; middle-aged 3D stimuli elicited the largest pupil diameter; no universal AI-origin effect	Eye-tracking and fNIRS were interpreted jointly, but the effects were condition-specific rather than a consistent AI-processing signature	Condition-dependent; no general AI load effect	One image represented each age-by-type cell, so stimulus identity and condition were partly confounded
LR0123	Guan et al. (2025)	4.7.4 Professional and creative tasks	Cognitive evaluation, emotional evaluation, source attribution and sustained attention	Questionnaire mediation models plus fixation count, total fixation duration, TTFF and heatmaps	No formal cognitive-load measure	Cognitive evaluation mediated the relationship between semantic relevance and sustained attention; emotional evaluation was not a significant mediator	Gaze patterns changed after creator labels were shown, indicating that source belief and content features jointly shaped processing	Source- and content-dependent cognitive evaluation	Unlabeled viewing always preceded labeled viewing, creating possible order/carryover effects
LR0125	Abu-Rayyash & Lacruz (2025)	4.7.1 Reading and language processing	Subtitle-reading effort, attentional concentration and multimedia comprehension	Fixation count, mean duration, total subtitle time, gaze distribution, TTFF, K-coefficient and comprehension quiz	GPT-4o subtitles increased load on all principal gaze metrics and shifted viewing toward more focal subtitle processing	Comprehension outcomes favored professional subtitles, with higher accuracy in factual recall, cultural-reference recognition and narrative coherence	Greater subtitle inspection and a higher K-coefficient were interpreted as increased processing effort rather than greater positive engagement	Higher cognitive demand and lower comprehension for AI subtitles	Twenty-five of 107 participants were excluded for gaze quality; randomization details were incompletely reported

RefID	Study	Synthesis subgroup	Cognitive constructs	Measures used	Cognitive-load finding	Performance / learning finding	Gaze–cognition relationship	Overall direction code	Critical caveat
LR0144	Komninos et al. (2024)	4.7.1 Reading and language processing	Context processing, text-entry performance and phrase memorability	Fixation count/duration in prompt and response AOIs; WPM, errors and backspaces	No direct cognitive-load measure	In the eye-tracking study, the LLM context did not significantly alter core typing-speed/error measures, but it substantially increased attention to prompt and response regions	Attention to conversational context demonstrated processing of the generated material, but no formal gaze–performance association was established	More contextual processing; no clear performance cost/benefit	Eye-tracking sample was only 13; later non-eye-tracking studies in the paper should not be merged with the ET component
LR0152	Bozkir et al. (2024)	4.7.2 Authenticity, synthetic content and detection	Authenticity judgment, perceived realism, workload and visual search	Believability ratings, classification accuracy, NASA-TLX, fixation rate, saccade length and viewing/rating time	NASA-TLX showed no clear group or phase differences	Participants distinguished older PGGAN and real faces more successfully; performance declined for newer, more realistic StyleGAN/StyleGAN2 faces; priming improved some synthetic-face classification	Task phase and priming affected gaze more consistently than actual origin; no stable real-versus-synthetic gaze signature emerged	Detection difficulty increased with realism; load largely unchanged	Small technically familiar sample limits generalization
LR0166	Liu et al. (2026)	4.7.2 Authenticity, synthetic content and detection	Cultural authenticity, aesthetic processing, source recognition and symbolic interpretation	Eye tracking, cultural/aesthetic ratings, origin identification, technology-acceptance measures and interviews	No formal cognitive-load instrument	Human drawings showed stronger cultural fidelity, emotional/narrative depth and attention to diagnostic cultural details; AI images were often valued for fantasy, vividness and visual innovation	Longer fixation on culturally diagnostic regions accompanied higher human-content authenticity ratings; AI-proficient users detected AI images more accurately	Human advantage for culturally diagnostic processing; AI advantage for novelty/visual stimulation	Qualitative procedures and eye-tracking processing were underreported; stimuli were heavily curated
LR0167	Yfantidou et al. (2025)	4.7.4 Professional and creative tasks	Message processing, persuasive attention and behavioral intention	TTFF, dwell, fixation count, revisits and intention questionnaire	No formal cognitive-load measure	AI images delayed orienting to slogans and produced less slogan dwell, fewer fixations and fewer revisits than hand-drawn advertisements	Attention to the slogan was the proposed pathway to persuasive processing; anticipated-regret framing improved attention across image types	Lower message-focused processing for AI advertising images	AI, hand-drawn and stock images differed in low-level properties and complexity
LR0172	Yun & Kim (2026)	4.7.4 Professional and creative tasks	Visual competition, cognitive appraisal and environmental/aesthetic processing	Dwell, fixation count/duration, revisits, facial indicators and semantic-differential ratings	No formal cognitive-load scale; facial indicators were interpreted as implicit engagement/appraisal	Some AI variants increased façade exploration and positive ratings; the most complex composite diverted attention away from signage	Physiological/gaze engagement did not correlate consistently with self-reported aesthetics, revealing a complexity–aesthetic dissociation	Complexity-dependent benefit with attentional trade-offs	Eighteen of 48 participants were excluded; sample was predominantly young
LR0197	Yao et al. (2025)	4.7.4 Professional and creative tasks	Temporal, technical and cognitive effort during professional translation	Time/word, edit operations, pauses, source/target fixation measures, self-rated effort and retrospective interviews	AI assistance reduced source-text fixation effort but increased pause-based/technical effort; self-rated cognitive effort did not differ	AI assistance did not reduce total temporal effort and increased technical editing; no translation-quality outcome was reported	Gaze suggested less source consultation, whereas pauses and edits indicated added evaluation/coordination costs	Mixed: visual-effort reduction but technical/cognitive coordination cost	The qualitative component was weakly reported; GPT suggestions were embedded in a dynamic workflow
LR0202	Yuan et al. (2026)	4.7.3 Learning and information integration	Immediate learning, delayed retention, self-efficacy, cognitive load and multimedia integration	Knowledge tests, one-week retention, cognitive-load scales, EEG and gaze allocation	Mind-map support produced the lowest extraneous load and strongest germane processing; both summary groups reduced load relative to control	Both summary conditions improved posttest performance; the mind-map condition produced the largest gain and strongest delayed retention	Summary-AOI fixation was positively associated with learning outcomes, while extraneous/intrinsic load related negatively to performance	Improved learning and more efficient load, strongest for mind maps	Device model, calibration and fixation-filter details were not reported
LR0228	Wong et al. (2025)	4.7.2 Authenticity, synthetic content and detection	Authenticity assessment, diagnostic scrutiny and expert visual processing	Real/fake and diagnosis responses; first/last/longest/shortest fixations, saccades and saliency metrics	No formal cognitive-load measure	The paper collected authenticity and diagnostic judgments, but the central report emphasized gaze rather than full performance statistics	Initial and final gaze was similar; synthetic images produced slightly longer maximum fixations and more divergent prolonged-fixation maps, consistent with localized scrutiny	Subtle additional scrutiny of synthetic radiographs	Recruitment, missing-gaze flow and repeated participant/image dependence were incompletely addressed
LR0258	Gupta et al. (2020)	4.7.2 Authenticity, synthetic content and detection	Detection accuracy, perceptual difficulty and visual search	Real/fake decisions, gaze metrics and EEG classification	No formal cognitive-load scale	Accuracy was highest for real videos (.87), lower for easier fakes (.80) and lowest for difficult fakes (.61)	Deepfakes produced compact face-centered gaze; difficult fakes generated more re-fixation than easy fakes, linking gaze organization to detection difficulty	Detection worsened with fake quality; gaze became more focal	Missing gaze/EEG data and hierarchical participant/video dependence were not fully reported
LR0273	Cartella et al. (2024)	4.7.2 Authenticity, synthetic	Authenticity judgment and	Five-point authenticity ratings,	No formal cognitive-load measure	Participants generally differentiated original and edited images,	Edited images produced significantly lower saliency	AI-edited images elicited more	Participant characteristics, tracker model and

RefID	Study	Synthesis subgroup	Cognitive constructs	Measures used	Cognitive-load finding	Performance / learning finding	Gaze–cognition relationship	Overall direction code	Critical caveat
		content and detection	anomaly-focused search	fixation maps and saliency entropy		although the article emphasized distributional and gaze analyses rather than one summary accuracy statistic	entropy, indicating concentrated inspection of suspected anomalies	focal verification search	repeated-measures modeling were underreported
LR0280	Xu et al. (2026)	4.7.3 Learning and information integration	Cognitive load, retention, transfer and cross-area information integration	Cognitive-load questionnaire, retention/transfer tests, AOI gaze and behavioral sequence analysis	No significant cognitive-load difference between search conditions	GenAI search improved retention but did not significantly improve transfer	GenAI increased switching between search and simulation regions, suggesting active integration but not deeper context-dependent transfer	Retention benefit without load or transfer advantage	Gaze was analyzed at broad interface-AOI level rather than directly within generated answer text
LR0317	Cao et al. (2025)	4.7.3 Learning and information integration	Attention allocation and feedback-processing effort	Per-word fixation duration/count and AOI backtracking across four feedback dimensions	No independent cognitive-load scale; gaze metrics operationalized processing demand	No source-based difference in attention; completeness and grammar/vocabulary required more attention and backtracking than structure/coherence	Feedback dimension, rather than AI/human source, explained processing effort	No AI-versus-teacher processing difference	Only 10 participants; AI feedback was always left and teacher feedback always right
LR0321	Cosentino et al. (2025)	4.7.3 Learning and information integration	Task performance, cognitive load, global/local processing and verification	Task scores, pupil-derived load, information-processing index and AOI transitions	GenAI feedback produced lower pupil-derived cognitive load than human feedback	Task performance did not differ significantly between groups	GenAI learners cross-referenced correct options and the number line, whereas human-feedback learners focused more on provider/task/incorrect options	Lower load and different verification strategy; no performance gain	Sampling reporting was internally inconsistent (50-Hz gaze versus 250-Hz pupil statement)
LR0334	Zhou & Kawabata (2023)	4.7.2 Authenticity, synthetic content and detection	Aesthetic evaluation, perceived authorship and provenance recognition	Beauty/liking/valence/arousal ratings, origin classification and fixation outcomes	No formal cognitive-load measure	Actual authorship did not affect aesthetic ratings; human paintings were recognized more accurately (68%) than AI paintings (43%)	Paintings believed to be human-made received longer dwell regardless of actual origin, linking gaze more strongly to perceived than actual provenance	Perceived source, not actual source, shaped processing	Homogeneous non-expert student sample and representational landscape stimuli limit generalization
LR0386	Chen et al. (2025)	4.7.3 Learning and information integration	Help-seeking regulation, evaluation of assistance and revision-process organization	Multimodal process coding from gaze, trace and conversation data; process-mining transitions	No formal cognitive-load measure	No essay-quality or learning-performance outcome was reported as the principal endpoint	ChatGPT users followed more direct, nonlinear Asking→Processing pathways and spent less process time explicitly evaluating help than human-expert users	More iterative/direct AI help-seeking with less explicit evaluation	Gaze contributed to process coding rather than conventional fixation-effect estimates
LR0525	Ilyas et al. (2025)	4.7.1 Reading and language processing	Reading fluency, oculomotor effort and text difficulty	Fixation duration/rate, saccades, pupil size, regressions, reading speed and prepared comprehension questions	No standalone cognitive-load scale; pupil effects varied by author/style	Comprehension questions were administered, but comprehension results were not reported; reading speed was not significantly related to LIX	AI passages produced shorter but more frequent fixations and larger saccades; style/readability moderated the pattern	Distinct AI reading pattern; cognitive implication conditional on style	Final N=12; AI and human texts were not fully matched in length/readability
LR0558	Osińska et al. (2025)	4.7.2 Authenticity, synthetic content and detection	Visual recognition, artifact search and source classification	Binary origin judgments, AOI gaze, saccades and post-task explanations	No formal cognitive-load measure	Recognition varied substantially by image category; AI pets and real architecture were among the easiest, whereas art was among the most difficult	AI images elicited slightly longer fixations; experienced image/AI users used fewer, longer fixations and shorter saccades while searching for artifacts	Small AI scrutiny effect moderated by expertise and category	AI and real images were selected from different source pools and not fully matched
LR0560	Holmes et al. (2025)	4.7.2 Authenticity, synthetic content and detection	Detection accuracy and dynamic facial search	Binary detection, fixation/saccade measures and strategy reports	No formal cognitive-load measure	Mean eye-tracking-study detection accuracy was .65; gaze to eyes/mouth/body did not predict correct detection	Fixation duration/count and saccade count did not differ by authenticity or decision accuracy	Moderate detection performance with no diagnostic gaze signature	Convenience sample; exact deepfake generation models and eye-event parameters were not reported

Supplementary Table S7B. Visual, behavioral, evaluative, and learning engagement.

RefID	Study	Visual engagement	Behavioral engagement	Evaluative engagement	Learning engagement	Liking	Trust	Comprehension	Recognition / detection	Performance	Gaze–outcome relationship	Overall engagement interpretation
LR0026	Morita et al. (2025)	AOI fixation-duration ratios and scanpaths showed large individual differences in use	Hovering, reading, question answering and format preference	Interest/helpfulness and text-versus-image preference were collected	Comprehension and retention tests; learner preference	Not measured	Not measured	Measured and reported	Not measured	Measured and reported	Primarily descriptive/parallel; no single inferential gaze–engagement model	Engagement depended on learner preference and format rather than

RefID	Study	Visual engagement	Behavioral engagement	Evaluative engagement	Learning engagement	Liking	Trust	Comprehension	Recognition / detection	Performance	Gaze–outcome relationship	Overall engagement interpretation
		of generated summaries/images			aligned with best-performing format							AI augmentation alone
LR0039	Fu et al. (2026)	Fixations, gaze trajectories and pupil responses indexed involvement in ideation/development	Brainstorming, AI/human collaboration and concept production	Creative-product evaluation; no direct liking/trust scale	Not a learning study	Not measured	Not measured	Not measured	Not measured	Creativity/performance measured and reported	Multimodal convergence was directional; several physiological associations were non-significant	AI support appeared most engaging/effective during divergent ideation, with phase-specific trade-offs
LR0042	Cha et al. (2026)	Fixation duration/count and pupil diameter indexed attention to character faces	Viewing and repeated socio-perceptual rating	Interest, liking, attractiveness, persuasiveness and trustworthiness were measured	Not measured	Measured and reported	Measured and reported	Not measured	Not measured	Not measured	Gaze/fNIRS and ratings were interpreted jointly; questionnaire outcomes were mainly descriptive	Engagement was selective by depicted age and image type, not uniformly higher for AI
LR0123	Guan et al. (2025)	Fixation count, duration and TTFF operationalized sustained attention to logos	Selecting the most attention-catching logo and responding to source labels	Cognitive and emotional evaluation; creator-type/source judgments	Not measured	Not measured	Discussed through source credibility, not a standalone trust scale	Not measured	Not measured	Not measured	Study 1 tested mediation; Study 2 showed disclosure-dependent gaze redistribution	Creator disclosure and semantic relevance shaped engagement more than AI origin alone
LR0125	Abu-Rayyash & Lacruz (2025)	Subtitle/picture fixations, dwell, gaze proportion and K-coefficient	Viewing episode and completing quiz/survey	No direct liking/trust result in the focal analysis	Comprehension quiz favored professional subtitles	Not measured	Not measured	Measured and reported	Cultural-reference recognition measured within comprehension	Measured and reported	Gaze burden and comprehension converged against GPT-4o subtitles	More visual engagement with AI subtitles reflected effort and competition with imagery, not a positive engagement advantage
LR0144	Komninos et al. (2024)	Fixation duration/count to generated prompt and response regions	Mobile transcription, typing corrections and later memorability/engagement tasks	Task realism/emotional engagement were assessed in later non-eye-tracking work	Not measured in the eye-tracking component	Not measured	Not measured	Not measured in ET component	Not measured	Typing performance measured and reported	No direct gaze–performance association established	Generated context increased visual engagement without a clear typing-performance effect in the ET study
LR0152	Bozkir et al. (2024)	Viewing/rating time, fixation rate and saccade length	Real/fake classification and free-text cue reporting	Realism/believability and workload ratings	Not measured	Not measured	Not measured	Not measured	Measured and reported	Detection performance measured and reported	Gaze phase effects and performance were analyzed in parallel; priming improved some detection	Engagement was driven by evaluation task and realism, not a consistent AI-origin effect
LR0166	Liu et al. (2026)	Fixation to faces/cultural details plus heatmaps	Source identification, clicks, ratings and interviews	Aesthetic preference, emotional depth, cultural authenticity and technology acceptance	Not measured	Measured as aesthetic preference/appeal	Discussed/attitudinal, not a standalone trust metric	Not measured	Measured and reported	Source-identification performance reported	AI experience was statistically related to recognition accuracy; gaze/authenticity links were mainly descriptive	AI generated novelty and visual stimulation, whereas human work sustained culturally relevant engagement
LR0167	Yfantidou et al. (2025)	TTFF, slogan dwell, fixation count and revisits	Viewing advertisements and reporting screening-related intentions	Message framing and image-format evaluation; no direct liking/trust scale	Not measured	Not measured	Not measured	Not measured	Not measured	Behavioral intention collected	Mixed-effects models linked slogan/image factors to gaze; gaze–intention mediation was not established	AI images produced weaker message-focused visual engagement than hand-drawn images
LR0172	Yun & Kim (2026)	Dwell, fixations, revisits and facial indicators across façade/signage AOIs	Viewing façades and completing semantic-differential ratings	Aesthetic, natural, calming, vibrant, pleasant, restorative, comfort and harmony ratings	Not measured	Measured through aesthetic/pleasantness ratings	Not measured	Not measured	Not measured	Not measured	No consistent significant correlation between implicit/gaze engagement and self-reported aesthetics	Visually complex AI façades could increase exploration while reducing functional signage attention
LR0197	Yao et al. (2025)	Source/target fixation effort during post-editing	Editing operations, pauses and completion time	Self-rated effort and retrospective accounts; no liking/trust scale	Not measured	Not measured	Not measured	Not measured	Not measured	Temporal/technical task performance reported; quality not measured	Gaze, pauses, edits and self-report produced partly divergent effort profiles	Higher interaction with AI did not necessarily mean lower effort or better output
LR0202	Yuan et al. (2026)	Fixation-time allocation to video and generated summaries	Watching video and completing	Self-efficacy and cognitive-load judgments	Posttest and one-week retention measured	Not measured	Not measured	Measured and reported	Not measured	Learning performance measured and reported	Summary fixation was positively associated with test performance; load measures	Mind maps supported the strongest visual and learning engagement

RefID	Study	Visual engagement	Behavioral engagement	Evaluative engagement	Learning engagement	Liking	Trust	Comprehension	Recognition / detection	Performance	Gaze–outcome relationship	Overall engagement interpretation
			immediate/delayed tests								also related to performance	
LR0228	Wong et al. (2025)	First/last/longest/shortest fixation and saliency distributions	Authenticity classification and diagnosis	No liking/trust outcome	Not measured	Not measured	Not measured	Not measured	Collected; full classification result not central to report	Diagnosis/authenticity performance collected but incompletely reported	Gaze differences were reported without a full gaze–accuracy model	Synthetic radiographs generated subtle localized scrutiny rather than a broad engagement shift
LR0258	Gupta et al. (2020)	Fixations, re-fixations, entropy, scanpath length and heatmaps	Repeated real/fake decisions	No evaluative outcome beyond authenticity judgment	Not measured	Not measured	Not measured	Not measured	Measured and reported	Detection performance measured and reported	Detection difficulty and gaze organization were compared; difficult fakes combined lower accuracy with more reinspection	Deepfakes elicited focused face inspection but not superior detection
LR0273	Cartella et al. (2024)	Fixation/saliency concentration and entropy	Five-point authenticity judgments	Authenticity confidence/evaluation	Not measured	Not measured	Not measured	Not measured	Measured and reported	Authenticity judgment reported	Lower entropy accompanied edited-image inspection; exact gaze–accuracy association was not the main analysis	Focused attention to AI-edited anomalies did not guarantee easy detection
LR0280	Xu et al. (2026)	Dwell/fixations across video, simulation and search areas; cross-area saccades	Search queries, simulation use and behavioral sequences	Cognitive-load report; no liking/trust scale	Retention and transfer measured	Not measured	Not measured	Measured and reported	Not measured	Learning performance measured and reported	GenAI altered search/attention behavior; retention improved but transfer did not	Behavioral and visual engagement increased in exploration without a corresponding deep-transfer benefit
LR0317	Cao et al. (2025)	Fixations and backtracking on GenAI and teacher feedback	Reading feedback; no revision task outcome reported	No direct evaluative outcome	Not measured	Not measured	Not measured	Not measured	Not measured	No revision performance outcome	Source did not affect gaze; feedback dimension did	Visual engagement was equivalent by source and differentiated by feedback content
LR0321	Cosentino et al. (2025)	AOI time, transitions, processing index and pupillary activity	Embodied problem solving and response selection	No direct liking/trust/satisfaction scale	Task performance measured	Not measured	Discussed as possible skepticism; not directly measured	Measured and reported through task performance	Not measured	Task performance measured and reported	Gaze strategy and pupil-derived load differed, while task performance did not	GenAI changed learning engagement and verification strategy without improving scores
LR0334	Zhou & Kawabata (2023)	Painting dwell, fixation count/duration and heatmaps	Aesthetic rating and origin classification	Beauty, liking, valence, arousal, familiarity and concreteness	Not measured	Measured and reported	Not measured	Not measured	Measured and reported	Classification performance measured and reported	Perceived authorship predicted dwell more strongly than actual authorship	Visual engagement reflected source belief; explicit aesthetic ratings showed no actual-origin bias
LR0386	Chen et al. (2025)	ROI gaze stays embedded in multimodal help-seeking stages	Asking, diagnosing, evaluating, processing and revising behavior	No liking/trust/satisfaction scale	No learning/essay-quality endpoint reported	Not measured	Not measured	Not measured	Not measured	Process performance reported; product quality not reported	Gaze and trace data jointly defined process pathways rather than a gaze–outcome correlation	ChatGPT promoted direct iterative engagement but less explicit help evaluation
LR0525	Ilyas et al. (2025)	Fixation, saccade, pupil and regression measures during reading	Reading six passages and completing comprehension questions	No evaluative engagement scale	Comprehension collected but results not reported	Not measured	Not measured	Collected but not reported	Not measured	Reading speed reported; comprehension performance not reported	No formal gaze–comprehension association could be evaluated	AI text changed reading behavior, but evidence about understanding remained incomplete
LR0558	Osińska et al. (2025)	Fixations, saccades, AOI viewing and revisits	Real/AI classification and post-task explanations	Perceptual judgments and reasons; no liking/trust scale	Not measured	Not measured	Not measured	Not measured	Measured and reported	Recognition performance measured and reported	Expertise/age/sex patterns linked gaze strategies with recognition, but global origin effects were modest	Visual engagement with AI images was slightly longer and strongly category/expertise dependent
LR0560	Holmes et al. (2025)	Fixation and saccade allocation to dynamic facial/body AOIs	Deepfake detection and strategy reporting	No liking/trust/satisfaction scale	Not measured	Not measured	Not measured	Not measured	Measured and reported	Detection performance measured and reported	Gaze to eyes/mouth/body did not significantly predict correct classification	Participants engaged with diagnostic facial regions, but the pattern was not diagnostically effective

No included eye-tracking component formally measured continuation intention; satisfaction outcomes were absent or incompletely reported.

S8. Moderators and Boundary Conditions

Supplementary Table S8A. Participant, source, and additional moderators.

RefID	Study	Expertise	Participant age	AI familiarity	Disclosure / source belief	Other moderator(s)	Boundary-condition conclusion	Evidence location
LR0026	Morita et al. (2025)	Student/professional status described; expertise not tested	Age 21–31, not tested	LLM/IGM familiarity collected; not reported as moderator	No source-label manipulation	Learner preference (text vs image)	Preference strongly conditioned which GenAI format supported comprehension	Sections 4.1–5.3
LR0039	Fu et al. (2026)	Design-trained, mostly junior designers	Mean age 23.3; not tested	AI familiarity not a reported moderator	No disclosure manipulation	Assistance mode and workflow phase	AIGC benefits were stronger in divergent ideation than convergent development	Sections 3–7
LR0042	Cha et al. (2026)	No expertise moderation	Participant age narrow; depicted age experimentally manipulated	AI familiarity not tested	Participants knew real and AI images were included	Depicted age × image type	Effects were selective to specific age/type combinations	Sections 2.1–2.5
LR0123	Guan et al. (2025)	Brand/AI knowledge used in recruitment; not a central tested moderator	Age 19–43; not tested as key moderator	Understanding of AI required; prior attitudes not modeled	Creator type explicitly manipulated	Innovation × relevance × creator type	Source disclosure changed attention only in combination with logo characteristics	Sections 3–4
LR0125	Abu-Rayyash & Lacruz (2025)	English proficiency explicitly modeled	Age 18–50; not central moderator	AI familiarity not tested	Participants exposed to subtitle condition; source-label effects not manipulated	English proficiency	Advanced proficiency showed the largest GPT-related processing disruption	Sections 3–4
LR0144	Komninos et al. (2024)	English proficiency described; not a central interaction	Mean age 25; not tested	AI familiarity NR	Context source manipulated across paper; eye study used LLM vs baseline	Context presence, phrase memorability and presentation effect	Generated context changed attention without a simple typing-performance effect	Study 1 and later experiments
LR0152	Bozkir et al. (2024)	Technical/computer-science familiarity described	Mean age about 27; not tested	Deepfake familiarity collected/described	Primed versus unprimed groups	GAN generation method, priming and phase	Detection declined as synthetic faces became more realistic	Sections 3–4
LR0166	Liu et al. (2026)	Design/cultural expertise and AI proficiency central	Mostly 21–30; age not primary	AI-use intensity significantly related to identification accuracy	No label manipulation; source judgment required	AI expertise and cultural knowledge	Experienced users recognized AI more accurately and evaluated dissemination more cautiously	Sections 3.2–4.3
LR0167	Yfantidou et al. (2025)	No domain expertise manipulation	Age 40–65; age entered as covariate	AI familiarity not tested	No AI label manipulation	Slogan frame, image type, identity congruence, age and gender	Message framing remained effective, but AI imagery weakened slogan attention	Sections 2–3
LR0172	Yun & Kim (2026)	Professional network recruitment; expertise not tested	Mostly 18–25; age not modeled	AI familiarity NR	No disclosure manipulation	Pattern, material, color and composite complexity	Increasing complexity intensified façade attention but could reduce signage visibility	Sections 3–4
LR0197	Yao et al. (2025)	All were translation trainees; professional experience constrained	Age NR	Prior MT/post-editing experience described; AI familiarity not key	No source-label manipulation	Full versus light brief and post-editing mode	AI reduced some visual effort but did not eliminate temporal/technical costs	Sections 4–6
LR0202	Yuan et al. (2026)	Education students; prior knowledge measured	Mean age 21.4; not a moderator	AI familiarity NR	No source-label manipulation	Summary format and prior knowledge	Mind maps best supported integration, load and retention	Sections 4–6
LR0228	Wong et al. (2025)	Radiologist experience/specialty recorded but not adequately modeled	Age NR	AI familiarity NR	No source-label manipulation	Expertise level and diagnostic category (mostly descriptive)	Expert scrutiny differences were subtle and may depend on case/experience	Sections 3–4
LR0258	Gupta et al. (2020)	Mostly student non-experts	Young sample; age/sex not modeled	Prior deepfake expertise not central	No disclosure manipulation	Deepfake difficulty	Harder fakes reduced accuracy and increased reinspection	Sections 3.1–3.2
LR0273	Cartella et al. (2024)	Expertise NR	Age/sex NR	AI familiarity NR	No disclosure manipulation	Editing method	All edit methods reduced gaze entropy, but	Sections II–III

RefID	Study	Expertise	Participant age	AI familiarity	Disclosure / source belief	Other moderator(s)	Boundary-condition conclusion	Evidence location
							magnitude depended on editing approach	
LR0280	Xu et al. (2026)	University majors varied; expertise not a moderator	Age 17–24; not tested	Participants had limited GenAI-search experience	Tool condition visible; no label experiment	Search mode and prior knowledge	GenAI improved retention and cross-area exploration but not transfer	Sections 3–4
LR0317	Cao et al. (2025)	L2 undergraduates; no advanced writing training	Mean age 20.4; not tested	AI familiarity NR	Source was visibly separated by interface	Feedback dimension and source	Attention differences arose from feedback content, not source	Sections III–V
LR0321	Cosentino et al. (2025)	Children with no prior system/GenAI experience	Age 11–13; age range constrained	No prior GenAI experience; discussed as possible skepticism	Feedback source apparent	Feedback source and prior unfamiliarity	GenAI produced lower load but more verification-oriented gaze	Methods/Results
LR0334	Zhou & Kawabata (2023)	No art expertise	Mean age 21.3; not tested	AI familiarity NR	No source label before viewing; perceived authorship analyzed post hoc	Actual versus perceived authorship	Perceived human authorship—not actual origin—predicted longer viewing	Methods/Results
LR0386	Chen et al. (2025)	ESL university learners; baseline expertise not fully compared	Mean age 22.7; not tested	AI familiarity NR	Help source explicit	Help source and question type	AI promoted direct iterative action, whereas human help prompted more explicit evaluation	Sections 3–4
LR0525	Ilyas et al. (2025)	English proficiency varied; not formally modeled	Age 20–29; not tested	AI familiarity NR	Origin not the central disclosure manipulation	Author style and readability	Origin effects on gaze changed across imitated authors	Sections 2–3
LR0558	Osińska et al. (2025)	Digital-graphics and AI-image experience explicitly analyzed	Age cohort/generation analyzed	AI-image experience analyzed	No source-label manipulation	Expertise, age generation, sex and image category	Experienced/younger participants used more artifact-focused and efficient search strategies	Sections 4–7
LR0560	Holmes et al. (2025)	Non-expert sample by design	Mean age 26.3; not modeled	Deepfake familiarity/experience not a main analysis	No disclosure manipulation	AOI, authenticity and decision correctness	AOI preference was stable, but did not explain successful detection	Study 2

Supplementary Table S8B. Modality, task, quality, complexity, coherence, and matching.

RefID	Study	Modality	Task	Realism / quality	Complexity	Semantic coherence / relevance	Content matching	Boundary-condition conclusion	Content-matching code
LR0026	Morita et al. (2025)	Multimodal reading	Timed reading/retention	Generated image/text format and quality varied	Time pressure and information load varied by format	Story–summary/image alignment central to design	Partial: common interface, but different stories/augmentation layouts	Preference strongly conditioned which GenAI format supported comprehension	Partial
LR0039	Fu et al. (2026)	Conversational text + generated visual stimuli	Ideation versus concept development	Curated versus generated imagery; novelty/quality varied by phase	Task phase was the principal complexity boundary	Relevance of AI outputs to brief required interpretation/screening	Partial: same brief/time, but phase conditions differed	AIGC benefits were stronger in divergent ideation than convergent development	Partial
LR0042	Cha et al. (2026)	Static real/2D/3D faces	Free viewing and socio-perceptual rating	Real, 2D and 3D representation/realism manipulated	Face age and representational type created factorial complexity	Not a primary moderator	Strong low-level matching; one unique stimulus per cell remains confounded	Effects were selective to specific age/type combinations	Partial
LR0123	Guan et al. (2025)	Static logos	Free viewing, source disclosure and selection	Innovation and aesthetic quality experimentally selected	Innovation × semantic relevance manipulated	Core experimental moderator: high/low semantic relevance	Strong visual standardization and expert selection; creator labels changed display	Source disclosure changed attention only in combination with logo characteristics	Partial
LR0125	Abu-Rayyash & Lacruz (2025)	Textual subtitles over video	Audiovisual comprehension	Professional versus unedited GPT subtitle quality	Cultural references, line segmentation and reading demand increased complexity	Translation coherence and cultural adequacy were important	Strong: same episode/timing/technical subtitle constraints	Advanced proficiency showed the largest GPT-related processing disruption	Partial
LR0144	Komninos et al. (2024)	Generated dialogue around transcription	Mobile text entry	Prompt–response relevance and memorability controlled/selected	Gradual presentation and phrase memorability tested across paper	Prompt/response contextual relevance central	Partial: same task/display, but phrase sets differed by condition	Generated context changed attention without a simple typing-performance effect	Partial
LR0152	Bozkir et al. (2024)	Static real/GAN faces	Free viewing, rating and classification	GAN generation sophistication/realism was a major boundary	Task phase and generation method altered difficulty	Not primary	Good size/gender/pose control; residual model-specific artifacts remained	Detection declined as synthetic faces became more realistic	Partial

RefID	Study	Modality	Task	Realism / quality	Complexity	Semantic coherence / relevance	Content matching	Boundary-condition conclusion	Content-matching code
LR0166	Liu et al. (2026)	Static cultural illustrations	Free viewing, source judgment and cultural evaluation	Cultural fidelity versus fantasy/visual innovation	Cultural-symbol density and narrative complexity mattered	Text–image/cultural-semantic fidelity central	Strong shared script/prompts; AI and human production processes remained different	Experienced users recognized AI more accurately and evaluated dissemination more cautiously	Partial
LR0167	Yfantidou et al. (2025)	Static health advertisements	Persuasive viewing and intention	AI/hand-drawn/stock format affected perceived quality and realism	Visual complexity and slogan framing influenced search	Slogan framing and identity congruence central	Partial/weak: unavoidable low-level differences across image formats	Message framing remained effective, but AI imagery weakened slogan attention	Partial
LR0172	Yun & Kim (2026)	Static architectural façades	Aesthetic appraisal	Color, material, pattern and biophilic design quality varied	Design complexity was central	Harmony with storefront/signage central	Strong common base image; generated variants differed by intended design attributes	Increasing complexity intensified façade attention but could reduce signage visibility	Partial
LR0197	Yao et al. (2025)	Interactive text/translation	Full versus light post-editing	GPT suggestion usefulness/accuracy varied dynamically	Post-editing brief was the main task-complexity moderator	Text relevance and adequacy evaluated during editing	Strong Latin-square text/brief allocation; dynamic suggestions inherently varied	AI reduced some visual effort but did not eliminate temporal/technical costs	Partial
LR0202	Yuan et al. (2026)	Generated text and mind-map summaries	Video learning	Summary format and expert-validated quality	Text versus mind-map structural complexity	Information equivalence and summary coherence expert-checked	Strong content equivalence and same display area	Mind maps best supported integration, load and retention	Partial
LR0228	Wong et al. (2025)	Synthetic/real medical images	Authenticity and diagnosis	Clinical realism of generated X-rays central	Diagnostic category and image complexity varied	Report–image semantic consistency built into generation	Strong paired report conditioning; repeated image/reader effects incompletely modeled	Expert scrutiny differences were subtle and may depend on case/experience	Partial
LR0258	Gupta et al. (2020)	Dynamic real/deepfake video	Detection	Easy versus difficult deepfake quality explicitly coded	Video difficulty was central	Narrative/semantic coherence not a main manipulation	Partial: real/fake counts and viewing frequencies were not fully balanced	Harder fakes reduced accuracy and increased reinspection	Partial
LR0273	Cartella et al. (2024)	Static original/AI-edited images	Authenticity rating	Three editing methods varied artifact type/realism	Edit method and scene content varied complexity	Instruction-guided semantic consistency varied	Strong paired original/edited structure; edits differed by method	All edit methods reduced gaze entropy, but magnitude depended on editing approach	Partial
LR0280	Xu et al. (2026)	Interactive GenAI search	Video learning, simulation and search	Generated-answer quality depended on RAG and user query	Concurrent video/simulation/search increased task complexity	Contextual relevance via retrieval augmentation	Strong common lecture/task; search outputs necessarily differed dynamically	GenAI improved retention and cross-area exploration but not transfer	Partial
LR0317	Cao et al. (2025)	Text feedback	Feedback reading	Feedback quality pedagogically constrained	Feedback dimension drove processing complexity	Completeness/coherence/grammar relevance central	Weak/partial: AI left/teacher right and content/length varied	Attention differences arose from feedback content, not source	Partial
LR0321	Cosentino et al. (2025)	Text feedback in embodied task	Embodied mathematics learning	Feedback structure/quality co-designed	Embodied, multisensory problem solving increased complexity	Feedback–task relevance central	Strong same problem context; human and AI delivery differed	GenAI produced lower load but more verification-oriented gaze	Partial
LR0334	Zhou & Kawabata (2023)	Static paintings	Free viewing, aesthetics and source classification	Representational landscape realism/style	Painting/image characteristics modeled	Perceived stylistic coherence influenced source belief	Moderate/strong matching and painting random effects; origins came from different production sources	Perceived human authorship—not actual origin—predicted longer viewing	Partial
LR0386	Chen et al. (2025)	Interactive help responses	Essay writing and revision	Response usefulness depended on dynamic conversation	Question type and help-seeking stage varied complexity	Context-rich prompt/rubric promoted relevance	Strong common task/platform; help content differed by agent	AI promoted direct iterative action, whereas human help prompted more explicit evaluation	Partial
LR0525	Ilyas et al. (2025)	Literary text	Reading/comprehension	Imitated author/style and text readability differed	LIX/readability and author style central	Stylistic coherence with the imitated author central	Partial: typography/layout controlled, length/readability imperfectly matched	Origin effects on gaze changed across imitated authors	Partial
LR0558	Osińska et al. (2025)	Static multi-category images	Real/AI classification	Image category and generation realism central	Category complexity varied widely	Semantic plausibility varied by category	Weak/partial: different source pools and subjective ‘best image’ selection	Experienced/younger participants used more artifact-focused and efficient search strategies	Partial
LR0560	Holmes et al. (2025)	Dynamic authentic/deepfake video	Detection	Deepfake quality not stratified in the eye study	Dynamic movement and single-view constraint shaped difficulty	Narrative/semantic coherence not separately tested	Strong matched authentic/deepfake video sets	AOI preference was stable, but did not explain successful detection	Partial

Content-matching codes summarize study-level matching quality and should be read with the narrative field.

S9. MMAT Appraisal

Supplementary Table S9A. Compact MMAT profile and narrative appraisal.

RefID	Study	MMAT design	Applicable criterion ratings	Narrative appraisal
LR0026	Morita et al. (2025)	Quantitative non-randomized	S1: Yes; S2: Yes; 3.1: No; 3.2: Can't tell; 3.3: Yes; 3.4: No; 3.5: Yes	Limited representativeness and residual story/layout confounding; outcome measures partly unvalidated.
LR0039	Fu et al. (2026)	Quantitative non-randomized	S1: Yes; S2: Yes; 3.1: No; 3.2: Yes; 3.3: Yes; 3.4: No; 3.5: Yes	Rich multimodal methods, but small specialist sample and inconsistent treatment of repeated/session-level observations.
LR0042	Cha et al. (2026)	Quantitative non-randomized	S1: Yes; S2: Yes; 3.1: No; 3.2: Yes; 3.3: Yes; 3.4: No; 3.5: Yes	Detailed measurement and low-level matching; narrow sample and one-stimulus-per-cell confounding.
LR0123	Guan et al. (2025)	Quantitative non-randomized	S1: Yes; S2: Yes; 3.1: No; 3.2: Yes; 3.3: Yes; 3.4: No; 3.5: Yes	Good material standardization; fixed unlabeled→labeled sequence creates order/carryover risk.
LR0125	Abu-Rayyash & Lacruz (2025)	Quantitative randomized trial	S1: Yes; S2: Yes; 2.1: Can't tell; 2.2: Can't tell; 2.3: No; 2.4: Yes; 2.5: Yes	Direct randomized comparison; randomization reporting incomplete and about 23% of participants excluded.
LR0144	Komninos et al. (2024)	Quantitative non-randomized	S1: Yes; S2: Yes; 3.1: No; 3.2: Yes; 3.3: Yes; 3.4: Can't tell; 3.5: Yes	Appropriate task and gaze outcomes; very small convenience sample and residual phrase/order confounding.
LR0152	Bozkir et al. (2024)	Quantitative non-randomized	S1: Yes; S2: Yes; 3.1: No; 3.2: Yes; 3.3: Yes; 3.4: Yes; 3.5: Yes	Coherent controlled design and confounder handling; small technically familiar sample.
LR0166	Liu et al. (2026)	Mixed methods	S1: Yes; S2: Yes; 1.1: Yes; 1.2: Can't tell; 1.3: No; 1.4: No; 1.5: Can't tell; 3.1: Yes; 3.2: Can't tell; 3.3: Yes; 3.4: No; 3.5: Yes; 5.1: Yes; 5.2: No; 5.3: No; 5.4: Can't tell; 5.5: No	Relevant mixed-method rationale, but qualitative analysis/integration and ET reporting are weak.
LR0167	Yfantidou et al. (2025)	Quantitative non-randomized	S1: Yes; S2: Yes; 3.1: Yes; 3.2: Yes; 3.3: Can't tell; 3.4: Yes; 3.5: Yes	Strong sampling relevance and ET quality procedures; completeness/attrition pathway incompletely reported.
LR0172	Yun & Kim (2026)	Quantitative non-randomized	S1: Yes; S2: Yes; 3.1: No; 3.2: Yes; 3.3: No; 3.4: Yes; 3.5: Yes	Controlled stimulus variants and multimodal outcomes; high data-exclusion rate and young sample.
LR0197	Yao et al. (2025)	Mixed methods	S1: Yes; S2: Yes; 1.1: Yes; 1.2: Can't tell; 1.3: No; 1.4: No; 1.5: Can't tell; 3.1: Yes; 3.2: Yes; 3.3: Yes; 3.4: Yes; 3.5: Yes; 5.1: Yes; 5.2: Yes; 5.3: Yes; 5.4: Yes; 5.5: No	Strong quantitative Latin-square/mixed-model component; qualitative component weakly documented.
LR0202	Yuan et al. (2026)	Quantitative randomized trial	S1: Yes; S2: Yes; 2.1: Yes; 2.2: Yes; 2.3: Yes; 2.4: No; 2.5: Yes	Strong randomization/baseline/completeness; blinding impossible or unreported.
LR0228	Wong et al. (2025)	Quantitative non-randomized	S1: Yes; S2: Yes; 3.1: Can't tell; 3.2: Yes; 3.3: Can't tell; 3.4: No; 3.5: Yes	Clinically relevant expert sample and measures; sampling/completeness and repeated-observation confounding unclear.
LR0258	Gupta et al. (2020)	Quantitative non-randomized	S1: Yes; S2: Yes; 3.1: No; 3.2: Yes; 3.3: Can't tell; 3.4: No; 3.5: Yes	Valuable multimodal database; convenience sample, unclear missing data and hierarchical analysis concerns.
LR0273	Cartella et al. (2024)	Quantitative non-randomized	S1: Yes; S2: Yes; 3.1: Can't tell; 3.2: Yes; 3.3: Can't tell; 3.4: No; 3.5: Yes	Appropriate paired exposure; participant reporting and repeated-measures analysis weak.
LR0280	Xu et al. (2026)	Quantitative randomized trial	S1: Yes; S2: Yes; 2.1: Can't tell; 2.2: Yes; 2.3: Yes; 2.4: No; 2.5: Yes	Good comparative implementation and completeness; allocation method and blinding underreported.
LR0317	Cao et al. (2025)	Quantitative non-randomized	S1: Yes; S2: Yes; 3.1: No; 3.2: Yes; 3.3: Yes; 3.4: No; 3.5: Yes	Clear direct feedback comparison; N=10 and serious left/right position/content confounding.
LR0321	Cosentino et al. (2025)	Quantitative randomized trial	S1: Yes; S2: Yes; 2.1: Can't tell; 2.2: Can't tell; 2.3: Yes; 2.4: No; 2.5: Yes	Relevant randomized school study; randomization details, baseline equivalence and blinding insufficient.
LR0334	Zhou & Kawabata (2023)	Quantitative non-randomized	S1: Yes; S2: Yes; 3.1: No; 3.2: Yes; 3.3: Yes; 3.4: Yes; 3.5: Yes	Strong modeling and complete data; homogeneous non-expert sample.
LR0386	Chen et al. (2025)	Quantitative randomized trial	S1: Yes; S2: Yes; 2.1: Can't tell; 2.2: Can't tell; 2.3: Yes; 2.4: No; 2.5: Yes	Rich multimodal process analysis; randomization/baseline/blinding underreported.
LR0525	Ilyas et al. (2025)	Quantitative non-randomized	S1: Yes; S2: Yes; 3.1: No; 3.2: Yes; 3.3: Yes; 3.4: Yes; 3.5: Yes	Careful counterbalancing and preprocessing; final N=12 and imperfect text matching.
LR0558	Osińska et al. (2025)	Quantitative non-randomized	S1: Yes; S2: Yes; 3.1: No; 3.2: Yes; 3.3: Yes; 3.4: No; 3.5: Yes	Complete direct comparison; convenience sampling and weak AI/real stimulus matching.
LR0560	Holmes et al. (2025)	Quantitative non-randomized	S1: Yes; S2: Yes; 3.1: No; 3.2: Yes; 3.3: Yes; 3.4: Yes; 3.5: Yes	Well-controlled dynamic AOI study; small nonrepresentative convenience sample.

No summed MMAT percentage or exclusion threshold was applied. All 23 studies passed S1 and S2.

Supplementary Table S9B. Full MMAT criterion-level audit trail.

RefID	MMAT design	Criterion	Rating	Evidence / rationale	Source location
LR0026	Quantitative non-randomized	S1 — Clear research questions/objectives	Yes	The report states explicit aims, hypotheses or objectives concerning comparative human responses to the studied AI-generated content in "GenAIReading: Augmenting Human Cognition with Interactive Digital Textbooks Using Large Language Models and Image Generation Models".	Abstract; Introduction / stated aims
LR0026	Quantitative non-randomized	S2 — Collected data address the questions/objectives	Yes	The collected participant data include eye-tracking/gaze outcomes and the relevant behavioral, cognitive, evaluative or physiological measures needed to address the stated comparative objective.	Methods; Results
LR0026	Quantitative non-randomized	3.1 — Participants representative of target population	No	The 24 participants were young university students or professionals based in Germany and appear to be a convenience sample; this does not represent the broader population of digital-textbook learners.	Participants
LR0026	Quantitative non-randomized	3.2 — Measurements appropriate	Can't tell	Eye tracking, AOIs and the fixation algorithm are described well, but the stories and comprehension questions were AI-generated and no independent validation of question difficulty or construct validity is reported.	Eye-Tracking Data Processing; Experiment Setup
LR0026	Quantitative non-randomized	3.3 — Complete outcome data	Yes	All 24 recruited participants appear to have completed and contributed data; no attrition or missing-outcome exclusions are reported.	Participants; Results
LR0026	Quantitative non-randomized	3.4 — Confounders accounted for	No	The baseline story was fixed, layouts and information load differed by condition, and the generated stories/questions may differ in difficulty; these factors were not adequately modeled or controlled in the outcome analysis.	Order of Reading Conditions; Reading Time; Results
LR0026	Quantitative non-randomized	3.5 — Intervention/exposure occurred as intended	Yes	The web application imposed the four predefined conditions, fixed timing and controlled laboratory procedure, indicating that exposure occurred as intended.	Web Application; Experiment Procedure
LR0039	Quantitative non-randomized	S1 — Clear research questions/objectives	Yes	The report states explicit aims, hypotheses or objectives concerning comparative human responses to the studied AI-generated content in "Evaluating the Impact of AIGC-Supported Design Ideation on Designers' Cognitive Load and Creativity".	Abstract; Introduction / stated aims
LR0039	Quantitative non-randomized	S2 — Collected data address the questions/objectives	Yes	The collected participant data include eye-tracking/gaze outcomes and the relevant behavioral, cognitive, evaluative or physiological measures needed to address the stated comparative objective.	Methods; Results
LR0039	Quantitative non-randomized	3.1 — Participants representative of target population	No	Only 18 design-trained participants, mostly junior designers, were studied in a controlled laboratory setting; the sample is too narrow for the wider designer population.	Participants; Limitations
LR0039	Quantitative non-randomized	3.2 — Measurements appropriate	Yes	NASA-TLX, CPSS, Tobii Pro Fusion eye tracking, pupil metrics and HRV are established measures and are operationalized in detail.	Measures; Physiological and Behavioral Measures
LR0039	Quantitative non-randomized	3.3 — Complete outcome data	Yes	The report states that 36 sessions yielded high-quality datasets and that no appreciable data loss occurred after preprocessing.	Results & Analysis
LR0039	Quantitative non-randomized	3.4 — Confounders accounted for	No	The within-subject ideation phase was analyzed with independent-samples tests, and the development assignment did not stratify prior brainstorming source; session-level dependence and carryover/confounding are not fully addressed.	Experimental Design; Results
LR0039	Quantitative non-randomized	3.5 — Intervention/exposure occurred as intended	Yes	Condition-specific interfaces and tasks were prespecified, recorded continuously and delivered under consistent laboratory conditions.	Experimental Procedure; Apparatus
LR0042	Quantitative non-randomized	S1 — Clear research questions/objectives	Yes	The report states explicit aims, hypotheses or objectives concerning comparative human responses to the studied AI-generated content in "Impact of AI-Generated Characters on Visual Attention and Prefrontal Cognitive Responses Across Age and Image Type Conditions: Using fNIRS and Eye-Tracking".	Abstract; Introduction / stated aims
LR0042	Quantitative non-randomized	S2 — Collected data address the questions/objectives	Yes	The collected participant data include eye-tracking/gaze outcomes and the relevant behavioral, cognitive, evaluative or physiological measures needed to address the stated comparative objective.	Methods; Results

RefID	MMAT design	Criterion	Rating	Evidence / rationale	Source location
LR0042	Quantitative non-randomized	3.1 — Participants representative of target population	No	The sample comprised 24 right-handed university students aged 21–26; this homogeneous group is not representative of general users of AI-generated characters.	Participants; Limitations
LR0042	Quantitative non-randomized	3.2 — Measurements appropriate	Yes	The study used a 1,200-Hz Tobii Pro Spectrum, concurrent fNIRS, defined eye outcomes, repeated-measures models, calibration/quality checks and detailed preprocessing.	Data Acquisition; Statistical Analysis
LR0042	Quantitative non-randomized	3.3 — Complete outcome data	Yes	All 24 eligible participants completed the experiment and are represented in the analyses; exclusions were defined prospectively.	Participants; Results
LR0042	Quantitative non-randomized	3.4 — Confounders accounted for	No	Only one image represented each age-by-image-type combination, so identity and unique stimulus characteristics remain confounded with condition despite luminance/contrast matching and expert selection.	Visual Stimuli; Experimental Design
LR0042	Quantitative non-randomized	3.5 — Intervention/exposure occurred as intended	Yes	All participants viewed all 18 stimuli in randomized order using a fixed timing protocol.	Experimental Design
LR0123	Quantitative non-randomized	S1 — Clear research questions/objectives	Yes	The report states explicit aims, hypotheses or objectives concerning comparative human responses to the studied AI-generated content in "Algorithmic Esthetics Meets Human Intuition: The Synergy of Innovation and Relevance Shapes Sustained Attention".	Abstract; Introduction / stated aims
LR0123	Quantitative non-randomized	S2 — Collected data address the questions/objectives	Yes	The collected participant data include eye-tracking/gaze outcomes and the relevant behavioral, cognitive, evaluative or physiological measures needed to address the stated comparative objective.	Methods; Results
LR0123	Quantitative non-randomized	3.1 — Participants representative of target population	No	Participants were recruited offline from two universities in China and South Korea; the convenience regional samples do not represent broader consumers.	Study 1 and Study 2 Participants
LR0123	Quantitative non-randomized	3.2 — Measurements appropriate	Yes	Logo materials were expert screened and standardized; Study 2 used Tobii Pro Nano, nine-point calibration and appropriate fixation, duration and TTFF outcomes.	Experimental Materials; Apparatus; Procedure
LR0123	Quantitative non-randomized	3.3 — Complete outcome data	Yes	Study 2 reports 60 recruited and 54 valid eye-tracking samples, with six exclusions explicitly attributed to low sampling rates.	Study 2 Procedure
LR0123	Quantitative non-randomized	3.4 — Confounders accounted for	No	The creator-unlabeled exposure always preceded the labeled exposure, and creator labels added visual information; order, learning and display-change effects were not counterbalanced.	Study 2 Materials and Procedure
LR0123	Quantitative non-randomized	3.5 — Intervention/exposure occurred as intended	Yes	The two-stage exposure, presentation times and creator labels were delivered using a programmed Tobii Pro Lab protocol.	Study 2 Procedure
LR0125	Quantitative randomized controlled trial	S1 — Clear research questions/objectives	Yes	The report states explicit aims, hypotheses or objectives concerning comparative human responses to the studied AI-generated content in "Through the Eyes of the Viewer: The Cognitive Load of LLM-Generated vs. Professional Arabic Subtitles".	Abstract; Introduction / stated aims
LR0125	Quantitative randomized controlled trial	S2 — Collected data address the questions/objectives	Yes	The collected participant data include eye-tracking/gaze outcomes and the relevant behavioral, cognitive, evaluative or physiological measures needed to address the stated comparative objective.	Methods; Results
LR0125	Quantitative randomized controlled trial	2.1 — Randomization appropriately performed	Can't tell	Participants were described as randomly assigned and proficiency stratification was used, but the random-sequence generation and allocation-concealment procedures are not reported.	Design / Assignment
LR0125	Quantitative randomized controlled trial	2.2 — Groups comparable at baseline	Can't tell	The final groups were similar in size, but no condition-specific baseline table tests age, gender and English-proficiency comparability after randomization.	Participants; Baseline Data
LR0125	Quantitative randomized controlled trial	2.3 — Complete outcome data	No	Twenty-five of 107 participants (approximately 23.4%) were excluded for gaze-quality reasons and no intention-to-treat analysis is reported.	Participants / Data Quality
LR0125	Quantitative randomized controlled trial	2.4 — Outcome assessors blinded	Yes	The primary eye-tracking outcomes were recorded and computed automatically; assessor judgment was unlikely to vary by subtitle condition.	Eye-Tracking Protocol
LR0125	Quantitative randomized controlled trial	2.5 — Participants adhered to assigned intervention	Yes	Participants watched the same episode with the subtitle condition to which they were assigned; crossover/non-adherence is not reported.	Procedure
LR0144	Quantitative non-randomized	S1 — Clear research questions/objectives	Yes	The report states explicit aims, hypotheses or objectives concerning comparative human responses to the studied	Abstract; Introduction / stated aims

RefID	MMAT design	Criterion	Rating	Evidence / rationale	Source location
				AI-generated content in “An LLM-Driven Transcription Task for Mobile Text Entry Studies”.	
LR0144	Quantitative non-randomized	S2 — Collected data address the questions/objectives	Yes	The collected participant data include eye-tracking/gaze outcomes and the relevant behavioral, cognitive, evaluative or physiological measures needed to address the stated comparative objective.	Methods; Results
LR0144	Quantitative non-randomized	3.1 — Participants representative of target population	No	The eye-tracking experiment included only 13 participants, predominantly male and highly proficient in English; it is not representative of mobile text-entry users.	Study 1 Participants
LR0144	Quantitative non-randomized	3.2 — Measurements appropriate	Yes	Tobii T120 eye tracking and fixation count/duration within prompt and response AOIs appropriately operationalize visual attention to the LLM context.	Study 1 Apparatus and Measures
LR0144	Quantitative non-randomized	3.3 — Complete outcome data	Yes	No participant attrition or missing outcome data are reported for the 13-person eye-tracking study.	Study 1 Results
LR0144	Quantitative non-randomized	3.4 — Confounders accounted for	Can’t tell	Condition order was randomized, but different phrase sets were sampled for different conditions and phrase-level dependence/carryover were not clearly modeled; the residual impact cannot be determined.	Study 1 Design and Analysis
LR0144	Quantitative non-randomized	3.5 — Intervention/exposure occurred as intended	Yes	The programmed transcription task displayed the assigned LLM or baseline context as planned.	Study 1 Procedure
LR0152	Quantitative non-randomized	S1 — Clear research questions/objectives	Yes	The report states explicit aims, hypotheses or objectives concerning comparative human responses to the studied AI-generated content in “Can You Tell Real from Fake Face Images? Perception of Computer-Generated Faces by Humans”.	Abstract; Introduction / stated aims
LR0152	Quantitative non-randomized	S2 — Collected data address the questions/objectives	Yes	The collected participant data include eye-tracking/gaze outcomes and the relevant behavioral, cognitive, evaluative or physiological measures needed to address the stated comparative objective.	Methods; Results
LR0152	Quantitative non-randomized	3.1 — Participants representative of target population	No	The sample included 22 participants with substantial computer-science or technical familiarity and is not representative of the general public.	Participants
LR0152	Quantitative non-randomized	3.2 — Measurements appropriate	Yes	A 600-Hz Tobii system, real/GAN stimulus sets, believability judgments, fixation/saccade outcomes and NASA-TLX were appropriate to the research question.	Experiment and Measures
LR0152	Quantitative non-randomized	3.3 — Complete outcome data	Yes	All recruited participants appear in the analysis and no missing-outcome concern is reported.	Participants; Results
LR0152	Quantitative non-randomized	3.4 — Confounders accounted for	Yes	Stimuli were gender balanced and randomized; priming groups were randomly formed; all participants viewed real and multiple GAN sources under a common protocol.	Design; Stimuli; Procedure
LR0152	Quantitative non-randomized	3.5 — Intervention/exposure occurred as intended	Yes	Real and synthetic face exposures were delivered under the planned viewing and rating phases.	Procedure
LR0166	Mixed methods	S1 — Clear research questions/objectives	Yes	The report states explicit aims, hypotheses or objectives concerning comparative human responses to the studied AI-generated content in “Can AI See the Unseen? Measuring the Perception Gap for Tibetan Cultural Symbols in AI-Generated Art”.	Abstract; Introduction / stated aims
LR0166	Mixed methods	S2 — Collected data address the questions/objectives	Yes	The collected participant data include eye-tracking/gaze outcomes and the relevant behavioral, cognitive, evaluative or physiological measures needed to address the stated comparative objective.	Methods; Results
LR0166	Mixed methods	1.1 — Qualitative approach appropriate	Yes	Interviews are an appropriate approach for exploring cultural authenticity, aesthetic interpretation and participant reasoning that cannot be captured by gaze measures alone.	Methods / Mixed-Method Framework
LR0166	Mixed methods	1.2 — Qualitative data collection adequate	Can’t tell	The report mentions in-depth interviews but does not adequately describe the interview guide, interviewer, sampling, recording or transcription procedures.	Interview Methods
LR0166	Mixed methods	1.3 — Findings adequately derived from data	No	No transparent qualitative coding procedure, theme-development process or audit trail is reported.	Qualitative Analysis
LR0166	Mixed methods	1.4 — Interpretation sufficiently substantiated by data	No	Interpretive claims are not substantiated with participant quotations or sufficiently detailed qualitative evidence.	Results / Interviews
LR0166	Mixed methods	1.5 — Coherence across qualitative sources, collection, analysis and interpretation	Can’t tell	Because collection and analysis procedures are underreported, coherence between interview data, analysis and interpretation cannot be established.	Methods and Results

RefID	MMAT design	Criterion	Rating	Evidence / rationale	Source location
LR0166	Mixed methods	3.1 — Participants representative of target population	Yes	The purposive sample included designers, computer-science participants and educators with relevant AI/cultural expertise, consistent with the study's stated perception-gap target.	Participants
LR0166	Mixed methods	3.2 — Measurements appropriate	Can't tell	Questionnaire and task concepts are relevant, but the eye-tracker model, sampling rate, calibration, fixation algorithm and several outcome definitions are insufficiently reported.	Eye-Tracking Method
LR0166	Mixed methods	3.3 — Complete outcome data	Yes	All 38 participants appear to have completed the tasks and been included in reported analyses.	Participants; Results
LR0166	Mixed methods	3.4 — Confounders accounted for	No	AI outputs were heavily curated, human illustrations were produced by graduate illustrators, and equivalence in low-level visual properties and production conditions was not demonstrated.	Stimulus Development
LR0166	Mixed methods	3.5 — Intervention/exposure occurred as intended	Yes	Participants were exposed to the planned AI-generated and human-drawn illustrations across the specified tasks.	Procedure
LR0166	Mixed methods	5.1 — Adequate rationale for mixed methods	Yes	The paper explicitly combines eye tracking, questionnaires and interviews to capture both measurable attention and subjective cultural/aesthetic interpretation.	Study Design
LR0166	Mixed methods	5.2 — Components effectively integrated	No	The report does not describe a formal integration procedure linking qualitative and quantitative datasets at collection, analysis or joint display stages.	Mixed-Method Integration
LR0166	Mixed methods	5.3 — Integrated outputs adequately interpreted	No	No clear meta-inferences are derived from explicit integration of qualitative and quantitative results.	Discussion
LR0166	Mixed methods	5.4 — Divergences/inconsistencies adequately addressed	Can't tell	Potential convergence or divergence between interview, questionnaire and eye-tracking findings is not systematically reported.	Results / Discussion
LR0166	Mixed methods	5.5 — Each component adheres to its methodological quality criteria	No	The qualitative component fails several MMAT qualitative criteria, so the overall mixed-method quality cannot exceed that weaker component.	Component Appraisals
LR0167	Quantitative non-randomized	S1 — Clear research questions/objectives	Yes	The report states explicit aims, hypotheses or objectives concerning comparative human responses to the studied AI-generated content in "Mapping the Gaze: Comparing the Effectiveness of Bowel-Cancer Screening Advertisements".	Abstract; Introduction / stated aims
LR0167	Quantitative non-randomized	S2 — Collected data address the questions/objectives	Yes	The collected participant data include eye-tracking/gaze outcomes and the relevant behavioral, cognitive, evaluative or physiological measures needed to address the stated comparative objective.	Methods; Results
LR0167	Quantitative non-randomized	3.1 — Participants representative of target population	Yes	Quota/purposive recruitment targeted UK adults aged 40–65—the intended bowel-screening audience—and included White and South Asian participants, making the sample relevant to the stated target.	Participants and Recruitment
LR0167	Quantitative non-randomized	3.2 — Measurements appropriate	Yes	The study reports RealEyes quality thresholds, calibration, I-VT fixation processing, AOIs and established attention outcomes such as TTFF, dwell and revisits.	Eye-Tracking Methods
LR0167	Quantitative non-randomized	3.3 — Complete outcome data	Can't tell	The final valid sample is 42, but the initial number approached or recorded and the full exclusion pathway are not reported, preventing assessment of outcome completeness.	Participants; Data Quality
LR0167	Quantitative non-randomized	3.4 — Confounders accounted for	Yes	Latin-square/randomized presentation, covariate adjustment, mixed-effects models and sensitivity analyses address important order and participant-level confounding.	Design; Statistical Analysis
LR0167	Quantitative non-randomized	3.5 — Intervention/exposure occurred as intended	Yes	All advertisement conditions were presented in the programmed within-subject procedure as intended.	Procedure
LR0172	Quantitative non-randomized	S1 — Clear research questions/objectives	Yes	The report states explicit aims, hypotheses or objectives concerning comparative human responses to the studied AI-generated content in "Evaluating WELL-Informed Biophilic Façades in Automated Retail Environments: A Multimodal Eye-Tracking and Facial Expression Analysis".	Abstract; Introduction / stated aims
LR0172	Quantitative non-randomized	S2 — Collected data address the questions/objectives	Yes	The collected participant data include eye-tracking/gaze outcomes and the relevant behavioral, cognitive, evaluative or physiological measures needed to address the stated comparative objective.	Methods; Results

RefID	MMAT design	Criterion	Rating	Evidence / rationale	Source location
LR0172	Quantitative non-randomized	3.1 — Participants representative of target population	No	The final sample was 86.7% aged 18–25 and was not representative of the diverse users of automated retail environments.	Participants
LR0172	Quantitative non-randomized	3.2 — Measurements appropriate	Yes	The study used calibrated online eye tracking, explicit quality thresholds, facial-expression analysis and a semantic differential scale with clear repeated-measures outcomes.	Multimodal Experiment; Data Processing
LR0172	Quantitative non-randomized	3.3 — Complete outcome data	No	Only 30 of 48 recruited participants were retained, a 37.5% exclusion rate, creating a material risk of incomplete-outcome bias.	Participants / Data Quality
LR0172	Quantitative non-randomized	3.4 — Confounders accounted for	Yes	AI façade variants were based on a common control storefront, presentation was randomized, and repeated-measures/correlation analyses reflected the within-person design.	Stimuli; Procedure; Analysis
LR0172	Quantitative non-randomized	3.5 — Intervention/exposure occurred as intended	Yes	The standardized image set and multimodal recording protocol were administered as planned.	Experimental Procedure
LR0197	Mixed methods	S1 — Clear research questions/objectives	Yes	The report states explicit aims, hypotheses or objectives concerning comparative human responses to the studied AI-generated content in "Measuring Translation Trainees' Effort in AI-Assisted Post-Editing: A Multi-Method Approach".	Abstract; Introduction / stated aims
LR0197	Mixed methods	S2 — Collected data address the questions/objectives	Yes	The collected participant data include eye-tracking/gaze outcomes and the relevant behavioral, cognitive, evaluative or physiological measures needed to address the stated comparative objective.	Methods; Results
LR0197	Mixed methods	1.1 — Qualitative approach appropriate	Yes	Retrospective screen-recording-cued interviews are appropriate for eliciting trainees' reasons for effort, pauses and responses to AI suggestions.	Interview Component
LR0197	Mixed methods	1.2 — Qualitative data collection adequate	Can't tell	The report does not fully specify the interview guide, recording/transcription process or interviewer procedures.	Interview Methods
LR0197	Mixed methods	1.3 — Findings adequately derived from data	No	A formal coding method, theme derivation and auditable analytic process are not sufficiently described.	Qualitative Analysis
LR0197	Mixed methods	1.4 — Interpretation sufficiently substantiated by data	No	Interview interpretations are mostly summarized or counted without adequate quotations or detailed supporting extracts.	Results / Interview Findings
LR0197	Mixed methods	1.5 — Coherence across qualitative sources, collection, analysis and interpretation	Can't tell	The limited reporting prevents confirmation of coherence across qualitative collection, analysis and interpretation.	Methods and Results
LR0197	Mixed methods	3.1 — Participants representative of target population	Yes	Translation trainees were recruited from three MA programs using explicit eligibility criteria and power planning, matching the target population.	Participants
LR0197	Mixed methods	3.2 — Measurements appropriate	Yes	EyeLink 1000+, Translog keylogging, self-ratings and established effort measures are clearly operationalized and appropriate.	Measures and Apparatus
LR0197	Mixed methods	3.3 — Complete outcome data	Yes	All 104 trials were collected; predefined outlier/data-cleaning rules are reported, with no material participant attrition.	Data Processing
LR0197	Mixed methods	3.4 — Confounders accounted for	Yes	Latin-square assignment and linear mixed-effects models with participant and text random effects address order, text and individual confounding.	Design and Statistical Analysis
LR0197	Mixed methods	3.5 — Intervention/exposure occurred as intended	Yes	Traditional and GPT-assisted post-editing conditions were delivered according to the counterbalanced protocol.	Procedure
LR0197	Mixed methods	5.1 — Adequate rationale for mixed methods	Yes	The rationale is to triangulate temporal, technical, cognitive and subjective effort using eye tracking, keylogging, ratings and retrospective interviews.	Study Design
LR0197	Mixed methods	5.2 — Components effectively integrated	Yes	Interview data are used alongside gaze, pause and rating results to explain when and why effort occurred.	Results / Integrated Interpretation
LR0197	Mixed methods	5.3 — Integrated outputs adequately interpreted	Yes	The discussion develops joint interpretations across objective effort indicators and trainees' retrospective accounts.	Discussion
LR0197	Mixed methods	5.4 — Divergences/inconsistencies adequately addressed	Yes	Discrepancies among eye-tracking, pause and self-rating indicators are explicitly discussed and reconciled rather than ignored.	Discussion
LR0197	Mixed methods	5.5 — Each component adheres to its methodological quality criteria	No	The quantitative component is strong, but the qualitative component does not meet criteria for transparent data collection, derivation and substantiation.	Component Appraisals

https://doi.org/10.3390/jemr19050097

RefID	MMAT design	Criterion	Rating	Evidence / rationale	Source location
LR0202	Quantitative randomized controlled trial	S1 — Clear research questions/objectives	Yes	The report states explicit aims, hypotheses or objectives concerning comparative human responses to the studied AI-generated content in “Effects of Pedagogical Agent-Generated Summaries on Video-Based Learning: Evidence from Eye-Tracking and EEG”.	Abstract; Introduction / stated aims
LR0202	Quantitative randomized controlled trial	S2 — Collected data address the questions/objectives	Yes	The collected participant data include eye-tracking/gaze outcomes and the relevant behavioral, cognitive, evaluative or physiological measures needed to address the stated comparative objective.	Methods; Results
LR0202	Quantitative randomized controlled trial	2.1 — Randomization appropriately performed	Yes	The report explicitly describes seeded complete randomization to the mind-map, text-summary and control groups.	Randomization / Participants
LR0202	Quantitative randomized controlled trial	2.2 — Groups comparable at baseline	Yes	Prior knowledge, age and gender were assessed and reported as comparable across groups.	Baseline Comparability
LR0202	Quantitative randomized controlled trial	2.3 — Complete outcome data	Yes	Eighty of 88 datasets were retained (about 90.9%); exclusions are described and the final groups were all analyzed, including delayed outcomes.	Participants / Data Screening
LR0202	Quantitative randomized controlled trial	2.4 — Outcome assessors blinded	No	The visible intervention made participant blinding impossible, self-reported cognitive load was condition-aware, and assessor blinding is not reported.	Procedure; Outcomes
LR0202	Quantitative randomized controlled trial	2.5 — Participants adhered to assigned intervention	Yes	The platform delivered the assigned summary/no-summary condition and constrained video controls consistently.	Experimental Procedure
LR0228	Quantitative non-randomized	S1 — Clear research questions/objectives	Yes	The report states explicit aims, hypotheses or objectives concerning comparative human responses to the studied AI-generated content in “Shifts in Doctors’ Eye Movements Between Real and AI-Generated Medical Images”.	Abstract; Introduction / stated aims
LR0228	Quantitative non-randomized	S2 — Collected data address the questions/objectives	Yes	The collected participant data include eye-tracking/gaze outcomes and the relevant behavioral, cognitive, evaluative or physiological measures needed to address the stated comparative objective.	Methods; Results
LR0228	Quantitative non-randomized	3.1 — Participants representative of target population	Can’t tell	The study involved 16 radiologists, but recruitment, sampling frame, specialty mix and intended target population are insufficiently described.	Participants
LR0228	Quantitative non-randomized	3.2 — Measurements appropriate	Yes	EyeLink 1000 Plus at 500 Hz, 13-point calibration, real and synthetic X-rays, gaze/saliency outcomes and diagnosis/authenticity judgments are appropriate.	Methods / Eye Tracking
LR0228	Quantitative non-randomized	3.3 — Complete outcome data	Can’t tell	The report does not provide a transparent recruitment, exclusion or missing-gaze flow for the radiologist sample.	Participants; Results
LR0228	Quantitative non-randomized	3.4 — Confounders accounted for	No	Experience and specialty differences are not modeled, and the analyses do not clearly account for repeated observations nested within radiologists and images.	Analysis
LR0228	Quantitative non-randomized	3.5 — Intervention/exposure occurred as intended	Yes	Radiologists viewed the planned real and AI-generated image sets under a common protocol.	Procedure
LR0258	Quantitative non-randomized	S1 — Clear research questions/objectives	Yes	The report states explicit aims, hypotheses or objectives concerning comparative human responses to the studied AI-generated content in “The Eyes Know It: FakeET—An Eye-Tracking Database to Understand Deepfake Perception”.	Abstract; Introduction / stated aims
LR0258	Quantitative non-randomized	S2 — Collected data address the questions/objectives	Yes	The collected participant data include eye-tracking/gaze outcomes and the relevant behavioral, cognitive, evaluative or physiological measures needed to address the stated comparative objective.	Methods; Results
LR0258	Quantitative non-randomized	3.1 — Participants representative of target population	No	The 40 participants were largely university-affiliated and do not represent the wider public targeted by deepfake-perception claims.	Participants
LR0258	Quantitative non-randomized	3.2 — Measurements appropriate	Yes	Tobii TX300 at 300 Hz, real/deepfake videos, gaze heatmaps, saliency and EEG provide appropriate measurements for the study objective.	Database Acquisition; Methods
LR0258	Quantitative non-randomized	3.3 — Complete outcome data	Can’t tell	Loss of gaze and EEG recordings is mentioned, but the quantity, distribution and analytic handling of missing data are not reported clearly.	Data Collection; Results
LR0258	Quantitative non-randomized	3.4 — Confounders accounted for	No	Videos had unequal numbers of viewings, easy/difficult labels were partly subjective, and aggregate ANOVA-style analyses do not clearly model participant and video nesting.	Experimental Design; Analysis

RefID	MMAT design	Criterion	Rating	Evidence / rationale	Source location
LR0258	Quantitative non-randomized	3.5 — Intervention/exposure occurred as intended	Yes	Participants were exposed to the planned real and fake video sets while gaze and EEG were recorded.	Procedure
LR0273	Quantitative non-randomized	S1 — Clear research questions/objectives	Yes	The report states explicit aims, hypotheses or objectives concerning comparative human responses to the studied AI-generated content in "Unveiling the Truth: Exploring Human Gaze Patterns in Fake Images".	Abstract; Introduction / stated aims
LR0273	Quantitative non-randomized	S2 — Collected data address the questions/objectives	Yes	The collected participant data include eye-tracking/gaze outcomes and the relevant behavioral, cognitive, evaluative or physiological measures needed to address the stated comparative objective.	Methods; Results
LR0273	Quantitative non-randomized	3.1 — Participants representative of target population	Can't tell	The report states only that 20 participants took part; demographics, recruitment and target population are not described sufficiently.	Eye-Tracking Experiment / Participants
LR0273	Quantitative non-randomized	3.2 — Measurements appropriate	Yes	The calibrated eye-tracking task, real/diffusion-edited pairs and fixation/saliency/entropy outcomes are appropriate to explore gaze differences.	Dataset and Eye-Tracking Method
LR0273	Quantitative non-randomized	3.3 — Complete outcome data	Can't tell	No participant/trial exclusion, data-loss or missing-outcome flow is reported.	Methods; Results
LR0273	Quantitative non-randomized	3.4 — Confounders accounted for	No	The statistical comparison appears to treat multiple observations from the same participants and images as independent rather than modeling the repeated hierarchical structure.	Statistical Analysis
LR0273	Quantitative non-randomized	3.5 — Intervention/exposure occurred as intended	Yes	Real and edited stimuli were presented according to the stated randomized eye-tracking protocol.	Experiment Procedure
LR0280	Quantitative randomized controlled trial	S1 — Clear research questions/objectives	Yes	The report states explicit aims, hypotheses or objectives concerning comparative human responses to the studied AI-generated content in "Comparing the Effects of GenAI and Traditional Search on Scientific Concept Understanding in a Simulation-Based Video Lecture: An Eye-Tracking Study".	Abstract; Introduction / stated aims
LR0280	Quantitative randomized controlled trial	S2 — Collected data address the questions/objectives	Yes	The collected participant data include eye-tracking/gaze outcomes and the relevant behavioral, cognitive, evaluative or physiological measures needed to address the stated comparative objective.	Methods; Results
LR0280	Quantitative randomized controlled trial	2.1 — Randomization appropriately performed	Can't tell	Participants were described as randomly assigned to traditional or GenAI search, but no method for generating or concealing the allocation sequence is provided.	Design / Participants
LR0280	Quantitative randomized controlled trial	2.2 — Groups comparable at baseline	Yes	Prior knowledge and gender proportions did not significantly differ between groups.	Baseline Characteristics
LR0280	Quantitative randomized controlled trial	2.3 — Complete outcome data	Yes	Fifty-seven of 66 participants were retained (86.4%); exclusions for eye-tracking quality or incomplete operations are reported.	Participants / Data Screening
LR0280	Quantitative randomized controlled trial	2.4 — Outcome assessors blinded	No	Participants knew whether they were using Baidu/traditional search or the GenAI interface; self-report and task outcomes were not blinded.	Procedure
LR0280	Quantitative randomized controlled trial	2.5 — Participants adhered to assigned intervention	Yes	Participants used the designated search mode during the lecture task, and free prompting/searching was part of the intended intervention.	Experimental Procedure
LR0317	Quantitative non-randomized	S1 — Clear research questions/objectives	Yes	The report states explicit aims, hypotheses or objectives concerning comparative human responses to the studied AI-generated content in "Attention Allocation in L2 Learners' Writing Feedback: Eye-Tracking Contrasts Between GenAI and Teacher Multidimensional Feedback".	Abstract; Introduction / stated aims
LR0317	Quantitative non-randomized	S2 — Collected data address the questions/objectives	Yes	The collected participant data include eye-tracking/gaze outcomes and the relevant behavioral, cognitive, evaluative or physiological measures needed to address the stated comparative objective.	Methods; Results
LR0317	Quantitative non-randomized	3.1 — Participants representative of target population	No	The sample comprised only 10 Chinese undergraduates at one institution, making it unrepresentative of L2 learners generally.	Participants
LR0317	Quantitative non-randomized	3.2 — Measurements appropriate	Yes	EyeLink 1000 Plus and normalized fixation duration, fixation points and backtracking per word are appropriate to compare feedback sources.	Eye-Tracking Methods
LR0317	Quantitative non-randomized	3.3 — Complete outcome data	Yes	All 10 participants appear in the reported analyses and no missing-data exclusions are described.	Results

https://doi.org/10.3390/jemr19050097

RefID	MMAT design	Criterion	Rating	Evidence / rationale	Source location
LR0317	Quantitative non-randomized	3.4 — Confounders accounted for	No	GenAI feedback was consistently displayed on the left and teacher feedback on the right; feedback length/content and each learner's source text also varied, leaving substantial position and content confounding.	Interface and Procedure
LR0317	Quantitative non-randomized	3.5 — Intervention/exposure occurred as intended	Yes	Both feedback types were delivered within the developed interactive system as intended.	Procedure
LR0321	Quantitative randomized controlled trial	S1 — Clear research questions/objectives	Yes	The report states explicit aims, hypotheses or objectives concerning comparative human responses to the studied AI-generated content in "Generative AI and Multimodal Data for Educational Feedback: Insights from Embodied Math Learning".	Abstract; Introduction / stated aims
LR0321	Quantitative randomized controlled trial	S2 — Collected data address the questions/objectives	Yes	The collected participant data include eye-tracking/gaze outcomes and the relevant behavioral, cognitive, evaluative or physiological measures needed to address the stated comparative objective.	Methods; Results
LR0321	Quantitative randomized controlled trial	2.1 — Randomization appropriately performed	Can't tell	The children were randomly assigned to GenAI or human-instructor feedback, but sequence generation and allocation concealment are not described.	Design / Assignment
LR0321	Quantitative randomized controlled trial	2.2 — Groups comparable at baseline	Can't tell	No adequate baseline equivalence table or statistical comparison between the feedback groups is reported.	Participants / Baseline
LR0321	Quantitative randomized controlled trial	2.3 — Complete outcome data	Yes	Thirty of 34 participants were analyzed after four incomplete multimodal datasets were excluded (about 88.2% retained).	Participants / Data Quality
LR0321	Quantitative randomized controlled trial	2.4 — Outcome assessors blinded	No	Participants and feedback providers knew the source, and no blinded outcome coding/assessment is reported.	Procedure; Analysis
LR0321	Quantitative randomized controlled trial	2.5 — Participants adhered to assigned intervention	Yes	Feedback was delivered according to group assignment during controlled school sessions.	Procedure
LR0334	Quantitative non-randomized	S1 — Clear research questions/objectives	Yes	The report states explicit aims, hypotheses or objectives concerning comparative human responses to the studied AI-generated content in "Eyes Can Tell: Assessment of Implicit Attitudes Toward AI Art".	Abstract; Introduction / stated aims
LR0334	Quantitative non-randomized	S2 — Collected data address the questions/objectives	Yes	The collected participant data include eye-tracking/gaze outcomes and the relevant behavioral, cognitive, evaluative or physiological measures needed to address the stated comparative objective.	Methods; Results
LR0334	Quantitative non-randomized	3.1 — Participants representative of target population	No	The 34 participants were Japanese undergraduates without art expertise; the sample does not represent broader or cross-cultural art audiences.	Participants
LR0334	Quantitative non-randomized	3.2 — Measurements appropriate	Yes	The 300-Hz Tobii procedure, free-viewing task, fixation outcomes and aesthetic/provenance judgments appropriately address the objectives.	Methods
LR0334	Quantitative non-randomized	3.3 — Complete outcome data	Yes	All 34 participants and 1,360 trials appear to have been analyzed.	Results
LR0334	Quantitative non-randomized	3.4 — Confounders accounted for	Yes	AI and human paintings were tested/matched, presentation was randomized, and linear mixed models included participant and painting random intercepts; image-size effects were considered.	Stimuli; Statistical Analysis
LR0334	Quantitative non-randomized	3.5 — Intervention/exposure occurred as intended	Yes	Participants completed the planned viewing, rating and categorization tasks for both origins.	Procedure
LR0386	Quantitative randomized controlled trial	S1 — Clear research questions/objectives	Yes	The report states explicit aims, hypotheses or objectives concerning comparative human responses to the studied AI-generated content in "Unpacking Help-Seeking Process Through Multimodal Learning Analytics: A Comparative Study of ChatGPT vs. Human Expert".	Abstract; Introduction / stated aims
LR0386	Quantitative randomized controlled trial	S2 — Collected data address the questions/objectives	Yes	The collected participant data include eye-tracking/gaze outcomes and the relevant behavioral, cognitive, evaluative or physiological measures needed to address the stated comparative objective.	Methods; Results
LR0386	Quantitative randomized controlled trial	2.1 — Randomization appropriately performed	Can't tell	Random allocation to ChatGPT or human-expert help is stated, but the sequence-generation and concealment method is not reported.	Participants and Procedure
LR0386	Quantitative randomized controlled trial	2.2 — Groups comparable at baseline	Can't tell	The two groups are reported by size, but no baseline comparison of writing ability, demographics or relevant covariates is provided.	Participants / Baseline

RefID	MMAT design	Criterion	Rating	Evidence / rationale	Source location
LR0386	Quantitative randomized controlled trial	2.3 — Complete outcome data	Yes	All 38 participants (18 AI, 20 human expert) appear in the multimodal/process analyses; no attrition is reported.	Participants; Results
LR0386	Quantitative randomized controlled trial	2.4 — Outcome assessors blinded	No	Participants could identify the help source, and coders viewed conversations and screen recordings that revealed condition; blinding is not described.	Coding Process
LR0386	Quantitative randomized controlled trial	2.5 — Participants adhered to assigned intervention	Yes	The online platform provided ChatGPT or human-expert support according to the assigned group throughout revision.	Experiment Procedure
LR0525	Quantitative non-randomized	S1 — Clear research questions/objectives	Yes	The report states explicit aims, hypotheses or objectives concerning comparative human responses to the studied AI-generated content in “Reading the Readers’ Mind Through Eye Tracking: Can AI-Generated Texts Match Human Authors?”.	Abstract; Introduction / stated aims
LR0525	Quantitative non-randomized	S2 — Collected data address the questions/objectives	Yes	The collected participant data include eye-tracking/gaze outcomes and the relevant behavioral, cognitive, evaluative or physiological measures needed to address the stated comparative objective.	Methods; Results
LR0525	Quantitative non-randomized	3.1 — Participants representative of target population	No	Only 13 university participants were recruited and 12 analyzed; this small single-site sample is not representative of general readers.	Participants
LR0525	Quantitative non-randomized	3.2 — Measurements appropriate	Yes	The study used a 90-Hz Tobii device, I2MC event detection, controlled typography/layout and standard reading/gaze outcomes.	Apparatus; Data Processing
LR0525	Quantitative non-randomized	3.3 — Complete outcome data	Yes	The exclusion flow is explicit: one participant and 12 low-quality trials were removed, leaving 12 participants and 66 trials for analysis.	Data Quality / Participants
LR0525	Quantitative non-randomized	3.4 — Confounders accounted for	Yes	A within-subject 6×6 Latin-square design, controlled presentation and participant-aware modeling address order and individual confounding.	Design; Statistical Analysis
LR0525	Quantitative non-randomized	3.5 — Intervention/exposure occurred as intended	Yes	AI and human texts were administered according to the counterbalanced reading protocol.	Procedure
LR0558	Quantitative non-randomized	S1 — Clear research questions/objectives	Yes	The report states explicit aims, hypotheses or objectives concerning comparative human responses to the studied AI-generated content in “AI Images vs. Real Photographs: Investigating Visual Recognition and Perception”.	Abstract; Introduction / stated aims
LR0558	Quantitative non-randomized	S2 — Collected data address the questions/objectives	Yes	The collected participant data include eye-tracking/gaze outcomes and the relevant behavioral, cognitive, evaluative or physiological measures needed to address the stated comparative objective.	Methods; Results
LR0558	Quantitative non-randomized	3.1 — Participants representative of target population	No	The 36 Polish adults were recruited without a clearly justified sampling frame or target-population strategy; representativeness is weak.	Participants
LR0558	Quantitative non-randomized	3.2 — Measurements appropriate	Yes	The study used a 150-Hz GazePoint system, repeated nine-point calibration, recognition responses, gaze patterns and time measures suitable for visual recognition.	Method / Eye Tracking
LR0558	Quantitative non-randomized	3.3 — Complete outcome data	Yes	All 36 participants appear to have completed and contributed data; no attrition is reported.	Participants; Results
LR0558	Quantitative non-randomized	3.4 — Confounders accounted for	No	AI images and real photographs came from different sources, generated images were subjectively selected as the ‘best,’ and low-level visual/category matching was not demonstrated.	Stimulus Selection
LR0558	Quantitative non-randomized	3.5 — Intervention/exposure occurred as intended	Yes	All participants viewed the intended 12 AI and 12 real images under the programmed task.	Procedure
LR0560	Quantitative non-randomized	S1 — Clear research questions/objectives	Yes	The report states explicit aims, hypotheses or objectives concerning comparative human responses to the studied AI-generated content in “Spotting Fakes: How Do Non-Experts Approach Deepfake Video Detection?”.	Abstract; Introduction / stated aims
LR0560	Quantitative non-randomized	S2 — Collected data address the questions/objectives	Yes	The collected participant data include eye-tracking/gaze outcomes and the relevant behavioral, cognitive, evaluative or physiological measures needed to address the stated comparative objective.	Methods; Results
LR0560	Quantitative non-randomized	3.1 — Participants representative of target population	No	The eye-tracking sample of 32 non-experts was recruited through convenience/student/snowball routes and does not represent the general public.	Study 2 Participants

RefID	MMAT design	Criterion	Rating	Evidence / rationale	Source location
LR0560	Quantitative non-randomized	3.2 — Measurements appropriate	Yes	Pupil Invisible eye tracking, dynamic face/body AOIs, authentic/deepfake matched videos and detection outcomes appropriately capture the research question.	Study 2 Method
LR0560	Quantitative non-randomized	3.3 — Complete outcome data	Yes	All 32 Study 2 participants are reported in the eye-tracking analyses, with no attrition or missing-outcome exclusions noted.	Study 2 Results
LR0560	Quantitative non-randomized	3.4 — Confounders accounted for	Yes	Authentic/deepfake versions were matched, video order was randomized, and repeated-measures analyses incorporated AOI, authenticity and decision factors with correction.	Study 2 Design and Analysis
LR0560	Quantitative non-randomized	3.5 — Intervention/exposure occurred as intended	Yes	Participants viewed the assigned authentic/deepfake video set during dynamic eye tracking as intended.	Study 2 Procedure

S10. Eye-Tracking Reporting Completeness

Supplementary Table S10A. Hardware, sampling, calibration, validation, event detection, and AOIs.

RefID	Study	Device/model	Sampling rate	Calibration	Validation/drift	Event algorithm	Numerical event thresholds	AOI/ROI definition
LR0026	Morita et al. (2025)	Partial	Yes	Partial	No	Yes	Yes	Yes
LR0039	Fu et al. (2026)	Yes	Yes	No	No	No	No	Yes
LR0042	Cha et al. (2026)	Yes	Yes	Partial	Partial	No	No	Yes
LR0123	Guan et al. (2025)	Yes	Yes	Yes	Partial	No	No	Yes
LR0125	Abu-Rayyash & Lacruz (2025)	Yes	Yes	Yes	Yes	Yes	Yes	Yes
LR0144	Komninos et al. (2024)	Yes	No	Partial	No	No	No	Yes
LR0152	Bozkir et al. (2024)	Yes	Yes	Yes	No	Yes	Yes	No
LR0166	Liu et al. (2026)	Partial	No	No	No	No	No	Partial
LR0167	Yfantidou et al. (2025)	Yes	No	Yes	Yes	Yes	Yes	Yes
LR0172	Yun & Kim (2026)	Yes	Yes	Yes	Yes	No	No	Yes
LR0197	Yao et al. (2025)	Yes	Yes	Yes	Yes	No	Yes	Yes
LR0202	Yuan et al. (2026)	No	Yes	No	No	No	No	Yes
LR0228	Wong et al. (2025)	Yes	Yes	Yes	Yes	Yes	Yes	No
LR0258	Gupta et al. (2020)	Yes	Yes	No	No	Yes	No	No
LR0273	Cartella et al. (2024)	No	No	Partial	No	Yes	No	No
LR0280	Xu et al. (2026)	Yes	No	Yes	Partial	No	No	Yes
LR0317	Cao et al. (2025)	Yes	No	No	No	No	No	Yes
LR0321	Cosentino et al. (2025)	Partial	Partial	Yes	No	No	No	Yes
LR0334	Zhou & Kawabata (2023)	Yes	Yes	Yes	Yes	Yes	Partial	No
LR0386	Chen et al. (2025)	Yes	No	No	No	N/A	N/A	Yes
LR0525	Ilyas et al. (2025)	Yes	Yes	Partial	Yes	Yes	Yes	No
LR0558	Osińska et al. (2025)	Yes	Yes	Yes	No	Partial	No	Yes
LR0560	Holmes et al. (2025)	Yes	No	Partial	No	No	No	Yes

Supplementary Table S10B. Preprocessing, missing data, exclusions, pupil processing, visual controls, multiplicity, and data availability.

RefID	Study	Preprocessing	Missing-data reporting	Participant/trial exclusion rules	Pupil preprocessing	Luminance/visual control	Multiplicity/statistical correction	Data/code availability	Reporting note
LR0026	Morita et al. (2025)	Partial	No	No	N/A	Partial	No	Partial	Model NR; no formal track-loss rule; custom I-DT thresholds were reported.

RefID	Study	Preprocessing	Missing-data reporting	Participant/trial exclusion rules	Pupil preprocessing	Luminance/visual control	Multiplicity/statistical correction	Data/code availability	Reporting note
LR0039	Fu et al. (2026)	Yes	Partial	Partial	Yes	Partial	Partial	Partial	Pupil baseline correction was reported, but calibration/event-detection details were absent.
LR0042	Cha et al. (2026)	Partial	Partial	Partial	Partial	Yes	Yes	Partial	High-frequency recording and visual matching were strong; fixation detection remained underreported.
LR0123	Guan et al. (2025)	Partial	Partial	Partial	N/A	Yes	Yes	Partial	Nine-point calibration and attrition were reported; low-sampling exclusion threshold was not.
LR0125	Abu-Rayyash & Lacruz (2025)	Yes	Yes	Yes	N/A	Yes	Yes	No	Most complete ET reporting in the corpus, including calibration, equipment checks, I-VT and quality classes.
LR0144	Komninos et al. (2024)	No	No	No	N/A	Yes	Partial	Yes	Device and AOIs were clear; sampling, event detection and quality thresholds were not.
LR0152	Bozkir et al. (2024)	Yes	Partial	Partial	N/A	Yes	Partial	Partial	Detailed I-VT/interpolation/smoothing; no formal AOIs or numerical participant-quality cutoff.
LR0166	Liu et al. (2026)	No	No	No	N/A	Partial	Partial	Partial	Device model, sampling, calibration and fixation processing were largely absent.
LR0167	Yfantidou et al. (2025)	Yes	Yes	Yes	N/A	Partial	Yes	Partial	Highly detailed remote-ET calibration, drift, event and track-loss rules.
LR0172	Yun & Kim (2026)	Yes	Yes	Yes	N/A	Yes	Partial	Partial	Explicit webcam accuracy/completion thresholds; event algorithm NR.
LR0197	Yao et al. (2025)	Yes	Partial	Yes	N/A	Yes	Partial	Partial	Strong calibration/drift/preprocessing; event algorithm itself NR.
LR0202	Yuan et al. (2026)	No	Partial	Partial	N/A	Yes	Partial	No	Sampling and AOIs reported, but device, calibration, filter and preprocessing omitted.
LR0228	Wong et al. (2025)	Yes	No	No	N/A	Yes	Partial	No	Strong hardware/calibration/event thresholds; missing-data/exclusion reporting weak.
LR0258	Gupta et al. (2020)	Partial	Partial	No	N/A	Partial	Partial	Yes	Sampling and event method reported; calibration/threshold and missing-data handling incomplete.
LR0273	Cartella et al. (2024)	Yes	No	No	N/A	Yes	Partial	Yes	Paired design/data public, but tracker, sampling and event threshold omitted.
LR0280	Xu et al. (2026)	Yes	Yes	Partial	N/A	Yes	Partial	No	Calibration and cleaning described qualitatively; sampling and numerical validity thresholds NR.
LR0317	Cao et al. (2025)	Partial	No	No	N/A	No	Partial	No	Device and AOIs reported; most ET technical details absent.
LR0321	Cosentino et al. (2025)	Yes	Yes	Partial	Yes	Yes	Partial	Partial	Good mapping/pupil preprocessing, but device model/event filter unclear and sampling statements inconsistent.
LR0334	Zhou & Kawabata (2023)	Partial	Yes	Partial	N/A	Yes	Yes	Partial	Strong calibration/validation and filter naming; exact velocity threshold not provided.
LR0386	Chen et al. (2025)	Yes	No	No	N/A	Yes	Partial	No	Gaze used for process coding rather than fixation-event analysis; quality thresholds absent.
LR0525	Ilyas et al. (2025)	Yes	Yes	Yes	Partial	Partial	Partial	No	Strong I2MC preprocessing and explicit trial/participant exclusions; pupil normalization limited.
LR0558	Osińska et al. (2025)	Yes	No	No	N/A	No	Partial	Yes	Device/sampling/calibration and AOIs clear; event and quality thresholds absent.

https://doi.org/10.3390/jemr19050097

RefID	Study	Preprocessing	Missing-data reporting	Participant/trial exclusion rules	Pupil preprocessing	Luminance/visual control	Multiplicity/statistical correction	Data/code availability	Reporting note
LR0560	Holmes et al. (2025)	Yes	No	No	N/A	Yes	Yes	Yes	Dynamic AOIs and open materials were strengths; sampling/filter/quality criteria absent from main article.

Yes = sufficiently reported; Partial = incompletely reported; No = absent/insufficient; N/A = not applicable. This is a purpose-built reporting framework.

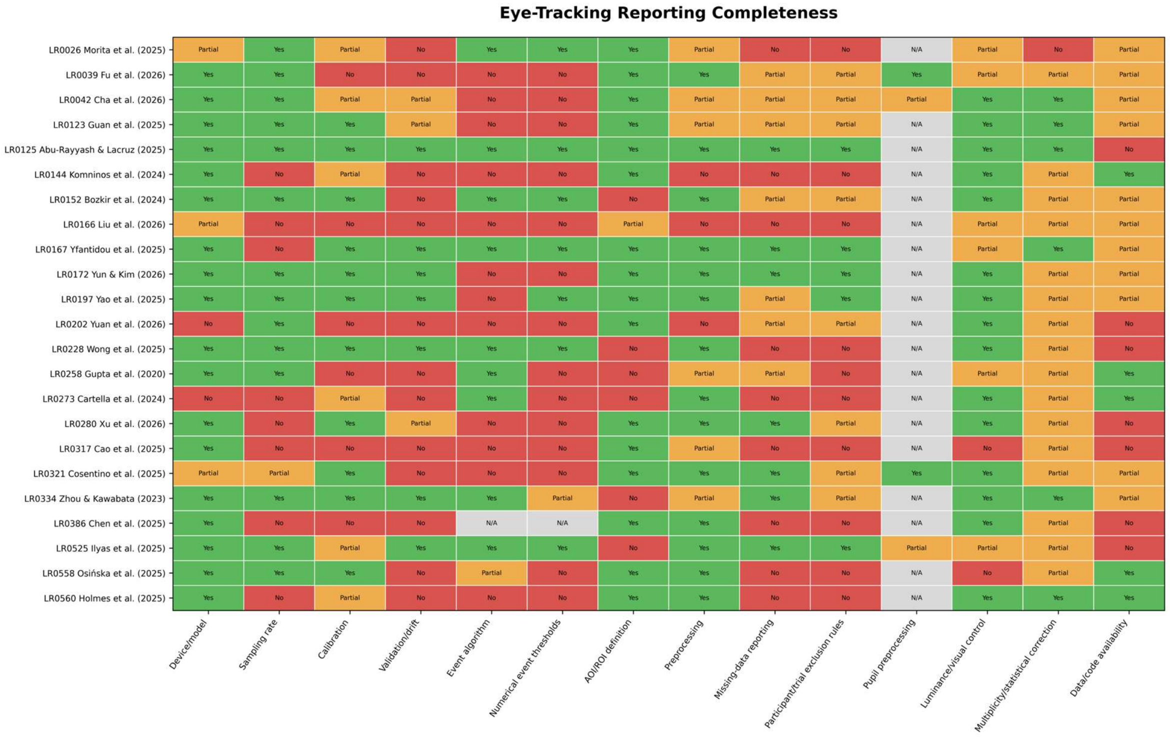

Figure S1. Eye-tracking reporting-completeness heatmap across the 23 included studies. Green indicates sufficiently reported information, amber indicates partial reporting, red indicates absent or insufficient reporting, and grey indicates not applicable.

S11. Reproducibility of AI-Generated Stimuli

Supplementary Table S11A. Model, version, prompt, settings, generation date, and candidate count.

RefID	Study	Model/provider named	Version/snapshot	Prompt/template	Generation settings	Generation date	Candidate/output count
LR0026	Morita et al. (2025)	Yes	Partial	Yes	Partial	No	Partial
LR0039	Fu et al. (2026)	Yes	Partial	Partial	No	No	No
LR0042	Cha et al. (2026)	Yes	Yes	Partial	Partial	No	Partial
LR0123	Guan et al. (2025)	Yes	Yes	Yes	Partial	No	Yes
LR0125	Abu-Rayyash & Lacruz (2025)	Yes	Yes	Partial	Partial	Yes	N/A
LR0144	Komninos et al. (2024)	Yes	Yes	Yes	Partial	No	Yes
LR0152	Bozkir et al. (2024)	Yes	Yes	N/A	N/A	N/A	N/A
LR0166	Liu et al. (2026)	Yes	Yes	Yes	Partial	No	Yes
LR0167	Yfantidou et al. (2025)	No	No	No	No	No	No
LR0172	Yun & Kim (2026)	Yes	Yes	Partial	Partial	No	Partial

RefID	Study	Model/provider named	Version/snapshot	Prompt/template	Generation settings	Generation date	Candidate/output count
LR0197	Yao et al. (2025)	Yes	Partial	Yes	No	No	N/A
LR0202	Yuan et al. (2026)	Yes	No	No	No	No	No
LR0228	Wong et al. (2025)	Yes	Yes	N/A	Yes	No	Partial
LR0258	Gupta et al. (2020)	Partial	No	N/A	N/A	N/A	N/A
LR0273	Cartella et al. (2024)	Yes	Yes	Partial	Partial	No	N/A
LR0280	Xu et al. (2026)	Yes	Yes	Partial	Partial	No	N/A
LR0317	Cao et al. (2025)	Yes	Yes	Partial	Partial	No	N/A
LR0321	Cosentino et al. (2025)	Yes	Partial	Yes	Partial	No	N/A
LR0334	Zhou & Kawabata (2023)	Yes	Partial	No	No	No	No
LR0386	Chen et al. (2025)	Yes	Partial	Partial	No	No	N/A
LR0525	Ilyas et al. (2025)	Yes	Partial	Yes	No	No	No
LR0558	Osińska et al. (2025)	Yes	Partial	No	No	No	Partial
LR0560	Holmes et al. (2025)	Partial	No	N/A	N/A	N/A	N/A

Supplementary Table S11B. Selection, editing, matching, availability, and reproducibility notes.

RefID	Study	Selection/curation procedure	Human editing/post-processing	Comparator matching	Stimuli/output availability	Data/code availability	Reproducibility note	DOI
LR0026	Morita et al. (2025)	Yes	No	Partial	Partial	Partial	Generation pipelines and prompts were illustrated; exact model snapshots/date and human editing remained unclear.	https://doi.org/10.1145/3745900.3746066
LR0039	Fu et al. (2026)	Partial	Partial	Partial	No	Partial	GPT-4.1/Midjourney named; full prompts, settings, candidate selection and stimuli were not fully reproducible.	https://doi.org/10.1016/j.displa.2025.103275
LR0042	Cha et al. (2026)	Yes	Partial	Yes	Yes	Partial	Midjourney v6.1, reference-image procedure, expert curation and low-level matching were well documented.	https://doi.org/10.3389/fnhum.2026.1723608
LR0123	Guan et al. (2025)	Yes	Yes	Yes	Yes	Partial	Detailed multi-stage generation, candidate ranking, professional screening and Photoshop standardization; date/seed absent.	https://doi.org/10.3389/fcomm.2025.1667077
LR0125	Abu-Rayyash & Lacruz (2025)	No	Yes	Yes	No	No	GPT-4o (March 2025) and no post-editing reported; exact prompt/settings and outputs not openly available.	https://doi.org/10.3390/jemr18040029
LR0144	Komninos et al. (2024)	Yes	Partial	Yes	Yes	Yes	Llama 3 8B prompt templates, selection pipeline, phrase sets, code and materials were openly documented.	https://doi.org/10.1145/3701571.3701586
LR0152	Bozkir et al. (2024)	Yes	Yes	Yes	Yes	Partial	Named public GAN datasets/architectures and balanced stimulus selection; prompt/date not applicable.	https://doi.org/10.1145/3696667
LR0166	Liu et al. (2026)	Yes	Partial	Yes	Yes	Partial	Model/version, prompt logic, candidate pool and rejection/selection process	https://doi.org/10.3390/electronics15010015

RefID	Study	Selection/curation procedure	Human editing/post-processing	Comparator matching	Stimuli/output availability	Data/code availability	Reproducibility note	DOI
							were detailed; exact generation settings/date absent.	
LR0167	Yfantidou et al. (2025)	Partial	Partial	Partial	Partial	No	Specific generation model, version, prompts and settings were not reported.	https://doi.org/10.3390/info16110935
LR0172	Yun & Kim (2026)	Partial	Partial	Yes	Yes	Partial	SDXL/ControlNet and common-base generation described; prompts/settings/candidate selection only partly reproducible.	https://doi.org/10.3390/buildings16040876
LR0197	Yao et al. (2025)	N/A	N/A	Yes	Partial	Partial	Detailed GPT task prompt/brief and Latin-square texts; exact GPT snapshot/settings and dynamic outputs not fully available.	https://doi.org/10.1080/1750399X.2025.2535239
LR0202	Yuan et al. (2026)	Partial	Partial	Yes	No	No	Doubao identified and expert validation described, but prompt, model version, settings and stimuli availability were weak.	https://doi.org/10.3390/educsci16010039
LR0228	Wong et al. (2025)	Yes	No	Yes	Partial	No	RoentGen, report conditioning, resolution and inference steps were documented; open stimuli/data unclear.	https://doi.org/10.1145/3715669.3726789
LR0258	Gupta et al. (2020)	Yes	N/A	Partial	Yes	Yes	DFDC/Jigsaw source and public FakeET resource available, but exact deepfake generation models were unknown.	https://doi.org/10.1145/3382507.3418857
LR0273	Cartella et al. (2024)	Yes	N/A	Yes	Yes	Yes	Named models, paired editing pipeline and public dataset/code; prompts/settings only partly specified.	https://doi.org/10.1109/LSP.2024.3375288
LR0280	Xu et al. (2026)	N/A	No	Yes	No	No	GLM-4/Qwen2/RAG architecture reported; dynamic prompts/outputs, settings and snapshots not archived.	https://doi.org/10.3390/educsci16050769
LR0317	Cao et al. (2025)	N/A	Partial	Partial	Partial	No	DeepSeek-R1 and pedagogical constraints/examples described; exact prompt/output archive unavailable.	https://doi.org/10.1109/ICET67421.2025.11380530
LR0321	Cosentino et al. (2025)	N/A	Partial	Yes	Partial	Partial	GPT-4 API and system prompt code were transparent; model snapshot, temperature/date and complete outputs were not.	https://doi.org/10.1111/bjet.13587
LR0334	Zhou & Kawabata (2023)	Partial	No	Partial	Partial	Partial	Disco Diffusion named, but prompts, settings and generation/selection details were sparse.	https://doi.org/10.1177/20416695231209846
LR0386	Chen et al. (2025)	N/A	No	Yes	No	No	ChatGPT '4.0' and contextual rubric reported; exact snapshot/settings and conversations were not openly archived.	https://doi.org/10.1016/j.compedu.2024.105198
LR0525	Ilyas et al. (2025)	No	No	Partial	Partial	No	Full Copilot prompts were available, but backend model snapshot/settings and selection/editing details were not.	https://doi.org/10.1145/3715669.3726846
LR0558	Osińska et al. (2025)	Yes	No	No	Yes	Partial	GPT-4o/ThisPersonDoesNotExist and public	https://doi.org/10.3390/jemr18060061

RefID	Study	Selection/curation procedure	Human editing/post-processing	Comparator matching	Stimuli/output availability	Data/code availability	Reproducibility note	DOI
							stimuli reported; prompts/settings absent and comparator matching weak.	
LR0560	Holmes et al. (2025)	Yes	N/A	Yes	Yes	Yes	Matched DFDC stimuli and OSF materials available; exact generation model not reported.	https://doi.org/10.5771/2192-4007-2025-4-550

Prompt-related items were coded N/A where fixed public GAN/deepfake datasets were used.

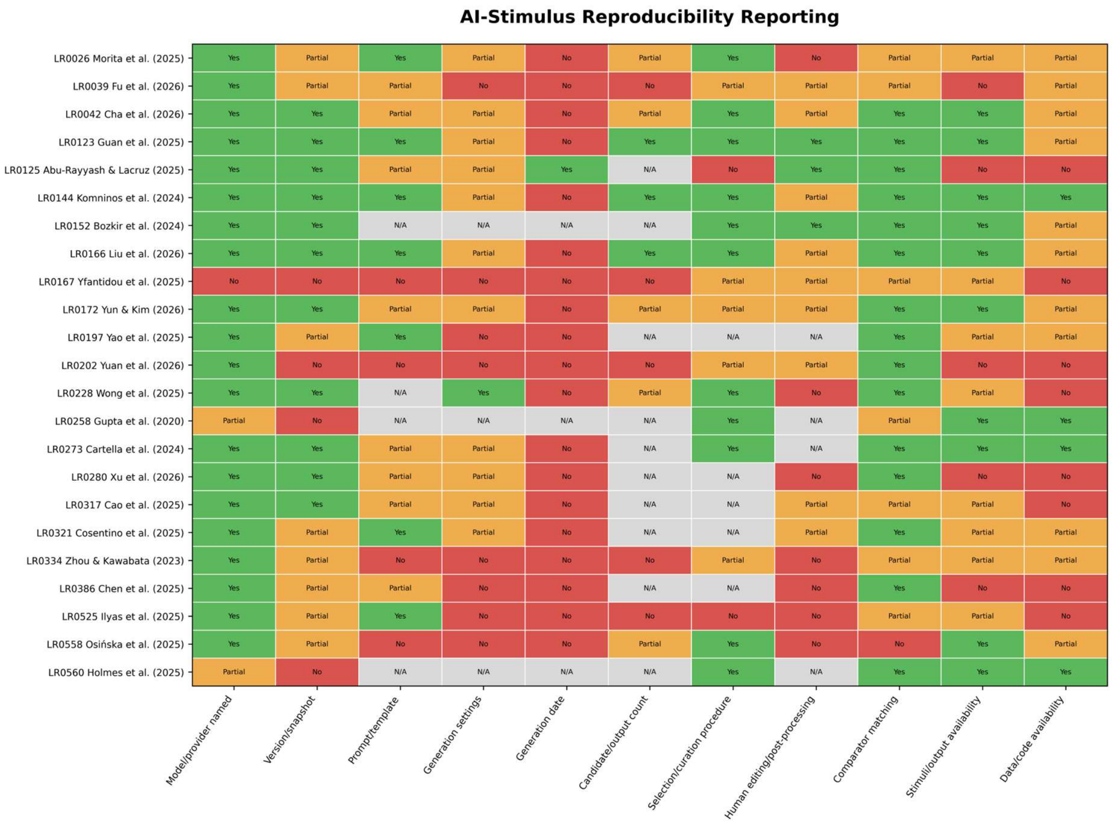

Figure S2. AI-stimulus reproducibility heatmap across the 23 included studies. Green indicates sufficiently reported information, amber indicates partial reporting, red indicates absent or insufficient reporting, and grey indicates not applicable.

S12. Evidence Map

Supplementary Table S12. Study-by-domain evidence map.

RefID	Study	Textual	Static visual	AI video/deepfake	Interactive/task-embedded	Initial attention	General visual-attention evidence	Verification/re-inspection evidence	Scanning/gaze-organization evidence	Formal load/effort	Learning/integration	Authenticity/detection	Professional/creative
LR0026	Morita et al. (2025)	Yes	Yes	No	Yes	No	Yes	Yes	Yes	No	Yes	No	No

RefID	Study	Textual	Static visual	AI video/deepfake	Interactive/task-embedded	Initial attention	General visual-attention evidence	Verification/re-inspection evidence	Scanning/gaze-organization evidence	Formal load/effort	Learning/integration	Authenticity/detection	Professional/creative
LR0039	Fu et al. (2026)	Yes	Yes	No	Yes	No	Yes	Yes	Yes	Yes	No	No	Yes
LR0042	Cha et al. (2026)	No	Yes	No	No	No	Yes	No	No	No	No	Yes	No
LR0123	Guan et al. (2025)	No	Yes	No	No	Yes	Yes	Yes	Yes	No	No	No	Yes
LR0125	Abu-Rayyash & Lacruz (2025)	Yes	No	No	No	Yes	Yes	Yes	Yes	Yes	No	No	No
LR0144	Komninos et al. (2024)	Yes	No	No	Yes	No	Yes	No	No	No	No	No	No
LR0152	Bozkir et al. (2024)	No	Yes	No	No	No	Yes	Yes	Yes	Yes	No	Yes	No
LR0166	Liu et al. (2026)	No	Yes	No	No	No	Yes	Yes	Yes	No	No	Yes	No
LR0167	Yfantidou et al. (2025)	No	Yes	No	No	Yes	Yes	Yes	Yes	No	No	No	Yes
LR0172	Yun & Kim (2026)	No	Yes	No	No	No	Yes	Yes	Yes	No	No	No	Yes
LR0197	Yao et al. (2025)	Yes	No	No	Yes	No	Yes	Yes	No	Yes	No	No	Yes
LR0202	Yuan et al. (2026)	Yes	Yes	No	Yes	No	Yes	Yes	Yes	Yes	Yes	No	No
LR0228	Wong et al. (2025)	No	Yes	No	No	Yes	Yes	Yes	Yes	No	No	Yes	No
LR0258	Gupta et al. (2020)	No	No	Yes	No	No	Yes	Yes	Yes	No	No	Yes	No
LR0273	Cartella et al. (2024)	No	Yes	No	No	No	Yes	No	Yes	No	No	Yes	No
LR0280	Xu et al. (2026)	Yes	No	No	Yes	No	Yes	Yes	Yes	Yes	Yes	No	No
LR0317	Cao et al. (2025)	Yes	No	No	Yes	No	Yes	Yes	Yes	No	Yes	No	No
LR0321	Cosentino et al. (2025)	Yes	No	No	Yes	No	Yes	Yes	Yes	Yes	Yes	No	No
LR0334	Zhou & Kawabata (2023)	No	Yes	No	No	No	Yes	No	No	No	No	Yes	No
LR0386	Chen et al. (2025)	Yes	No	No	Yes	Yes	Yes	Yes	Yes	No	Yes	No	No
LR0525	Ilyas et al. (2025)	Yes	No	No	No	No	Yes	Yes	Yes	No	No	No	No
LR0558	Osińska et al. (2025)	No	Yes	No	No	No	Yes	Yes	Yes	No	No	Yes	No
LR0560	Holmes et al. (2025)	No	No	Yes	No	No	Yes	No	Yes	No	No	Yes	No

Content categories are cross-cutting and should not be summed. "Yes" indicates contribution to the evidence domain, not a positive effect.

Supplementary Abbreviations and Notes

AI, artificial intelligence; AIGC, artificial-intelligence-generated content; AOI, area of interest; ET, eye tracking; I-DT, identification by dispersion threshold; I-VT, identification by velocity threshold; LLM, large language model; MMAT, Mixed Methods Appraisal Tool; NR, not reported; PECOS, Population, Exposure, Comparator, Outcomes, and Study design; PRISMA, Preferred Reporting Items for Systematic Reviews and Meta-Analyses; RAG, retrieval-augmented generation; ROI, region of interest; TTFF, time to first fixation.

Open items requiring author confirmation: final search date; exact exported database queries and filters; citation-level full-text exclusion log; screening, extraction, and appraisal reviewer roles; inter-reviewer agreement; protocol/OSF status and URL; author, funding, conflict-of-interest, and repository details.

S13. PRISMA 2020 Checklist

Table S13. Completed PRISMA 2020 Checklist. This appendix is distinct from Supplementary Tables S3A–S3B, which document full-text exclusions. This checklist follows the PRISMA 2020 statement [25].

Section and Topic	Item #	Checklist item	Location where item is reported
**TITLE**			
Title	1	Identify the report as a systematic review.	Title page, p. 1
**ABSTRACT**			
Abstract	2	See the PRISMA 2020 for Abstracts checklist.	Abstract, p. 1.
**INTRODUCTION**			
Rationale	3	Describe the rationale for the review in the context of existing knowledge.	Section 1.1, "Background and Rationale."
Objectives	4	Provide an explicit statement of the objective(s) or question(s) the review addresses.	Section 1.2, "Objectives and Scope of the Review."
**METHODS**			
Eligibility criteria	5	Specify the inclusion and exclusion criteria for the review and how studies were grouped for the syntheses.	Sections 3.1 and 3.3; Supplementary Table S1. Synthesis grouping is described in Section 3.7 and Sections 4.2–4.8.
Information sources	6	Specify all databases, registers, websites, organisations, reference lists and other sources searched or consulted to identify studies. Specify the date when each source was last searched or consulted.	Section 3.2; Supplementary Table S2A. Sources: Scopus, Web of Science Core Collection, and PubMed; final search date: 2 July

Section and Topic	Item #	Checklist item	Location where item is reported
			2026; limits: English language and 2015–2026.
Search strategy	7	Present the full search strategies for all databases, registers and websites, including any filters and limits used.	Section 3.2 and Supplementary Table S2B. Two Scopus search strings are supplied.
Selection process	8	Specify the methods used to decide whether a study met the inclusion criteria of the review, including how many reviewers screened each record and each report retrieved, whether they worked independently, and if applicable, details of automation tools used in the process.	Section 3.4 and Figure 1 report the selection flow, and Section 3.2
Data collection process	9	Specify the methods used to collect data from reports, including how many reviewers collected data from each report, whether they worked independently, any processes for obtaining or confirming data from study investigators, and if applicable, details of automation tools used in the process.	Section 3.5 describes the standardized extraction framework and the unit of extraction.
Data items	10a	List and define all outcomes for which data were sought. Specify whether all results that were compatible with each outcome domain in each study were sought (e.g. for all measures, time points, analyses), and if not, the methods used to decide which results to collect.	Section 3.5; Sections 4.5–4.8; Supplementary Tables S6A–S7B.
	10b	List and define all other variables for which data were sought (e.g. participant and intervention characteristics, funding sources). Describe any assumptions made about any missing or unclear information.	Section 3.5; Supplementary Tables S4A–S5B, S8A–S8B, and S11A–S11B.

Section and Topic	Item #	Checklist item	Location where item is reported
Study risk of bias assessment	11	Specify the methods used to assess risk of bias in the included studies, including details of the tool(s) used, how many reviewers assessed each study and whether they worked independently, and if applicable, details of automation tools used in the process.	Section 3.6; Supplementary Tables S9A–S11B.
Effect measures	12	Specify for each outcome the effect measure(s) (e.g. risk ratio, mean difference) used in the synthesis or presentation of results.	No common effect measure was used because meta-analysis was not performed. Original-study metrics are summarized by metric family in Section 3.7, Section 4.5, Tables 2–4, and Supplementary Tables S6A–S7B.
Synthesis methods	13a	Describe the processes used to decide which studies were eligible for each synthesis (e.g. tabulating the study intervention characteristics and comparing against the planned groups for each synthesis (item #5)).	Section 3.7; Sections 4.2–4.8; Supplementary Tables S7A–S8B.
	13b	Describe any methods required to prepare the data for presentation or synthesis, such as handling of missing summary statistics, or data conversions.	Section 3.7.
	13c	Describe any methods used to tabulate or visually display results of individual studies and syntheses.	Tables 1–4; Figures 1–2; Sections 4.1–4.10; Supplementary Tables S4–S12 and Supplementary Figures S1–

Section and Topic	Item #	Checklist item	Location where item is reported
			S2.
	13d	Describe any methods used to synthesize results and provide a rationale for the choice(s). If meta-analysis was performed, describe the model(s), method(s) to identify the presence and extent of statistical heterogeneity, and software package(s) used.	Section 3.7.
	13e	Describe any methods used to explore possible causes of heterogeneity among study results (e.g. subgroup analysis, meta-regression).	Sections 3.7, 4.2–4.9, and 5.2.2; Supplementary Tables S8A–S8B.
	13f	Describe any sensitivity analyses conducted to assess robustness of the synthesized results.	Section 3.6 and Section 4.10.2.1.
Reporting bias assessment	14	Describe any methods used to assess risk of bias due to missing results in a synthesis (arising from reporting biases).	Section 5.7.
Certainty assessment	15	Describe any methods used to assess certainty (or confidence) in the body of evidence for an outcome.	Not formally assessed.
**RESULTS**			
Study selection	16a	Describe the results of the search and selection process, from the number of records identified in the search to the number of studies included in the review, ideally using a flow diagram.	Section 3.4 and Figure and Section 4.1.
	16b	Cite studies that might appear to meet the inclusion criteria, but which were excluded, and explain why they were excluded.	Figure 1 and Supplementary Table S3 report aggregate exclusion reasons. The separate “Supplementary Table S3. Citation-level list…” identifies all 50 excluded reports.
Study characteristics	17	Cite each included study and present its characteristics.	Section 4.1; Tables 1–2; Supplementary Tables S4A–S4B.

Section and Topic	Item #	Checklist item	Location where item is reported
Risk of bias in studies	18	Present assessments of risk of bias for each included study.	Sections 4.10.1–4.10.3; Supplementary Tables S9A–S11B and Supplementary Figures S1–S2.
Results of individual studies	19	For all outcomes, present, for each study: (a) summary statistics for each group (where appropriate) and (b) an effect estimate and its precision (e.g. confidence/credible interval), ideally using structured tables or plots.	Sections 4.6–4.8, Table 2, and Supplementary Tables S6A–S7B
Results of syntheses	20a	For each synthesis, briefly summarise the characteristics and risk of bias among contributing studies.	Sections 4.6–4.10; Tables 3–4; Section 4.10.2.1; Supplementary Tables S7–S12.
	20b	Present results of all statistical syntheses conducted. If meta-analysis was done, present for each the summary estimate and its precision (e.g. confidence/credible interval) and measures of statistical heterogeneity. If comparing groups, describe the direction of the effect.	Not applicable
	20c	Present results of all investigations of possible causes of heterogeneity among study results.	Sections 4.2–4.9 and 5.2.2; Supplementary Tables S8A–S8B.
	20d	Present results of all sensitivity analyses conducted to assess the robustness of the synthesized results.	Section 4.10.2.1.
Reporting biases	21	Present assessments of risk of bias due to missing results (arising from reporting biases) for each synthesis assessed.	Not formally assessed.
Certainty of evidence	22	Present assessments of certainty (or confidence) in the body of evidence for each outcome assessed.	Not assessed.
**DISCUSSION**			
Discussion	23a	Provide a general interpretation of the results in the context of other evidence.	Sections 5.1–5.2 and Section 6.
	23b	Discuss any limitations of the evidence included in the review.	Sections 5.3–5.4

Section and Topic	Item #	Checklist item	Location where item is reported
			and 5.7.
	23c	Discuss any limitations of the review processes used.	Section 5.7.
	23d	Discuss implications of the results for practice, policy, and future research.	Sections 5.5–5.6 and Section 6.
**OTHER INFORMATION**			
Registration and protocol	24a	Provide registration information for the review, including register name and registration number, or state that the review was not registered.	Not reported
	24b	Indicate where the review protocol can be accessed, or state that a protocol was not prepared.	Not reported
	24c	Describe and explain any amendments to information provided at registration or in the protocol.	Not applicable as currently reported.
Support	25	Describe sources of financial or non-financial support for the review, and the role of the funders or sponsors in the review.	Funding statement in the back matter: “This research received no external funding.” The role of funders is therefore not applicable.
Competing interests	26	Declare any competing interests of review authors.	Conflicts of Interest statement in the back matter: “The authors declare no conflicts of interest.”
Availability of data, code and other materials	27	Report which of the following are publicly available and where they can be found: template data collection forms; data extracted from included studies; data used for all analyses; analytic code; any other materials used in the review.	Supplementary Materials statement and Data Availability Statement in the back matter; Supplementary Tables

Section and Topic	Item #	Checklist item	Location where item is reported
			S1–S12 and Supplementary Figures S1–S2.

From: Page MJ, McKenzie JE, Bossuyt PM, Boutron I, Hoffmann TC, Mulrow CD, et al. The PRISMA 2020 statement: an updated guideline for reporting systematic reviews. BMJ 2021;372:n71. doi: 10.1136/bmj.n71. This work is licensed under CC BY 4.0. To view a copy of this license, visit https://creativecommons.org/licenses/by/4.0/
